# Soft Electronics for Health Monitoring Assisted by Machine Learning

**DOI:** 10.1007/s40820-023-01029-1

**Published:** 2023-03-15

**Authors:** Yancong Qiao, Jinan Luo, Tianrui Cui, Haidong Liu, Hao Tang, Yingfen Zeng, Chang Liu, Yuanfang Li, Jinming Jian, Jingzhi Wu, He Tian, Yi Yang, Tian-Ling Ren, Jianhua Zhou

**Affiliations:** 1 School of Biomedical Engineering, Shenzhen Campus of Sun Yat-sen University, No. 66, Gongchang Road, Guangming District, Shenzhen, 518107 People’s Republic of China; 2 School of Integrated Circuits and Beijing National Research Center for Information Science and Technology (BNRist), Tsinghua University, Beijing, 100084 People’s Republic of China; 3 Key Laboratory of Sensing Technology and Biomedical Instruments of Guangdong Province, School of Biomedical Engineering, Sun Yat-sen University, Guangzhou, 510275 People’s Republic of China

**Keywords:** Soft electronics, Machine learning algorithm, Physiological signal monitoring, Soft materials

## Abstract

Due to the development of the novel materials, the past two decades have witnessed the rapid advances of soft electronics. The soft electronics have huge potential in the physical sign monitoring and health care. One of the important advantages of soft electronics is forming good interface with skin, which can increase the user scale and improve the signal quality. Therefore, it is easy to build the specific dataset, which is important to improve the performance of machine learning algorithm. At the same time, with the assistance of machine learning algorithm, the soft electronics have become more and more intelligent to realize real-time analysis and diagnosis. The soft electronics and machining learning algorithms complement each other very well. It is indubitable that the soft electronics will bring us to a healthier and more intelligent world in the near future. Therefore, in this review, we will give a careful introduction about the new soft material, physiological signal detected by soft devices, and the soft devices assisted by machine learning algorithm. Some soft materials will be discussed such as two-dimensional material, carbon nanotube, nanowire, nanomesh, and hydrogel. Then, soft sensors will be discussed according to the physiological signal types (pulse, respiration, human motion, intraocular pressure, phonation, etc.). After that, the soft electronics assisted by various algorithms will be reviewed, including some classical algorithms and powerful neural network algorithms. Especially, the soft device assisted by neural network will be introduced carefully. Finally, the outlook, challenge, and conclusion of soft system powered by machine learning algorithm will be discussed.

## Highlights

- This review introduces soft electronics for health monitoring assisted by machine learning, and discusses soft materials, physiological signals, and machine learning algorithms in sequence and their relationships.
- The principles of classic machine learning algorithms and neural network algorithms are summarized and explained by representative examples combining with soft electronics.
- The potential challenges of soft electronics assisted by machine learning especially in health monitoring field are outlined, and future research directions are outlooked.

## Graphical abstract

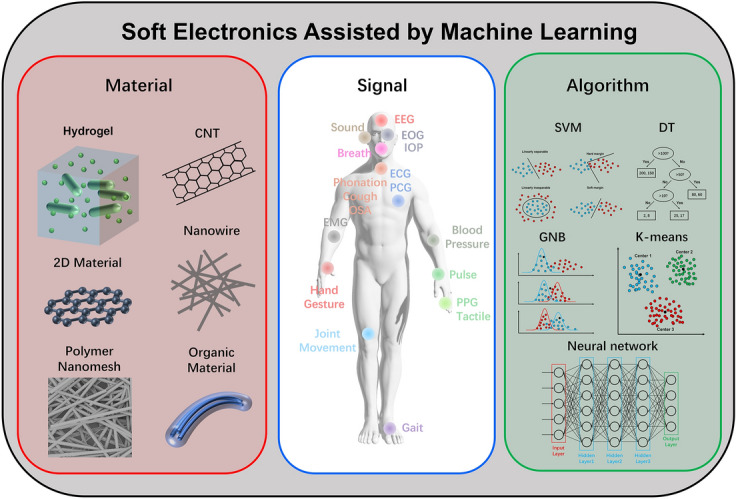

## Introduction

Soft electronics have wide applications in radio frequency identification (RFID) [1], soft display [2], organic light emitting diode (OLED) display and lighting [3], chemical and biological sensors [4], soft photovoltaic [5], soft logic and storage [6], soft battery [7], wearable health monitoring devices [8] and other applications. With the rapid development of soft material and health care requirement, the soft electronic is being paid more and more attentions.

Traditional rigid sensors based on the silicon or other material have some disadvantages such as rigid substrate, no strain, low biocompatibility, which make it not suitable to be used in the large-strain and rough surface conditions [9]. The rigid morphology will greatly influence the wearing experience of the users, no more than long-time wearing. In addition, due to the low Young’s modulus of skin and other organs, the rigid electronics device cannot realize a tight interface with the skin. The air gap in the interface will greatly decrease the signal-to-noise ratio (SNR), introduce motion artifacts, and even destroy the original signal. The soft electronics with the good interface to skin can optimize the wearing experience and expand the number of users, which will enlarge the database size. Besides, more accurate physiological information can be distinguished from the high-quality signal. For the machine learning algorithms, the quantity and quality of data is important, which can help the algorithm to find the law in the data better. Therefore, soft electronics can improve the performance of the machine learning algorithms.

The machine learning algorithms can be used in data information and mining, pattern recognition, bioinformatics, etc., which can make the soft electronics more intelligent. The soft electronic device can monitor the physiological signal in real time and long time. After that, a dataset containing a great deal of physiological information with high quality to be learned and analyzed by machine learning algorithm will be built. Like human learning process, the more we learned, the more knowledge we will obtain. Large-quantity dataset can provide more knowledge to the algorithms, and the high-quality signal can provide more accurate knowledge for the algorithms to learn. Therefore, the interdiscipline containing soft electronics and machine learning has been widely studied to realize an intelligent system, which can not only detect the physiological signal but also diagnose it. Hence, the soft electronics and machine learning algorithms complement each other.

To meet the soft requirement, many structures such as serpentine [10], nanomesh [11, 12], and wavy [13] have been applied. In addition, many nanomaterials have been demonstrated to adapt to the complex interface, such as the two-dimensional (2D) material [14], carbon nanotube (CNT) [15], nanowire, and organic materials. These materials have been widely applied in soft solar cell [5], light emitting diode (LED) [16], sensors [15, 17], transistors [18], etc. Thus, the soft material will be discussed in the second section, which is the fundamental of soft electronics.

Among the applications of soft electronics, the health monitoring is an important function [8, 19]. Human body is full of physiological signals, which can reflect the conditions of ourselves. Some signals have been widely used in the diagnosis and prevention of diseases not only in hospital, but also in our daily life. For example, the pulse wave has been widely used in the diagnosis in the traditional Chinese medicine for 1000 years [20]. The respiration is an important parameter in health monitoring especially in the respiratory disease [21], particularly for the COVID (2019) [22]. Intraocular pressure (IOP) is the prime indicator for the diagnosis and treatment of glaucoma [23]. Electrocardiogram (ECG) is an important basis for judging cardiovascular diseases [24]. Electroencephalogram (EEG) can be used to diagnose epilepsy and mental diseases [25]. However, the transitory physical examination may have large uncertainty. The result may depend on the testing time and location. It is very meaningful to monitor the physiological signals whenever and wherever, which requires the good sensitivity, flexibility, and comfort of devices. Besides, the physiological signals provide the data for the algorithms to learn, which is the prerequisite of the machine learning. Therefore, the physiological signals and related soft monitoring devices will be discussed in the third section.

With the tight contact between the soft electronics and skin, the continuous and real-time physiological signal monitoring can be realized. Some sudden signals unable to be captured in hospital can be detected, which is important to the diagnosis. In addition, the good interface between skin and soft devices can further improve the SNR. The quantity and quality of physiological signal database can be optimized based on the soft electronics, which can improve the performance of machine learning algorithm, conversely. Before the extensive research of neural network algorithm, many classic algorithms have been used to classify the signals detected by the soft devices. Therefore, machine learning algorithm such as principal component analysis (PCA), linear discriminant analysis (LDA), Gaussian naive Bayes (GNB), support vector machine (SVM), *k*-nearest neighbor (kNN), K-means, decision tree (DT), etc., will be discussed combining with the soft electronics. Recently, due to the easy building and powerful characteristic, neural network has been investigated extensively. Hence, neural network algorithms including fully connected neural network (FNN), convolutional neural network (CNN), recurrent neural network (RNN), and spiking neural network (SNN) will be reviewed in detail in the fourth section.

Although the soft-electronics concept has been proposed for many years, the commercialization of soft devices has not been developed as fast as the research. The most notable application may be the soft screen, which doesn’t need the close contact with human body continuously. This can be attributed to many problems. First, compared with the silicon process, the production and fabrication of soft device are not stable and mature as the silicon device, which limits the mass production. In addition, the high price of the nanomaterials is also the obstruction of the commercialization. Secondly, most of the soft devices are fabricated on the dense polymer substrate such as polyethylene terephthalate (PET), polyimide (PI), polydimethylsiloxane (PDMS). With the interface mismatching to skin and low gas permeability, the wearing experience will be largely influenced. Therefore, the material is the key point of the high-comfort soft devices. Thirdly, the human body is a complex system with many kinds of physiological signals, each specific signal has its own characteristic. The soft electronics should be designed according to the signal. Fourthly, most of the research is still in the single device level, it is urged to realize the total soft system containing sensor, circuit, and intelligent algorithm, whose hardware parts all have the tight contact with skin. Finally, the signals obtained by the soft devices have the advantage of real-time, consecutive, and long-term. Large-scale datasets can be easy to build. Therefore, some datasets and algorithms for the soft devices need to be built and studied to realize the intelligent system, which can diagnose the physiological signals autonomously. According to the problems above, the challenge and outlook of soft electronics assisted by machine learning algorithm will be discussed in the fifth section.

**Fig. 1:**
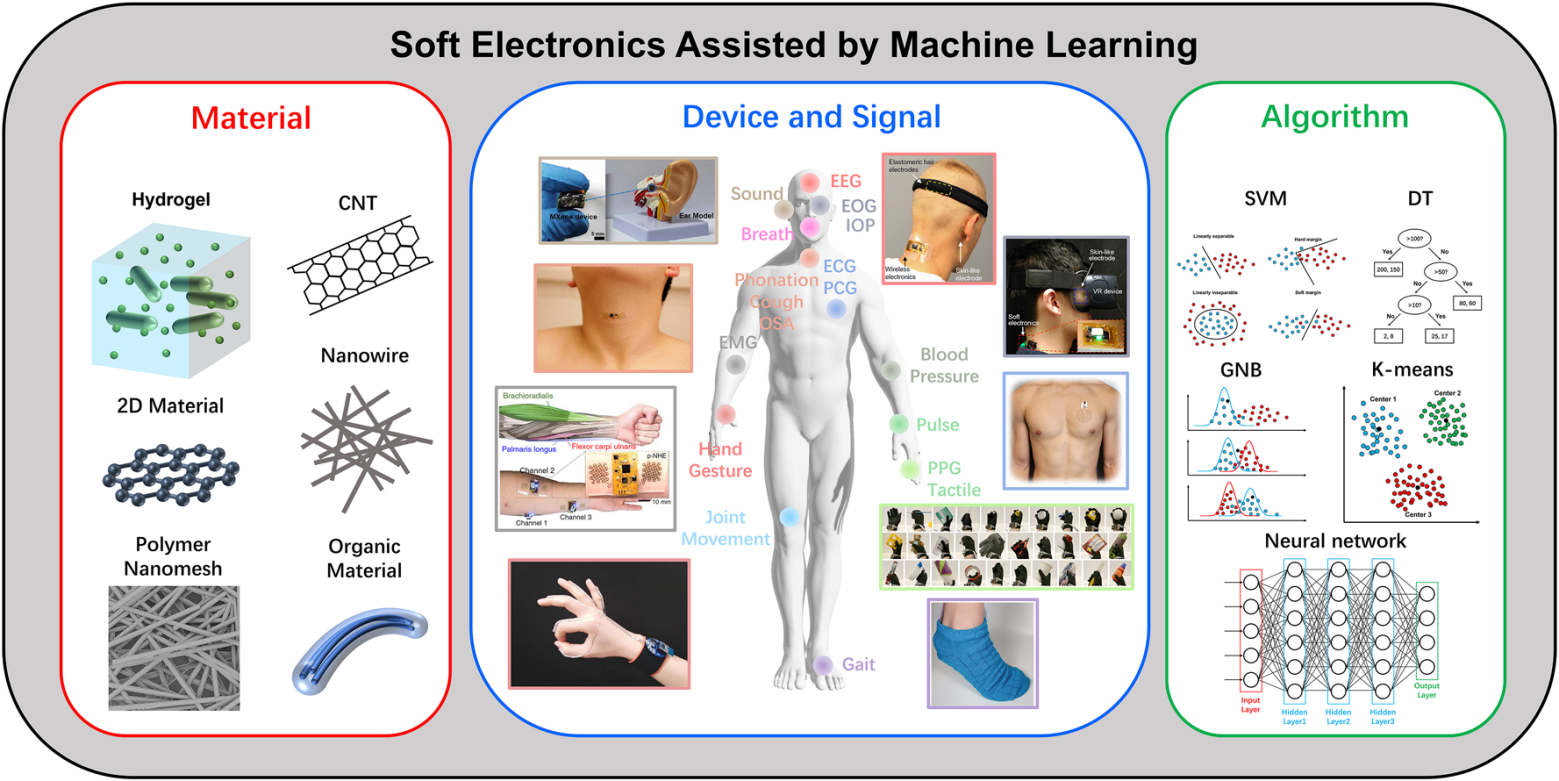
Many new soft materials (2D material, CNT, nanowires, polymer nanomesh, hydrogel, etc.) have been applied to monitor physiological signals, such as EEG, EOG, IOP, breath, ECG, joint movement, blood pressure, pulse, photoplethysmography (PPG), EMG, and gait. The soft physiological monitoring system can be more and more intelligent assisted by algorithms such as SVM, DT, GNB, K-means, and neural network. Reproduced with permission [26–29]. Copyright (2016), (2020), (2022), American Association for the Advancement of Science. Reproduced with permission [30–33]. Copyright (2019), (2020), (2021), Nature Publishing Group. Reproduced with permission [34]. Copyright (2020), Wiley–VCH

In all, the soft electronics with good interface with skin can monitor the physiological signals in real time. Therefore, some sudden diseases can be alerted timely and the database of specific user can be built and enlarged. By combining with the machine learning algorithms, the soft system can not only detect the physiological signals but also diagnose them, which can reduce the burden on doctors. For example, during the popularity of COVID-19, a large number of inquiries increased the workload and the risk of infection of doctors. In a word, the intelligent soft electronic will lead to a healthier life, which is much meaningful to the human society. To realize the soft electronics, the materials and structures of the device should be designed. In addition, due to the variety of physiological signals, the structure of specific device should also be optimized. For easy reading, before talking about soft electronics assisted by the machine learning algorithms, soft materials and physiological signals will be discussed. In this review, we will first give an introduction to the soft material, physiological signals, and machine learning algorithms as well as their relationship. Then, some soft nanomaterials will be reviewed, respectively. After that, soft electronics based on the nanomaterials for physiologic signals monitoring will be discussed according to the signal types. The intelligent soft electronics assisted by machine learning algorithms will be reviewed. Finally, the challenge and outlook about the intelligent soft electronics will be given (Fig. 1).

## Soft Nanomaterials

### CNT

The extraordinary electrical and mechanical properties of CNT make it ideally suitable for soft electronics, especially high-performance wearable sensors, soft display, thin film transistors (TFTs), the implementation of complementary metal–oxide–semiconductor (CMOS) circuits, and the realization of medium-to-large-scale integrated circuits (ICs) and monolithic three-dimensional (3D) integration [35]. As one of promising candidates for next-generation electronic materials, CNT exhibits excellent properties for constructing high-performance soft electronics, including great mechanical flexibility [36], high carrier mobility [37], high current-carrying capacities [38], ultrathin body for effective electrostatic control, and the solution-processability for low-cost production [39]. Although there is still large room to improve the purity and density of the CNT for better performance, the currently available CNTs are adequate for the application in soft electronics with the large critical device dimensions. Many explorations have been carried out using CNT, and tremendous developments have revealed the superiority of the implementation of CNT in soft electronics [40, 41].

**Fig. 2:**
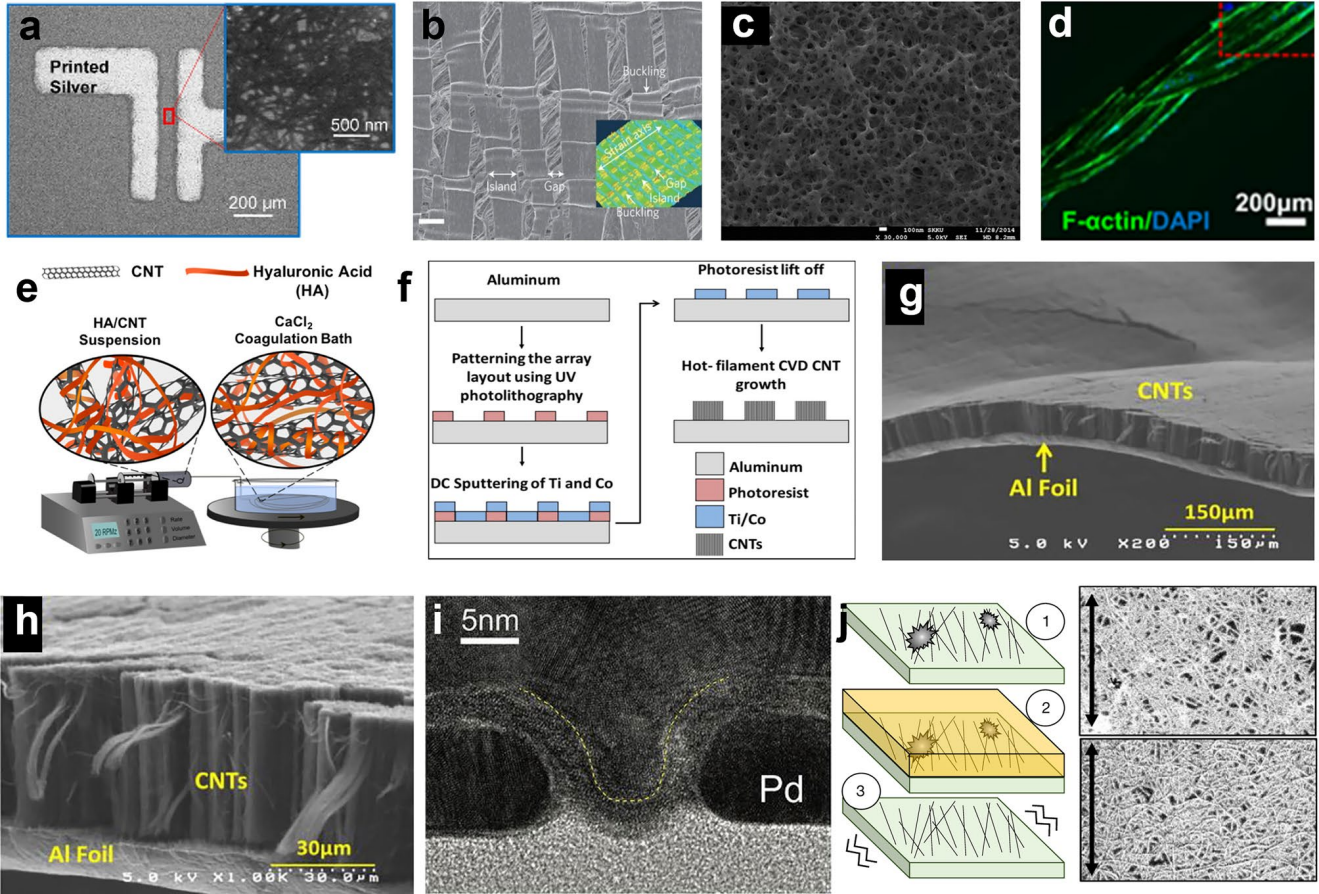
Microstructure and fabrication process of CNTs-based devices. **a** SEM image shows the SWCNT network between the printed Ag electrodes. The inset shows the incubated SWCNT network on the PET substrate as the channel region. Reproduced with permission [42]. Copyright (2016), American Chemical Society. **b** SEM image of the fractural structure of the SWCNT film grown from patterned catalysts using water-assisted CVD. Scale bar represents 5 µm. Inset: 3D image at 100% strain. Reproduced with permission [44]. Copyright (2011), Nature Publishing Group. **c** Top-view FE-SEM image of the PU-Poly(3,4-ethylenedioxythiophene)/Poly(styrenesulfonate) (PEDOT:PSS)-PDMS hybrid structure with SWCNT solution drop coated on it. Reproduced with permission [45]. Copyright (2015), American Chemical Society. **d** Implantable CNTs-based hybrid microfiber with tissues on it and **e** Schematic illustration of the wet-spinning setup for the fabrication of SWCNT hybrid microfibers with SWCNT concentration of 4 mg mL^−1^. Reproduced with permission [47]. Copyright (2019), American Chemical Society. **f** Fabrication process of the 3D patterned CNT array on aluminum substrate, and SEM micrographs of the vertically aligned CNTs on the aluminum substrate at different magnifications: **g** 200 × ; **h** 1 k × . Reproduced with permission [52]. Copyright (2017), Elsevier. **i** TEM image of a normal Pd-contacted CNT FET with gate length of 5 nm, the CNT FET synthesized by CVD. Reproduced with permission [65]. Copyright (2017), American Association for the Advancement of Science. **j** Removal of the aggregates CNTs on the wafer and the SEM image of it (the top-view CNT incubation pre-removed, and the bottom shows CNTs left on the wafer post-removed). Reproduced with permission [66]. Copyright (2019), Nature Publishing Group

CNT has great potentials in soft displays (Fig. 2a) [42], wearable health (Fig. 2b) [43], sport monitors (Fig. 2c, d) [44–46], implantable medical devices (Fig. 2e) [47], etc. Electronic skin (e-skin) as a representative soft integrated sensor system usually consists of variable sensors on a soft platform that can spatially map or quantify certain stimuli, such as pressure [48], temperature [37], electromyograms (EMG) signals [49], ECG signals [50]. These platforms have drawn great attention for potential applications in wearable electronics, robotics, health monitoring, and medical prostheses. By closely integrating interface circuits with sensors, the SNR can be greatly enhanced by the in situ signal processing capability. For this purpose, the interface of circuits should also be mechanically soft with appropriate performance.

CNTs are usually produced as a mixture of semiconducting and metallic nanotubes. Since only semiconducting nanotubes can be applied as the channel of transistors, the metallic nanotubes are typically not utilized, though it can be used as resistive load devices [51]. The purity of CNTs mainly depends on the preparing strategies, which will be discussed later.

Several strategies are currently available to prepare CNT networks and thin films, which can generally be classified in two categories: dry processes and solution processes. Dry processes are mainly chemical vapor deposition (CVD) and dry drawing from vertically aligned CNT arrays [52, 53]. As shown in Fig. 2f–h, CVD-grown single-walled CNT (SWCNT) films comprise ultralong nanotubes bonded by strong connections and thereby possess excellent conductivity, making them suitable for the electrode materials of many functional devices like super-fast actuators [54], stretchable supercapacitors [55], and strain sensors [56]. As for the solution-based process, where several methods have been reported including vacuum filtration [57], rod coating, drop coating, and printing [58, 59]. The solution process of CNTs can be achieved by successfully dissolving them in suitable organic solvents or in aqueous solution with the assistance of certain type of surfactants [36]. One disadvantage of CVD-grown SWCNT is purity. About one-third of the grown SWCNTs are metallic and two-thirds semiconducting SWCNTs (s-SWCNTs). Metallic-SWCNTs (m-SWCNTs) generally increase the current density of the film due to the higher current-carrying capacity, and can be used as the electrodes in various devices. However, the percolation path of m-SWCNTs connecting source to drain electrodes will lead to the short-circuit of the field effect transistor (FET) and the decreasing of the on/off ratio [60, 61]. The presence of m-SWCNTs also limits the channel length of the FET because shorter channels increase the probability of generating a percolating path of m-SWCNTs between the source/drain contacts. One of the common methods to remove m-SWCNTs in thin films of random networks or aligned SWCNTs is the selective electrical burning of m-SWCNTs [62]. Another method, particularly for random network of SWCNTs, is the strip method [63]. The SWCNT film is simply fabricated into narrow strips using conventional lithography and reactive-ion etching. Other than the methods mentioned above, selective plasma/gas etching, light irradiation, chemical surface reaction, and selective chiral growth have also been developed to selectively remove m-SWCNTs. These techniques have exhibited some success by fully or partially removing m-SWCNTs.

s-SWCNTs typically exhibit unipolar* p*-type behavior, which has been attributed to the doping of SWCNTs by oxygen in air or oxidizing acids during solution processing. To enable various applications such as diodes and complementary logic circuits, it is important to be able to fabricate* n*-type SWCNT transistors. Many techniques have been used to convert SWCNT FETs from* p*-type to* n*-type. One way is to change the contact metals from high work function metals to low work function metals, such as Al, Ca, and Sc, which aligns the metal Fermi level closer to the conductance band and reduces the barrier for electrons at the contacts [51]. Other techniques include electrostatic doping, annealing in hydrogen or in vacuum, passivation with inorganic oxides, and chemical doping with potassium, polyethyleneimine (PEI), hydrazine, polymer, electrolyte, viologen, and nicotinamide adenine dinucleotide [64].

ICs, being the core unit of electronic systems for information processing, are required to have decent electrical performance and mechanical flexibility and the ability to be integrated with other components. CMOS technology is the fundament of modern ICs and is also essential to pushing CNT-based soft electronics toward the next stage. Qiu et al. realized a 5 nm CNT FETs approached the quantum limit of FETs by using one electron per switching operation (Fig. 2i) [65]. Hills et al. have fabricated a 16-bit microprocessor based on the RISC-V instruction set, comprises more than 14,000 complementary metal–oxide–semiconductor CNT FETs (Fig. 2j) and is designed and fabricated using industry-standard design flows and process [66]. These works experimentally validate a promising path toward practical beyond-silicon electronic systems.

CNTs have been proven to be the material for high-performance soft electronics owing to the intrinsically great electric/mechanical properties and the low-temperature fabrication processes. The performance of CNT TFTs is much better than those using organic materials and metal oxide semiconductors, and surpassing those of silicon-based devices with similar channel lengths. In addition, CNTs are solution-processable, which can be deposited onto a large area of glass and soft substrates in a suspension at low temperature and cost. In one of the demonstrated works, integrating screen-printed active matrix CNT-based TFTs and electrochromic pixels showed a very good example of a cost-effective platform for large size soft displays [67].

### Graphene

Graphene was discovered by Andre Geim and Konstantin Novoselov, who brought the monolayer graphene from the previous scientific hypothesis to the reality [68]. From then on, especially during the last decade, graphene has showed its revolutionary application potentials in wearable electronics and materials field, due to its excellent characteristics such as high electron mobility (350,000 cm^2^ V^−1^ s^−1^) [69], Young’s modulus (1 TPa) [70], thermal conductivity (5300 W m^−1^ K^−1^) [71], large specific surface area (2600 m^2^ g^−1^), and limited thickness (0.34 nm) [68].

**Fig. 3:**
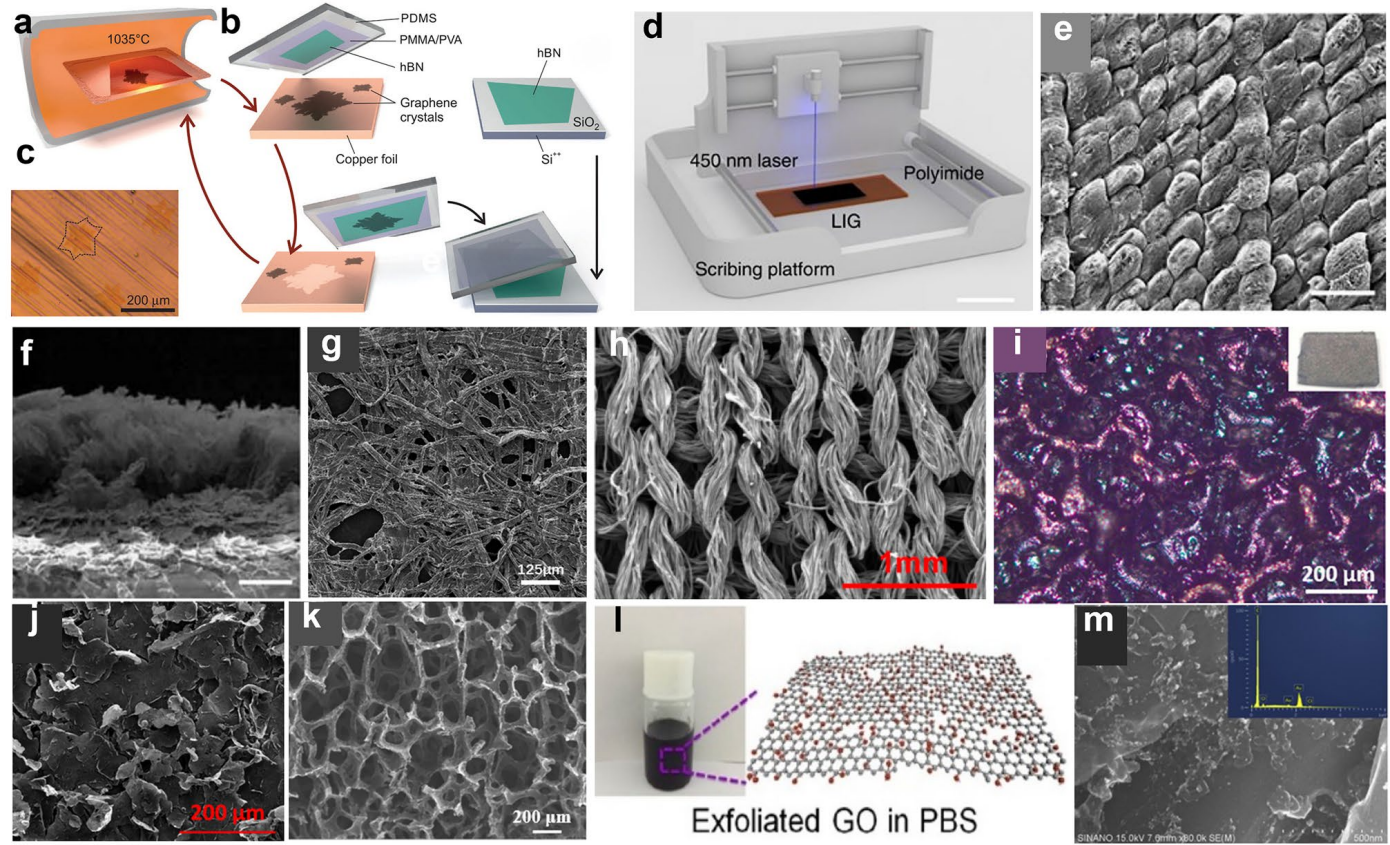
Microstructure and fabrication process of graphene-based devices. **a** Illustration of the CVD furnace with a Cu enclosure inside. **b** Process schematic of the contamination-free transfer of CVD graphene from Cu onto hBN. **c** Optical microscopy image of grown graphene crystals on Cu foil. Reproduced with permission [69]. Copyright (2015), American Association for the Advancement of Science. **d** Schematic illustration of the fabrication process of LSG, and **e** the morphology of LIG sample produced at 290 mW under SEM. Scale bar represents 150 μm. **f** Cross-sectional view of LIG sample produced at 290 mW. Scale bar represents 12.5 μm. Reproduced with permission [101]. Copyright (2017), Nature Publishing Group. **g** SEM images of the tissue paper with rGO. Reproduced with permission [102]. Copyright (2017), American Chemical Society. **h** SEM image of the graphene textile. Reproduced with permission [103]. Copyright (2018), American Chemical Society. **i** Bioinspired graphene pressure sensor with a random distribution spinosum microstructure. Reproduced with permission [104]. Copyright (2018), American Chemical Society. **j** Self-overlapping graphene sheets and stacked structure with numerous interlayer gaps. Reproduced with permission [105]. Copyright (2018), American Chemical Society. **k** SEM images of porous graphene network. Reproduced with permission [21]. Copyright (2018), Elsevier. **l** Schematic illustration of the GO in PBS, and **m** Typical SEM images of rGO/Au nanoparticles (AuNPs) composite (Insert is the corresponding Energy dispersive X-ray spectrometry (EDS)) which can be used for electrocatalytic oxidation of nitrite at the electrode surface. Reproduced with permission [92]. Copyright (2018), Elsevier

Graphene is commonly referred as a 2D atomically thin sheet made of carbon atoms with a honeycomb lattice, densely packed by *sp*^2^ carbon atoms and can be rolled up to form zerodimensional (0D) fullerene and one dimensional (1D) CNT. Each carbon atom in the lattice has a *π* orbital that contributes to a delocalized network of electronics [72] and has three C–C bonds instead of four bonds like the diamond. These structures are the fundamental of the physical properties shown above. During the last decade, top-down and bottom-up methods have been developed to prepare graphene. The former is mainly based on bulk graphite [73]. External force could be used to peel out graphene, and this category of process can be divided into physical exfoliation and chemical exfoliation. Among them, physical exfoliation mainly refers to the exfoliation using a tape [68]. Meanwhile, chemical exfoliation including intercalation peeling [74], ultrasonic exfoliation [75], electrochemical exfoliation [76], and redox exfoliation [77]. The bottom-up method contains a series of complex reaction processes of carbon-containing precursor, such as CVD [69, 78], and chemical synthesis [79]. Among these methods, three classical methods are usually used: mechanical exfoliation, reduction of graphene oxide (GO), CVD (Fig. 3a–c) [69], etc. Meanwhile, 3D graphene films built by the 2D graphene flakes has potential in gas sensors and sound sources. The choice of graphene morphology should consider many factors, such as application, cost, and process [14]. Recently, the laser-scribed graphene (LSG) and laser-induced graphene (LIG) have drown much attention due to its low cost and fast fabrication of graphene (Fig. 3d). As shown in Fig. 3e, f, the LIG shows porous morphology on soft films, suitable for wearable application. LIG can also be prepared based on many different substrates, greatly enriched the raw materials to produce graphene [80].

Graphene can be fabricated into various forms (Fig. 3g–k). Based on the unique characteristics of graphene, more and more devices have been demonstrated. The high electron mobility and conical bandgap structure are suitable for high-performance photodetectors [81], and TFTs [82]. The ultrasmall thickness allows the bandgap of graphene to be easily tuned by applying a voltage. Therefore, spectrum-tunable LED [83] and window-tunable resistive random-access memory (RRAM) and synapses have been developed. Moreover, the high thermal conductivity is ideal for applications such as the heater [84], actuator [85], and thermoacoustic sources [86].

Among these applications, wearable graphene sensors applied for physiological signals monitoring show great potential. Physiological signals are highly complex, which are influenced by various factors of anatomical, psychological, physiological, social, environmental effects, etc. Wearable sensors should avoid rigid substrate, and have flexibility, biocompatibility, simple fabrication process, low cost. However, there are still many problems related to physiological sensors, the incompact interface and large impedance between human body and sensors will decrease the signal quality. Many physiological signals have been detected using graphene sensors such as mechanical signals like pulse [87], respiration [78], and human motions [88, 89], IOP [90], electrophysiological signals like ECG, EEG, EMG, and electrooculography (EOG) [88, 91]; electrochemical signals like ion and glucose concentration in fluids [92]. With the ultrahigh specific surface area, graphene is a suitable carrier (Fig. 3l, m) that can be modified as a chemical sensor to detect fluid [92] and gas [21].

Up to now, the 2D material has been developed into a system consisting of conductor (graphene [93] and MXene [94]), semiconductor (MoS_2_ [95] and other transition metal dichalcogenides (TMD) [96] and black phosphorus [97]), and insulator (hexagonal boron nitride [98]). The 2D system has potential in the soft electronics [99, 100].

### MXene

In 2011, a new family of 2D carbides, carbonitrides, and nitrides labeled MXene was discovered. Their formula of MXene can be M_1.33_XT_*z*_ or M_*n*+1_X_*n*_T_*z*_ (*n* = 1, 2 or 3), where M is an early transition metal, X is C and/or N and T represents various possible terminations (mainly hydroxyl, -OH, oxygen, -O and/or fluorine, -F) [106]. All known MAX phases (the abbreviation of M_*n*+1_A*X*_*n*_ phases, A is mainly a group IIIA or IVA element) are a group of layered hexagonal materials with P63/mmc symmetry, where the M layers are nearly closed packed, and the *X* atoms fill the octahedral sites. The M_*n*+1_*X*_*n*_ layers are, in turn, interleaved with layers of A atoms. In other words, the MAX phase structure can be described as 2D layers of early transition metal carbides and /or nitrides ‘glued’ together with an A element [107]. The strong M–X bond has a mixed covalent/metallic/ionic character, whereas the M–A bond is metallic. Therefore, in contrast to other layered materials, such as graphite and transition metal chalcogenides, where weak van der Waals interactions hold the structure together, the bonds between the layers in the MAX phases are too strong to be broken by shear or any similar mechanical means. However, by taking advantage of the differences in character and relative strengths of the M–A compared with the M–X bonds, the A layers can be selectively etched by chemical method without disrupting the M–X bonds [108].

Due to the M–A bonds are weaker than the M–X bonds, MXene synthesis can be achieved by selective etching of the A element layers from the MAX phases at room temperature. The vast majority of MXene are obtained by etching the A layer from layered ternary MAX phases and their 2D nature [109], using concentrated hydrofluoric acid (HF) or a solution of lithium fluoride and HF.

Depending on the synthetic methods, the lattice parameter *c* (a parameter which indicates the interplanar spacing of MXene) of MXenes is different [109]. By using this parameter, the hydrated cations enter the space of MXene layers. For example, the lattice parameter* c* of Ti_3_C_2_ synthetic by etching Ti_3_AlC_2_ with 50% HF is 20.3 Å [110], but when etching with 40% HF, the lattice parameter *c* is about 20 Å [111], and the V_2_CT_*x*_ with 50% HF is 23.96 Å [112]. In general, there are two methods of synthesizing MXene. The first is a bottom-up approach, such as CVD, which can produce high-quality films on various substrates. However, this approach is not generally used to fabricate MXene, because the films obtained are not single layer, but few-layer thin films [113]. The second approach is a top-down approach, involving the exfoliation of layered bulk. This approach can be further divided into mechanical and chemical exfoliation. The way to separate the graphene layers by adhesive tape is unsuitable for the MAX phases, because in contrast to most other 3D solids used as precursors to their 2D counterparts, the bonds between the M elements and A are strong covalent/metallic for the most part. Therefore, neither mechanical nor classical chemical exfoliation is possible. The first selectively etching the A layers is required. Recently, approaches to synthesizing MXene by top-down approaches including etching the MAX precursors for multilayers [114], and exfoliation for MXene [115] have been realized. Currently, about 30 different MXene compositions have been synthesized by top-down approaches. More compositions have been predicted by theoretical studies with stability.

**Fig. 4:**
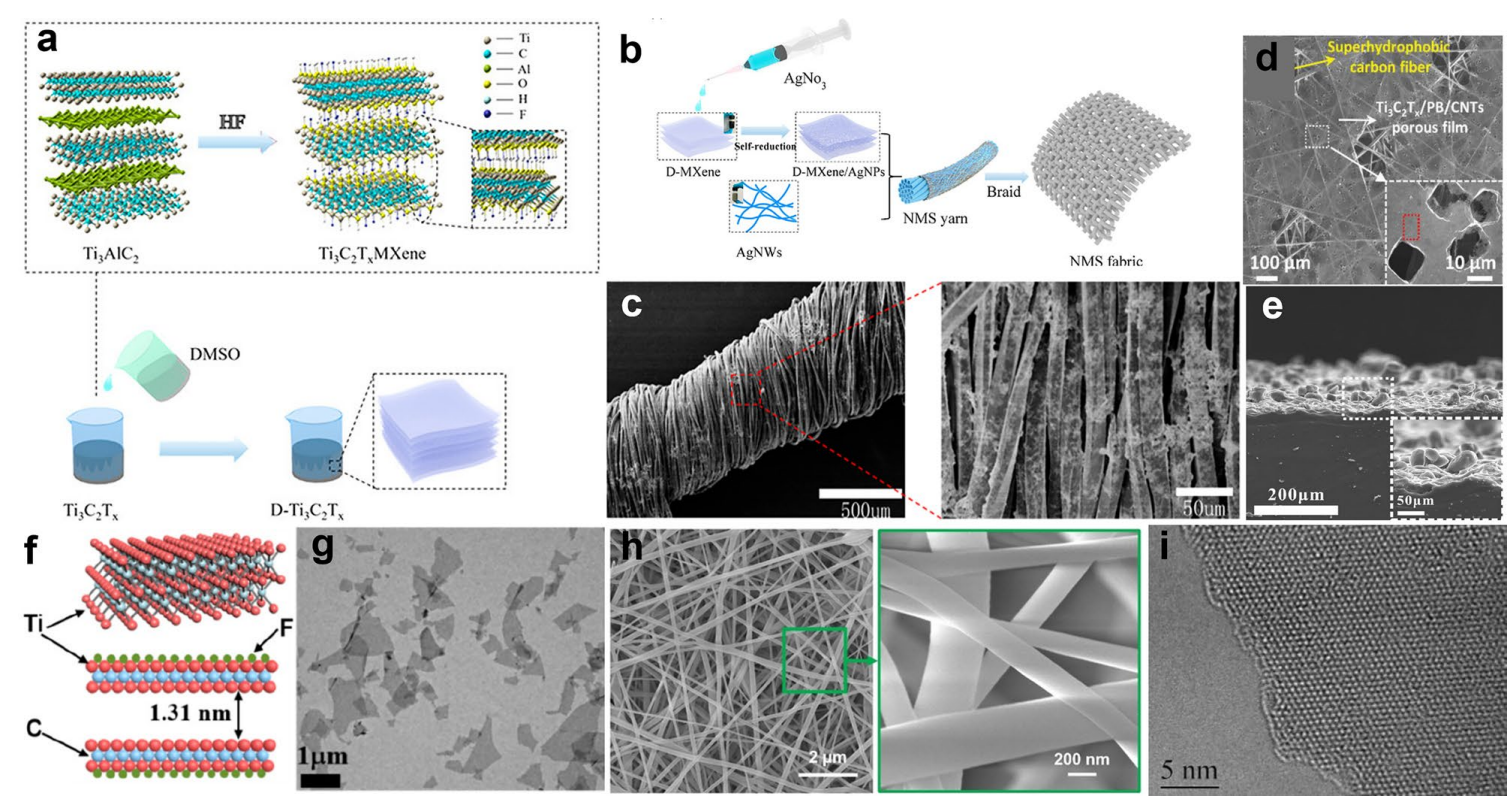
The structure and fabrication process of MXene. **a** Scheme of HF etching Al directly, by adding proportion of the DMSO solution, the MXene nanoblocks were delaminated into nanosheets. **b** AgNPs reduced by Ti_3_C_2_T_*x*_ mixed with AgNWs dipped into the surface of wrapped yarn modified by PDA, and **c** SEM image of yarns coated with MXene. Reproduced with permission [122]. Copyright (2019), American Chemical Society. **d** SEM image of porous and ultrathin Ti_3_C_2_T_*x*_/PB and CNTs ternary film, with the inset (white box) displaying a zoomed‐in SEM image of the holes in the film. Reproduced with permission [123]. Copyright (2019), Wiley–VCH. **e** Schematic illustration of the Ti_3_C_2_ crystal structure and **f** TEM image of MXene nanoflakes, which has thermoacoustic effect for soft MXene earphone. Reproduced with permission [124]. Copyright (2019), American Chemical Society. **g** SEM images showing the rough surface and side of the randomly distributed microspinous MXene-based PDMS obtained using abrasive paper. Reproduced with permission [120]. Copyright (2020), American Chemical Society. **h** SEM image of the MXene composite nanofibrous scaffolds for wearable pressure sensor and the inset showing the morphology at a higher magnification. Reproduced with permission [125]. Copyright (2020), American Chemical Society. **i** Atomic resolution TEM image of a suspended Ti_3_C_2_T_*x*_ nanosheet from top view [121]. Copyright (2020), Nature Publishing Group

For the combination of good properties and easy processing, MXene has various application potential, such as energy storage [116], electromagnetic shielding [117], electrodes [111, 118], electrocatalysis [119], and biosensors [120, 121]. In addition, MXene is easy to be combined with other nanomaterials as a nanosubstrate, which can greatly improve the malleability. When combined with 0D silver nanoparticles (AgNPs) and 1D silver nanowires (AgNWs) (Fig. 4a–c) [122], the elasticity and conductivity of traditional 1D materials can be improved, which ensures continuity and high gauge factor for soft fabric strain sensors for monitoring human motions. For wearable electrochemical biosensors, a MXene-based biosensor system has been proposed for in vitro perspiration analysis by simultaneously measuring physiochemistry signals (glucose and lactate level) using solid–liquid–air three-phase interface designed electrode [123]. As shown in Fig. 4d, the electrochemical detection platform is based on the Ti_3_C_2_T_*x*_ MXene, which consists of Ti_3_C_2_T_*x*_/Prussian blue (PB) and CNTs porous film. The MXene thin film with low heat capacity and special layered structure is emerging as a promising candidate to build sound source [124]. Based on MXene’s thermoacoustic effect (Fig. 4e, f), the MXene earphone has a higher sound pressure level than that of graphene with the same thickness due to the better heat dissipation performance. After packed into a commercial earphone mold, MXene earphone has excellent performance especially at high frequencies, which is suitable for human audio equipment. Inspired by the human skin, a MXene-based piezoresistive sensor with randomly distributed spinous microstructures is designed (Fig. 4g) [120], and it can effectively promote the contact area of the conductive channels and improve performance. Sudeep et al. reported a facile fabrication of highly sensitive and reliable capacitive pressure sensor for ultralow pressure measurement by sandwiching MXene/PVDF-TrFE composite nanofibrous scaffolds (CNS) as a dielectric layer, as shown in Fig. 4h [125]. The proposed sensor can be used to determine the health condition of patients by monitoring physiological signals. Based on the Ti_3_C_2_T_*x*_ nanosheet, the proposed sensor can be used to determine the health condition of patients by monitoring physiological signals and represents a good candidate for the human–machine interfacing device. Tan et al. reported an optoelectronic spiking afferent nerve with neural coding, perceptual learning, and memorizing capabilities to mimic tactile sensing and processing, based on the Ti_3_C_2_T_*x*_ nanosheet (Fig. 4i). The system can detect the pressure by MXene-based sensors, and convert the pressure information to light pulses, and integrate light pulses using a synaptic photomemristor. With the dimensionality-reduced feature extraction and learning, the system can recognize and memorize handwritten alphabets and words, which provides a promising approach toward e-skin, neurorobotics and human–machine interaction technologies [121].

MXene occupies great potential in soft sensors because of its excellent conductivity, mechanical properties, hydrophilicity, and ease to control the morphology [126, 127]. By fully considering the advantages of MXene and the target requirements of devices, a new sensing system is formed by combining MXene materials with other suitable materials [125, 128], which can maximize the synergistic effect between MXene and other phase materials, and thus obtain a high-performance sensor with high sensitivity and wide response range.

### AgNWs

With increasing demand for electronic and photovoltaic devices, it has become critical to ensure the electrical and mechanoelectric reliability of electrodes. Among various alternative materials for soft electrodes, such as metallic/carbon nanowires or meshes, AgNWs networks are regarded as promising candidate [129, 130]. Due to the high conductivity, high transparency, good thermal, chemical, and mechanical properties, more and more applications based on AgNWs have been discussed [131, 132].

**Fig. 5:**
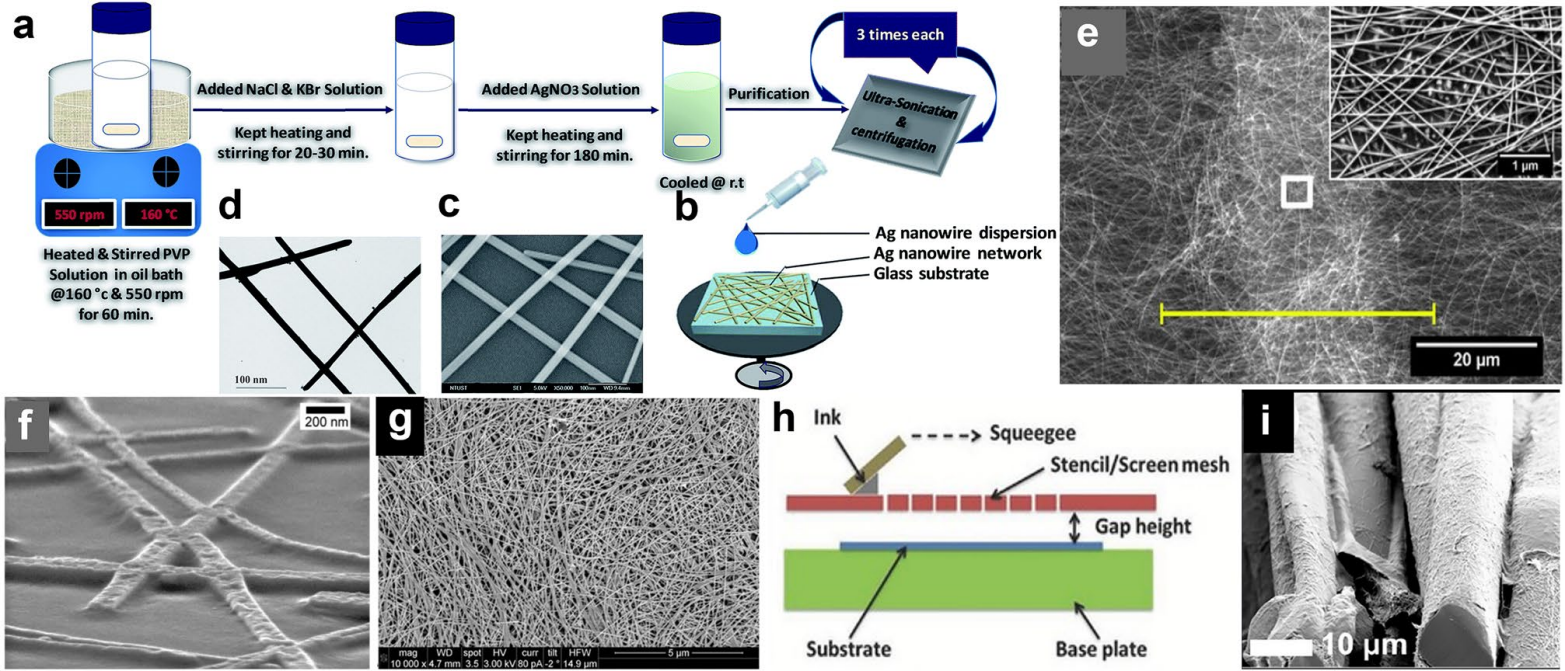
Fabrication process and structures for AgNWs in different fields. **a** Schematic diagram of synthesis and purification of AgNWs with hydrothermal method. **b** Schematic diagram of spin-coated AgNWs network on a glass substrate. **c** SEM image of the spin-coated AgNWs network on a glass substrate. **d** TEM image of the spin-coated AgNWs network on a lacey carbon-coated copper grid. Reproduced with permission [135]. Copyright (2017), Royal Society of Chemistry. **e** Close‐up of fused AgNWs junctions embedded into polymethyl-methacrylate (PMMA). Reproduced with permission [148]. Copyright (2013), Wiley–VCH. **f** SEM image of the interconnection with enlarged interconnection region. Inset showing the contact between AgNWs and ITO. Reproduced with permission [151]. Copyright (2015), Wiley–VCH. **g** Cross-sectional illustration of the screen‐printing process, and **h** SEM image of the dense AgNWs network structure in the screen‐printed AgNWs line for intrinsically stretchable AgNWs TFT array. Reproduced with permission [156]. Copyright (2016), Wiley–VCH. **i** SEM images of individual filaments, which used as a Joule heating element for woven heating fabric. Reproduced with permission [157]. Copyright (2020), American Chemical Society

Up to now, various methods for the synthesis of AgNWs have been proposed which can be originally derived from the metal nanoparticle preparation [133]. At early stages, AgNWs were mainly prepared via electrochemical methods with low yield and non-uniform size. Later, other methods including photochemical reduction [134], hydrothermal methods (Fig. 5a–d) [135, 136], and template techniques [137] were developed. Despite getting considerable progress, it remains a challenge to produce high-aspect ratio AgNWs via a facile and rapid process. More specifically, AgNWs networks are considered as promising alternative transparent conductive electrode materials because of network geometry, no dislocation activity, and high strength [138]. Transparent electrodes (TEs) are crucial for various optoelectronic devices including liquid–crystal displays (LCDs) [139], OLEDs [139], organic solar cells (OSCs) [140], touch screens [141], wearable electronics [142, 143], etc. Their performance highly depends on the fabrication method and the characteristics of AgNWs networks.

Currently, the mainstream of TEs relies on the technique of high vacuum processes [131]. With the low-temperature processes and low cost, many solution coating processes have been studied to produce AgNWs electrodes through simple, reliable, and cost-efficient deposition techniques, such as spray coating, drop casting, spin coating, rod coating, dip coating, vacuum filtration, slot-die coating, and R2R coating [144]. Based on the fabrications and applications, the properties of AgNWs networks strongly depend on the following features: (i) individual nanowire properties, (ii) the interconnection (junctions) between them [132], and finally (iii) network density. Many works have been done to enhance these features, most of them are focused on the post treatment of the AgNWs network, including thermal annealing [145], mechanical pressing [132], light-induced plasmonic nanowelding [146].

Currently, the most efficient and widely used transparent conducting material is indium tin oxide (ITO). However, when compared with AgNWs, it shows less flexibility, and relatively high manufacturing costs [147]. AgNWs are suitable for preparing transparent soft electrode for its high conductivity, transparency, and mechanical flexibility. For soft OLED, high-efficiency white OLEDs fabricated using AgNWs-based composite TEs show almost perfectly Lambertian emission and superior angular color stability, imparted by electrode light scattering (Fig. 5e) [148]. Besides, 1D AgNWs and 2D graphene can be integrated for transparent OLEDs with similar behavior to the commercial ITO-based counterparts [149]. When used in photovoltaic (PV) modulus [144, 150], the AgNWs increased stability of the OSCs, suited for affordable PV modules. E-skin made by AgNWs electrodes (Fig. 5f) enables real-time super-resolution imaging of pressure distribution, which may have large impact on health care and security affairs [151]. Recently, with the rapid growth of soft electronics, carbon nanomaterial-based sensors have shown outstanding performance due to their superior mechanical and electrical properties. Highly sensitive strain sensors have been reported by using graphene sheets on the soft substrates [152]; with 1D structure and high transparency, AgNWs-based devices can gain higher stretchability and optical advantages than traditional carbon-based devices [142, 153]. It is easy to integrated with fibers for clothing-integrated sensors [142, 154]. Kim et al. proposed a soft smart sensor system integrated on soft contact lenses that achieved wireless ocular diagnostics [155], the AgNWs-graphene hybrid material was used to make field effect sensor and antenna, which is suitable for using in eyes. Since AgNWs can disperse homogeneously in water, Liang et al. fabricated a stretchable TFT array by screen-printed AgNWs (Fig. 5g, h), which revealed a low cost way for printed electronics [156]. Clothe can heat themselves spontaneously, based on the conductivity and thermal effect of AgNWs, Hwang et al., realized a soft heaters using AgNWs/PEDOT:PSS composition (Fig. 5i), which is machine-washable [157]. For electromagnetic interference (EMI) shielding, a soft device was demonstrated with AgNWs network on a PDMS substrate. Considering the increase in the EMI shielding effectiveness at low AgNWs density, this unique phenomenon is attributable to the effective shielding of the incoming EV wave [158].

AgNWs assembled into random networks have problems such as rough surface roughness, non-uniform networks, and high nanowires–nanowires junction resistance [141]. Recently, many studies have been carried out on techniques for the alignment of AgNWs, such as external magnetic or electric fields-based assembly [159], flow-enabled technique [160, 161], rod coating technique [141], and capillary printing technique [162]. Jung et al. fabricated a conductive nanomembrane with 540% elongation by float assembly method [163]. This method enabled monolayer compact packing of nanomaterials at the water–oil interface and fabrication of a nanomembrane with a cross-sectional structure in which metal nanomaterials are partially embedded in an ultrathin elastomeric membrane. The teeth-like nanowire structure allows high-resolution patterning of nanowires using photolithography without damaging elongation because nanowires are partially exposed from the elastomer. Moreover, contacts between nanowires can be consolidated further by cold welding of the partially exposed nanowires firming connections across nanowires. The conductivity of monolayer can differ depending on measurement directions, 103,100 S cm^−1^ in the parallel direction or 32,900 S cm^−1^ in the vertical direction. When two nanomembranes are stacked with the nanowires aligned to each other, a maximum conductivity of 165,700 S cm^−1^ was achieved. The stacked nanomembrane remained conductive up to ~ 400 or > 1,000% strain, parallel or vertical to the direction of nanowires, respectively.

AgNWs network offer opportunities for fundamental and applied research. Thanks to the easy fabrication, and the excellent electrical, optical, and thermal properties, AgNWs networks exhibit great potential for applying in various fields [164, 165]. Recently, investigations on enhancing the nanostructure for soft applications are increasing, such as nanomesh, nanofibers, and core-sheath structure to fulfill requirements in soft and implantable application [163, 166, 167]. Also, there are other kinds of metallic or nonmetallic nanowires (copper, gold, core–shell, organic nanofiber, etc.), which are suitable for a large variety of applications, which will not be discussed in this article.

### Hydrogel

**Fig. 6:**
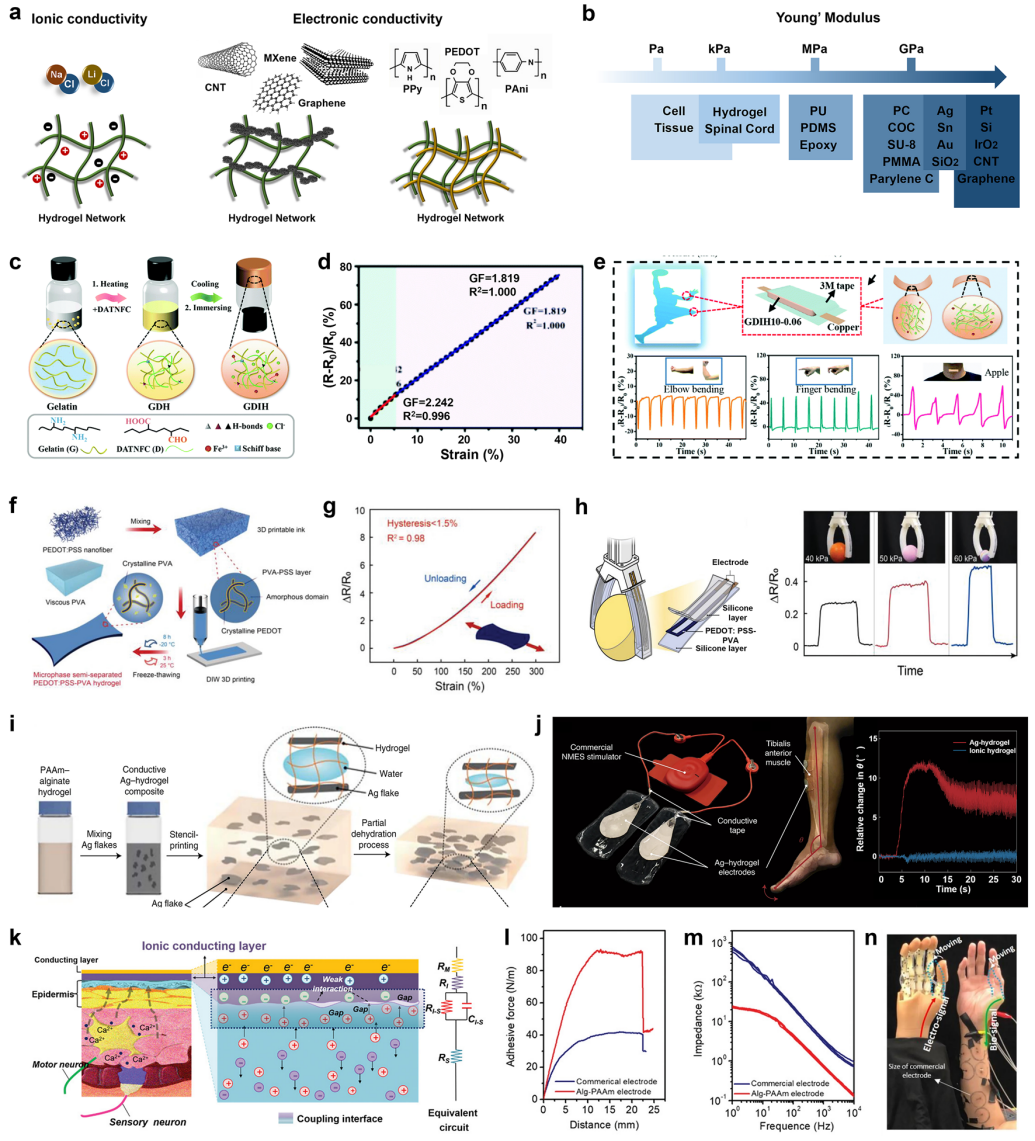
Hydrogel-based soft devices. **a** Material design of ICHs and ECHs. **b** Young's moduli of common electrode materials and cell/tissue. **c** Schematic illustration of the preparation and internal structure of conductive gelatin/nanofibrillated cellulose/Fe^3+^ hydrogels, and the relative resistance changes and GF with the increase of tensile strain (**d**), as well as their applications in monitoring elbow flexion, index finger flexion, throat vibration (**e**). Reproduced with permission [179]. Copyright (2022), Royal Society of Chemistry. **f** Principle and fabrication process of the PEDOT:PSS-PVA conducting polymer hydrogel, and the loading and unloading resistance responses of the PEDOT:PSS-PVA hydrogel strain sensor with a strain of 300% (**g)**, as well as its application as robotic skins for sensory grasping (**h**). Reproduced with permission [178]. Copyright (2022), Wiley–VCH. **i** Schematic of the electrode and skin for sEMG and coupling process of the ionic fluxes in electrolytic tissue media and electronic current in the recording electrode. **j** 90° peel-off test of electrode based on Alg-PAAm gel and commercial gel. **k** Contact impedance verse frequency of the Alg-PAAm electrode and commercial electrode. **l** Needle grasping driven by sEMG signals obtained by as-prepared Alg-PAAm electrodes. Reproduced with permission [181]. Copyright (2020), Wiley–VCH. **m** Composition and synthesis of the conductive hydrogel composite composed of micrometer-scale Ag flakes and PAAm-alginate hydrogel (Ag-hydrogel composite). **n** Neuromuscular electrical stimulation electrodes made of the Ag-hydrogel composite with a commercial electrical muscle stimulator, and the relative changes in dorsiflexion angle as a function of stimulation time. Reproduced with permission [172]. Copyright (2021), Nature Publishing Group

Hydrogel is a kind of extremely hydrophilic 3D polymer network, which can swell rapidly in water and hold a large volume of water without completely dissolving. Hydrogels exhibit many characteristics similar to natural soft tissues, such as good biocompatibility, adjustable physical and chemical properties, and high water content, and have always been one of the most widely used biological materials [168–170]. Furthermore, since the first report in 1994 of introducing conductive components into conventional hydrogels to obtain electroconductive hydrogels (CHs), the multifunctional CHs have been garnering tremendous interests in soft electronics, sensors and actuators, human–computer interfaces, as well as soft energy storage [171, 172]. Generally, CHs are composed of conductive components and soft hydrogel substrates. Considering the different conductive components, CHs can be divided into ionically conductive hydrogels (ICHs) and electronically conductive hydrogels (ECHs) (Fig. 6a) [173, 174]. ICHs are generally prepared by dissolving ionic salts (e.g., NaCl, LiCl) into hydrogels. As for the ECHs, the conductive components mainly include noble metal NPs and nanowires, carbon nanomaterials (CNTs, graphene, etc.) and other novel 2D materials (e.g., MXene), as well as several intrinsically conductive polymers with various ionic dopants, such as polypyrrole (PPy), polyaniline (PAni) and PEDOT [168, 175]. By integrating these conductive nanomaterials into the hydrogel matrix, the composite CHs can possess the ideal electronic conductivity, while retaining the reinforced biomechanical advantages of hydrogels. In addition, a wide range of natural polymers and synthetic polymers have also created infinite possibilities for the design and synthesis of CHs.

Currently, multifunctional CHs have been broadly used in wearable and implantable soft bioelectronics due to their intrinsic skin-like and tissue-like properties [173, 176]. Overall, the advantages of CHs as soft bioelectronics are: (i) the adjustable conductivity over a wide range, (ii) the excellent biocompatibility (antibacterial, etc.), (iii) the ideal flexibility and elasticity, as well as favorable biomechanical interactions with biological tissues, (iv) the available bio adhesive properties at highly conformal electrode–tissue interfaces, even in humid environments, (v) the abundant and wide range of hydrogel materials for the “green” electronics. Briefly, for the design and synthesis of CHs in different applications, it is necessary to take into consideration the conductivity and mechanical properties of CHs, as well as the interaction between CHs and biological tissues. Yunsik et al. embedded Ag flakes into polyacrylamide (PAAm)–alginate hydrogel matrix [172], followed by the partial dehydration process, to obtain an electrically conductive hydrogel with high electrical conductivity (374  S cm^−1^), low Young’s modulus (< 10 kPa) matching several biological tissues, and high stretchability (250% strain). Apart from a variety of conductive fillers, the intrinsic hydrogel substrate materials have endowed the CHs with ideal biomechanical properties and adhesive properties. Conventional electronic materials are much stiffer than biological tissue, which may induce adverse biomechanical interactions at the electrode–tissue interface. Differently, in terms of Young’s modulus, the mechanical properties of CHs are similar to those of skin and other biological tissues, probably minimizing the mechanical mismatch with tissues (Fig. 6b). Besides, CHs with favorable bio adhesive properties are more likely to establish highly conformal and stable bioelectronic interfaces on the biological surfaces, which is beneficial to reduce interfacial impedance and promote bioelectrical signal transmission [177].

CHs-based stress/strain sensors have been widely used in human motion monitoring, prosthetic control, human–computer interaction (HMI), and touch panels [178, 179]. Different from traditional elastomer materials with brittle mechanical properties and insufficient biocompatibility, the comprehensive properties of CHs are expediently adjusted in terms of ionic and electronic conductivity, biocompatibility, antibacterial property, self-adhesion, elasticity, and flexibility, by means of reasonable material and structural design. Therefore, the CHs-based stress/strain sensors will exhibit excellent performance in mechanical stability, high strain sensitivity, wide linear range, fast response and recovery, low hysteresis, and fatigue resistance. As shown in Fig. 6c, Fu et al. constructed environmentally friendly, fully recyclable strain sensors based on self-healing, recyclable and conductive gelatin/nanofibrillated cellulose/Fe^3+^ hydrogels [179]. The multifunctional strain sensor possessed favorable strain sensitivity (Gauge factor (GF) = 2.24 under 6% strain) and compressive sensitivity (Sensitivity = 1.14 kPa^−1^ under 15 kPa) (Fig. 6d), and thus could accurately monitor and discern subtle bodily motions, handwriting, and personal signatures (Fig. 6e). Besides, Shen et al. developed a facile one-step compositing methodology combining PEDOT:PSS nanofibers with poly (vinyl alcohol) (PVA) [178], to create a unique microphase semi-separated network of CHs (Fig. 6f). The as-prepared PEDOT:PSS-PVA hydrogel strain sensor could exhibit high stretchability (300%) and ultralow hysteresis (< 1.5%) (Fig. 6g). The strain sensor with stable performance and high robustness could reliably enable precise, real-time remote control of industrial robots (Fig. 6h).

As a new generation of bioelectronic materials, the CHs have enabled the successful construction of highly conformal electrode–tissue interfaces, for high-quality bioelectronic stimulation and recording [30, 180]. Benefiting from the CHs with ideal stretchability and stiffness matching the biological tissue, the CHs-based patch electrode can closely fit on the uneven biological surface, even under the disturbance of dynamic mechanical deformation [181]. In addition, the CHs with intrinsic self-adhesive properties also ensure the high conformality and long-term stability of the hydrogel electrode–tissue interface. Pan et al. designed and fabricated compliant ionic electrodes based on highly self-adhesive Alg-PAAm/LiCl hydrogels, which were able to enhance the intermolecular interaction with the biological surface and eliminate the microgaps at the electrode–tissue interface (Fig. 6i) [181]. Therefore, the Alg-PAAm compliant electrode exhibited bioadhesive properties far superior to commercial electrodes (Fig. 6j), and had an ultralow interfacial impedance (20 kΩ) with skin (Fig. 6k). As shown in Fig. 6l, this electrode could record dynamically weak sEMG signals with high SNR and low crosstalk, for the successful and precise control of the prosthesis to perform fine and sophisticated motions. Yunsik et al. fabricated neuromuscular electrical stimulation electrodes based on Ag/Alg-PAAm hydrogels with high electrical conductivity and soft conformability (Fig. 5m) [172]. The Ag-hydrogel electrode could deliver high-frequency electrical signals with sufficient current to induce dorsiflexion in the foot, and drive more angular movements of the fingers compared with the normal ionic hydrogel electrodes (Fig. 5n).

### Polymer Nanomesh

**Fig. 7:**
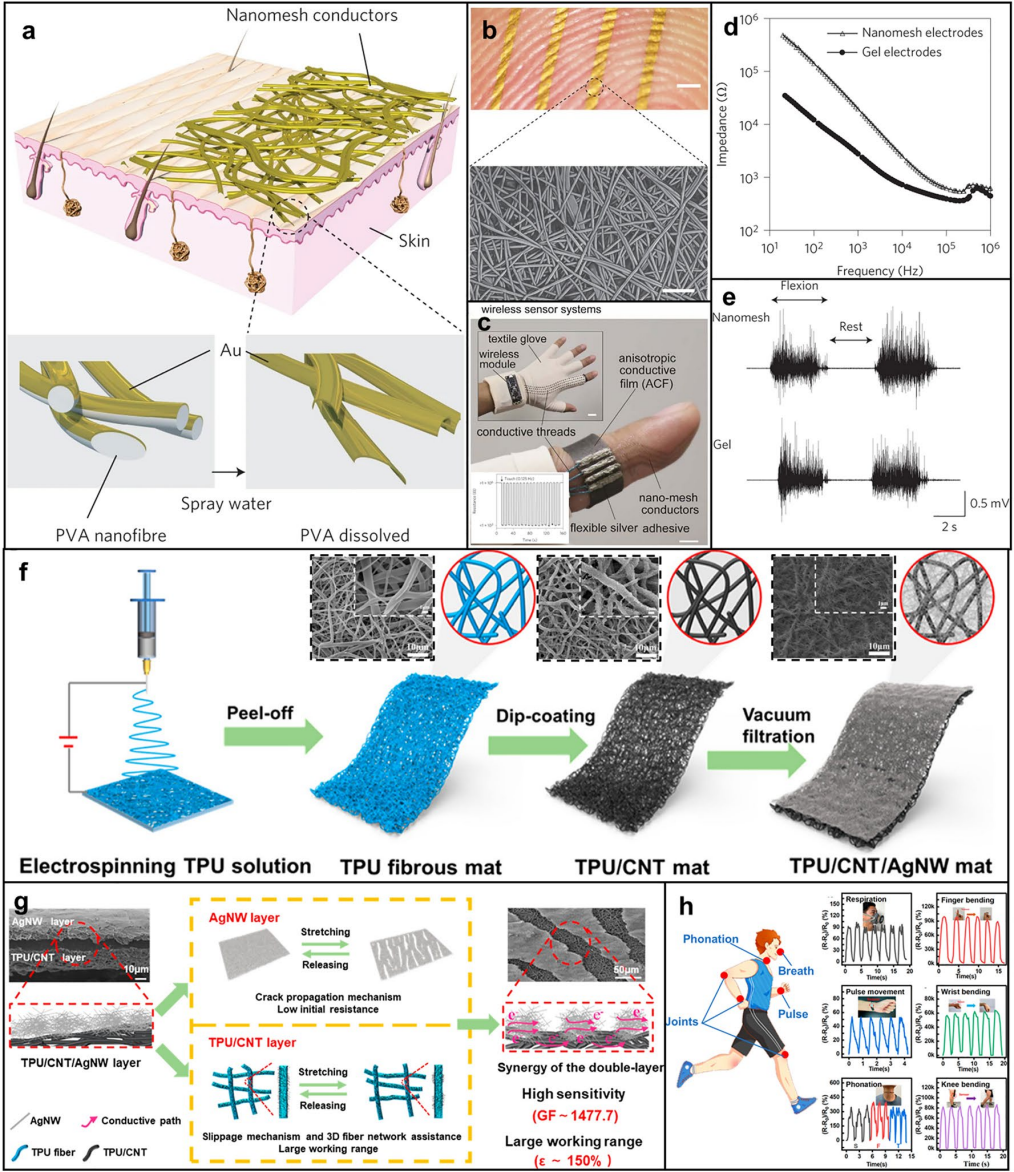
Application of polymer nanomesh in soft sensors. **a** Preparation process of the on-skin nanomesh electronics: first, Au is evaporated onto PVA nanomesh obtained by electrospinning; then, PVA meshes are dissolved by spraying water so that nanomesh conductors can adhere to the skin. **b** Picture of nanomesh conductor attached to the fingertip (Scale bar represents 1 mm) and the SEM image of the nanomesh conductor after dissolving PVA nanomesh (Scale bar represents 5 µm). **c** On-skin wireless sensor system based on the on-skin nanomesh electronics for touch sensing (Scale bar represents 3 mm). **d** Measuring the impedance of the skin/electrode interface by using nanomesh electrodes compared with Ag–AgCl gel electrodes. **e** EMG signals were measured on the forearm, while the wrist was flexed at 90° (two times) and at rest by both nanomesh and gel electrodes. Reproduced with permission [11]. Copyright (2017), Springer Nature. **f** Preparation of the PU/CNT/AgNWs strain sensor, and the SEM images of PU nanomesh, PU/CNT nanomesh and PU/CNT/AgNWs nanomesh. **g** Design concept of the double-layered conductive network for the PU/CNT/AgNWs strain sensor. **h** Applications of the PU/CNT/AgNWs strain sensor for monitoring different motion signals. Reproduced with permission [204]. Copyright (2020), American Chemical Society

Recently, soft electronics have been widely used in the field of health care, playing a great role in monitoring biophysical signals, such as physiological electrical signals (ECG, EMG, etc.) and mechanical signals (pulse, respiration, joint bending, etc.) generated by vascular dynamics and human motions [8]. The monitoring of these signals is of great significance to the prevention and diagnosis of diseases and the recovery and reconstruction of motor function. Among the materials used to prepare sensors, polymer nanomesh with porous structure has shown great potential in the field of soft electronics due to its advantages, such as ultrasmall weight, high water vapor permeability, good skin compatibility, and good stability [11, 182, 183]. For example, Miyamoto et al. prepared an electrode based on the Au/PVA nanomesh as shown in Fig. 7a, which is inflammation-free, gas-permeable, lightweight, and stretchable [11]. After spraying water, PVA nanomesh can be dissolved so that nanomesh conductors can adhere to the skin closely (Fig. 7a, b). This electrode can not only be used as a pressure sensor to realize touch sensing (Fig. 7c), but also to monitor EMG, and the test results are almost the same as those of Ag–AgCl gel electrodes (Fig. 7d, e). The nanomesh can also be used to measure the skin impedance [184, 185]. Wang et al. realized a durable strain sensor based on the Au/PDMS/polyurethane (PU) nanomesh to monitor the facial tissue movements [186].

Nanomesh can be prepared by many methods such as photolithography [187], natural fiber [188], and electrospinning [11]. In this review, the electrospinning polymer nanomesh was mainly discussed. The polymer nanomesh is manufactured by electrospinning technique as the matrix, and then functionalized by other materials to realize the construction of the sensing function. Therefore, the sensors are generally composed of nanomesh and functional modified materials. The former plays a role of structural support, and the latter acts as the signal response element of the sensor.

The polymer materials commonly be used to prepare nanomesh including PU [183, 189–192], PVA [11, 184, 185, 193], styrene butadiene styrene (SBS) [194], styrene ethylene butene styrene block copolymer (SEBS) [195], polyvinylpyrolidone (PVP) [196], etc. Under the electrostatic field, the viscous polymer solution in the syringe forms the Taylor cone at the needle tip due to the combined action of the electric field force, the surface tension and the viscoelastic stress of the solution, and extends into the uniform filament to deposit on the collector. The polymer can be changed into a 2D network film composed of micro- and nano-fibers by electrospinning, which leads to a large specific surface area for the next functional modification.

Functionalized modified materials are generally conductive materials, which can form conductive pathways in nanomesh to respond to external physical stimuli. Commonly used modified materials including CNTs [192, 195], reduced graphene oxide (rGO) [197, 198], MXene [191, 194], metal nanowires or NPs (Au [11, 184–186], Ag [192, 199], Pt [200], etc.). Functional modification methods can generally be divided into two types. One is to add functional materials to the electrospinning solution, forming nanomesh with polymer after electrospinning, and finally complete functional modification through subsequent treatment [196]. The other is to functionalize the surface of polymer nanomesh obtained by electrospinning. Here, due to the simplicity and convenience of preparation, the latter will be discussed. There are many methods of surface modification, the most common one is direct spraying, which is achieved by preparing functional substances into solutions and then coating them on the surface of polymer nanomesh by drop coating [201] or spraying [202, 203]. This modification method can only modify the surface layer of nanomesh. Functional materials can also be modified inside the nanomesh by soaking the nanomesh in the solution supplemented by ultrasound [197], so as to make the modification more solid. The above modification methods are mostly applicable to carbon nanomaterials, MXene, etc. For metal functional modifiers, the commonly used modification materials are generally NPs or nanowires. In addition to modifying the prepared metal nanowires by the above methods [202], the polymer nanomesh can also be soaked in the precursor solution and modified by in situ synthesis of metal NPs on the surface of the nanomesh through chemical reaction [204, 205], or the metal can be deposited on the surface of the nanomesh by sputtering [200]. In addition, in order to improve the stability, strain range, sensitivity, and other properties of the sensor, the nanomesh can be pre-modified before the modification of functional materials to improve the firmness of the modifier. For example, the nanomesh can be pre-modified with polydopamine (PDA) [195, 199] and other functional modifiers [190, 199]. Wang et al. used CNT and AgNWs to modify PU Nanomesh successively as shown in Fig. 7f [190]. The design principle of this sensor is to take advantage of different conductivities of the two conductive layers (Fig. 7g). The high-stretchability PU/CNT substrate layer acts as a structural support, which can realize the integrity of the conductive path even under a large strain. And the AgNWs layer offer a very low initial resistance. The combination of the two gives the strain sensor a wide working range (up to 150%) and a high sensitivity (up to 1477.7); therefore, this sensor can accurately detect the omnidirectional human motions, including subtle and large human motions (Fig. 7h).

### Liquid Metal

Liquid metal and its alloys have become non-negligible materials for soft electronics due to their excellent thermal and electrical conductivity and rheological properties. Due to low vapor pressure, safety, and no pollution, gallium and its eutectic alloys formed with indium and tin are more widely used than highly toxic mercury, such as scalable RF electronics [206], strain sensor [207], thermal elastomer composite [208], microheater [209], epidermal strain sensors [210], electrically self-healing composite [211], and battery for stretchable electronics [212]. Liquid alloys have unique advantages in soft electronics with complex surface structures that require sufficient softness and deformation, including high resolution [213], conformal, stretchability, and self-healing to avoid failure or circuit breaking under cyclic deformation [211]. In an aerobic environment, the liquid alloys surface will form amphoterics solid oxide skin of nanometer thickness [214], which will affect the shape and adhesion of the liquid metal to various surfaces. A variety of technologies for liquid metal patterning have been implemented, such as atomized spraying [215], microchannel injection [216], inkjet printing [217], 3D printing [218], masked deposition [219], and transfer writing [220]. Among them, stencil print technology is undoubtedly the most attractive, because it can achieve economic, fast, mask free, automated, and mass production [221]. However, the high surface tension of liquid alloy and the existence of surface oxide make it difficult to print directly on the flexible substrate, and easy to fracture. Therefore, it is necessary to select a suitable transfer template and modify it, so that it cannot only selectively adsorb liquid metal, but also transfer completely on a variety of substrates [221, 222]. Recently, electron-beam lithography and soft lithography techniques can achieve high resolution at the sub-micron level [213]. In short, how to realize the patterning of liquid alloys in batch, high fidelity, high resolution, and low cost is still a hot research topic.

### Brief Summary

As discussed above, to realize the soft electronics, the kinds and structures of material should be designed. Many novel nanomaterials (such as CNT, graphene, MXene, AgNWs, hydrogel, nanomesh, and liquid metal) have been prepared by well-designed methods (such as CVD, laser inducing, electrospinning, chemical synthesis, and solution-based method), which have great advantage to traditional materials in soft electronics. During the bending and stretching process, the materials must withstand large deformation without damage. Therefore, the breaking elongation of materials should be large enough. For example, the single-layer graphene can be used in the flexible devices. However, due to the fragility of single-layer structure, the materials will be damaged during the stretching process. The structure of materials should also be designed like the porous 3D structure. To some extent, not the thinner the better. In addition, for the large-scale commercial application, how to prepare the soft material in high efficiency and low cost is also crucial. Solution-based methods cooperated with pattern process may be a good choice.

## Wearable Devices for Different Physiological Signals

### Pulse

The pulse is driven by the heart and usually measured at the wrist (radial artery). It is affected by many factors, such as the conditions of blood, and muscle, skin. Therefore, it can reflect some physical conditions of human body. The pulse has been used in traditional Chinese medical science for more than 2000 years [223], where the pulse signals are picked at three acupoints (called ‘chi’, ‘guan’, ‘cun’) [20] with different applied force (small force call ‘fu’, middle force called ‘zhong’, large force called ‘chen’). This pulse condition of three acupoints under three pressure levels constitutes nine indexes.

The pulse rate of human is about 30–200 beats min^−1^. In the frequency domain, the pulse spectrum range is 0–20 Hz, and most of the energy is concentrated within 10 Hz. There are usually 3 peaks in a typical pulse wave (the percussion (P), tidal (T), and dicrotic (D) wave). The relative position and amplitude ratio of these peaks can be used to analyze cardiovascular status [20]. To detect the pulse signal, pressure/strain sensors are widely used, which be divided into three types: piezoresistive [224], piezoelectric [225], and piezocapacitive. Under the pressure, the resistance, voltage, and capacitance would be changed based on these three effects, respectively. Some typical pulse sensors are listed in Table 1.

**Table 1:** Typical soft pulse sensor

Physiological signal	Device type	Active material	Signal type	Mechanism	Detecting source	References
Pulse	Pressure sensor	PZT	Voltage	Piezoelectric	Wrist and carotid artery	[229]
Pulse	Pressure sensor	Nylon netting	Capacitance	Piezocapacitive	Wrist	[230]
Pulse	Pressure sensor	SiO_2_ NP/PDMS	Capacitance	Piezocapacitive	Artificial blood vessel	[231]
Pulse	Pressure sensor	POMaC/PLLA/PHB/PHV	Capacitance	Piezocapacitive	Artery	[228]
Pulse	Pressure sensor	Group III-nitride materials	Voltage	Piezoelectric	Carotid, temporal, brachial, facial, radial, fingertip, femoral, popliteal, posterior tibial, dorsalis pedis	[227]
Pulse	Pressure sensor	Ionic polymer metal composite	Voltage	Piezoelectric	Wrist (Chi, Guan, Cun)	[232]
Pulse	Strain sensor	Graphene	Resistance	Piezoresistive	Wrist	[233]
Pulse	Strain sensor	Nickel-Chrome	Resistance	Piezoresistive	Wrist	[234]
Pulse	Strain sensor	Graphene foam	Resistance	Piezoresistive	Wrist	[235]
Pulse	Strain and pressure sensor	Graphene foam	Resistance	Piezoresistive	Neck artery, Wrist artery, and Fingertip	[236]
Pulse	Pressure sensor	Graphene	Resistance	Piezoresistive	Wrist	[104]
Pulse	Strain sensor	Graphene	Resistance	Piezoresistive	Neck artery, Wrist artery, and Fingertip	[103]
Pulse	Pressure sensor	Graphene	Resistance	Piezoresistive	Carotid artery, Brachial artery, Radial artery, and Dorsalis pedis artery	[226]

**Fig. 8:**
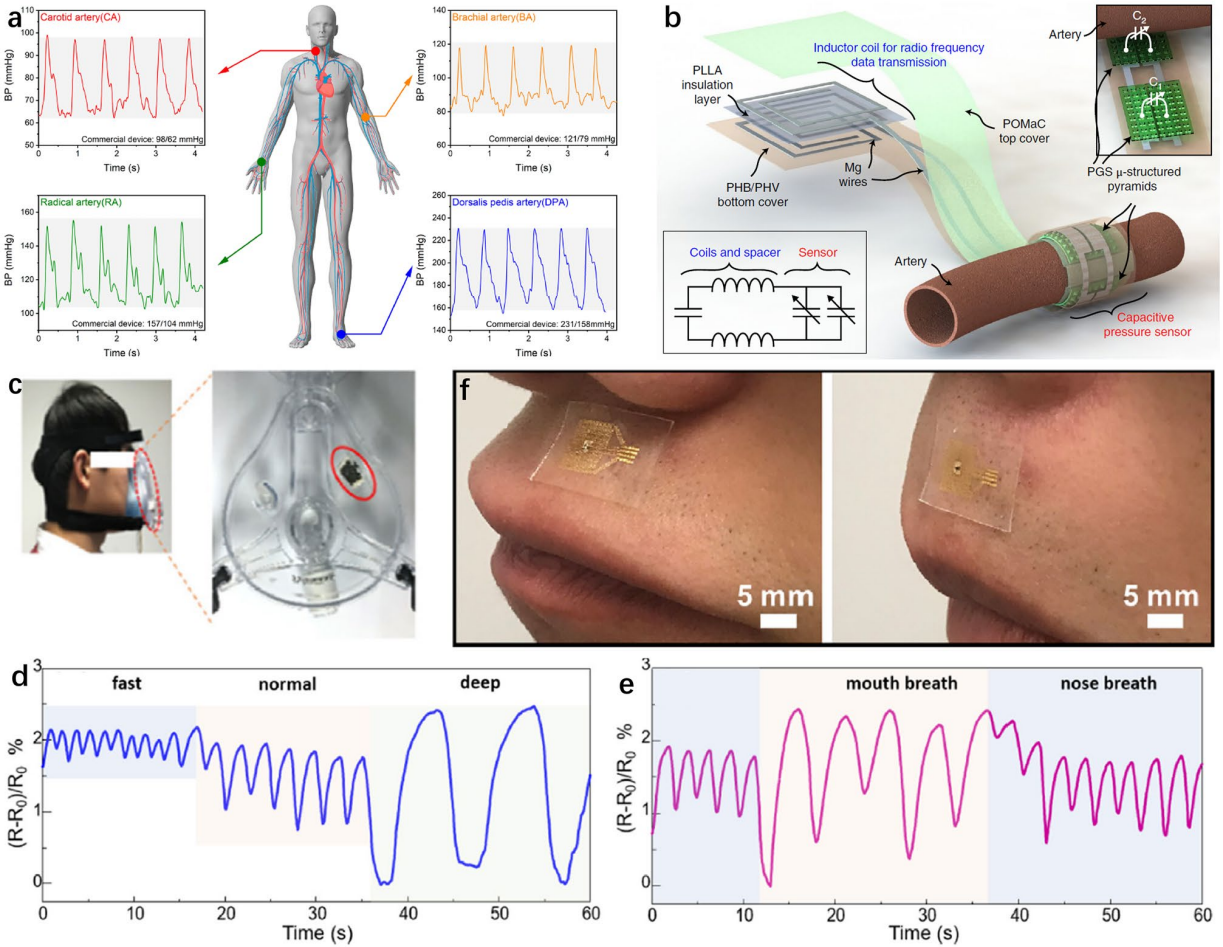
Soft sensors for pulse and respiration. **a** Blood pressure waveforms and values collected at the CA, BA, RA, and DPA. Gray areas indicate the BP range between the SBP and DBP values detected by a commercial sphygmomanometer. Reproduced with permission [226]. Copyright (2020), American Chemical Society. **b** Illustration of the pulse sensor with an exposed view of the bilayer coil structure for wireless data transmission and the cuff-type pulse sensor wrapped around the artery. Inset: Close-up view of the pressure-sensitive region of the sensor with the two variable capacitors C_1_ and C_2_. The two variable capacitors correspond to C_1_ and C_2_. The two inductors are in series with a fixed capacitor. Reproduced with permission [228]. Copyright (2019), Nature Publishing Group. **c** Photograph of a volunteer wearing the medical breathing mask with the humidity sensor fixed inside it. **d** Resistance variation response to fast, normal, and deep breathing. **e** Resistance variation response to mouth and nose breathing. Reproduced with permission [21]. Copyright (2018), Elsevier. **f** Photograph of the respiration sensor on the top of the upper lip with two motions, including pouting (left) and compressing lips (right). Reproduced with permission [237]. Copyright (2020), Elsevier

Wu et al. demonstrated a soft pressure sensor with LSG based on the piezoresistive effect [226]. Different with common pressure sensor, this device has a positive resistance —pressure response. After optimizing the graphene pattern, the relative resistance variation of the sensor can be over 360,000% with good repeatability, and the sensitivity can be up to 434 kPa^−1^. In addition, the mechanical signal can be amplified like a mechanical triode under the external pressure bias. The pulse waves can be collected at the carotid artery (CA), brachial artery (BA), radial artery (RA), and dorsalis pedis artery (DPA), as shown in Fig. 8a. The systolic blood pressure (SBP) and diastolic blood pressure (DBP) can also be deduced from the pulse signals.

Piezoelectric effect is another way to detect the pulse signals. An important advantage of the piezoelectric sensor is the self-power. Chen et al. developed a soft piezoelectric pulse sensor (PPS) based on the single-crystalline group III-nitride thin film, which can be easily transferred and packaged by PDMS [227]. The piezoelectric sensor has good sensitivity. When dropped on a single drop of water with a mass of 38 ± 4 mg, the sensor can generate a voltage of about 20 mV. With the piezoelectric sensor, pulse waveform was measured at the carotid, brachial, radial, femoral, posterior tibial, dorsalis pedis, femoral, popliteal, fingertip, facial, and temporal artery.

Piezocapacitive effect is also a significate method of fabricating pressure sensors. Boutry et al. reported a piezocapacitive pressure sensor with biodegradable materials (Fig. 7b) [228]. The arterial blood flow can be detected in both contact and non-contact modes. Poly (glycerol sebacate) (PGS) with pyramidal patterns was applied as the dielectric layer. After the pressure loaded, the capacitance would be changed, leading to the shift of resonance frequency of the system consisting of the inductance and the piezocapacitive pressure sensor. More importantly, the sensor can realize the in vivo arterial pulse monitoring of the rat femoral artery.

### Respiration

The total process of gas exchange between human body and external environment is called respiration. In the calm state, the normal respiratory rate of adults is about 16–20 times min^−1^. The monitoring and detection of human respiration is an important part of modern medical monitoring technology, especially in the treating of the COVID-2019 [22]. There are three typical sensors to detect the respiration signal, mechanical sensor [102], humidity sensor [21], and temperature sensor [237]. Some parameters of respiratory are shown in Table 2.

**Table 2:** Typical respiration pulse sensor

Physiological signal	Device type	Active material	Signal type	Mechanism	Detecting source	References
Respiration	Strain sensor	Metal cotton fibers	Capacitance	Piezocapacitive	Belly	[240]
Respiration	Humidity and gas sensor	Acidified carbon nanotube/PU nanofibers	Resistance	Electron transfer	Mask	[239]
Respiration	Humidity sensor	Graphene/GO Graphene/Ag colloids Graphene/PEDOT:PSS	Resistance	Molecular adsorption and desorption	Mask	[21]
Respiration	Pressure sensor	Graphene	Resistance	Piezoresistive	Mask	[102]
Respiration	Strain sensor	Graphene	Resistance	Piezoresistive	Mask, Throat, Top of the upper lip	[233]
Respiration	Strain and pressure sensor	Graphene foam	Resistance	Piezoresistive	Mask, Heart area, and Lung cavity	[236]
Respiration	Pressure sensor	Graphene	Resistance	Piezoresistive	Chest	[104]
Respiration	Humidity sensor	GO coated silk fibers	Current	Proton transfer	Mask	[241]
Respiration	Nanogenerators	PVDF	Voltage	Pyroelectric	N95 respirator	[242]
Respiration	Thermistor	N.A	Resistance	Thermal convection effect	Top of the upper lip	[237]
Respiration	Humidity and gas sensor	Ce-doped ZnO	Voltage	Triboelectric	Mask	[238]
Respiration	TENG	Ce-doped ZnO-PANI	Voltage	Triboelectric	Elastic balloon	[243]

During the respiration process, the thorax expands when inhaling and shrinks when exhaling driven by the intercostal and diaphragmatic muscles. In addition, there will be pressure changes around the mouth or nose due to the flow of air. Therefore, the mechanical sensor can also be used to monitor the respiration signal, which is similar to the pulse sensor. Triboelectric nanogenerator (TENG) is another self-power device which can be utilized as not only energy harvester but also mechanical sensor. It relies on static electricity generated by friction between two materials to drive the flow of electrons and generate electricity. Wang et al. realized an integrated triboelectric respiration sensor for monitoring human respiration and NH_3_ concentration in exhaled gases [238]. Ce-doped ZnO and PDMS were used as the triboelectric layer and Au were coated as electrodes. The dissolution of water molecules into Ce-doped ZnO would enhance the relative permittivity of sensitive material and decrease the output voltage. Some gases such as NH_3_, CH_4_, CH_2_O, C_2_H_5_OH, and CO can also be detected.

Humidity sensor based on the electron transferring rather than mechanical interaction can also be used as the respiration sensor. Pang et al. fabricated the graphene on the nickel foam by CVD [21]. After etching the nickel, porous graphene networks were obtained. To enhance the sensing performance, the porous graphene was modified by the GO, PEDOT:PSS, and Ag colloids. The air flow force has no effect to the humidity sensor. Different breathing modes, such as slow, fast, deep, and normal, can be distinguished. The device can also be fixed inside the medical breathing mask to detect the respiration in real time (Fig. 8c, d). Huang et al. realized a soft, stretchable, and conductive nanofiber composite with acidified CNT decorated PU nanofiber [239]. As mentioned above, CNT is a typical *p*-type semiconductor. Electrons transferred from H_2_O molecules would reduce the density of holes in CNT and lead to the increase of the resistance. In addition, during a sensing test cycle, swelling and de-swelling caused by the H_2_O molecules would damage and recover the conductive network, also lead to the increase and decrease of the resistance. Other gas such as methanol, heptane, and acetone can also be detected.

During inhalation and exhalation, the temperature in the nasal cavity will change accordingly. Therefore, the temperature sensor can also be applied to detect the respiration signal. Liu et al. developed a respiration sensor based on metallic heating electrode (Cr/Au), thermistor, and PDMS package, as shown in Fig. 8e [237]. By adjusting the input power of heating electrode and increasing the temperature difference between the respiration sensor and environment, the sensitivity of the respiration sensor can be improved. In addition, various breathing patterns can be distinguished with the breath rate/depth of subjects, such as sitting, frightening, sleeping, meditating, and gasping.

### Human Motion

Human motion signals including motions of the arm, hand, foot, knee, etc. Normally, the limbs would have large-strain changes during human activities. For example, walking is accompanied by the knee bending, arm swing, and foot compressing. More importantly, the detection of those motion signals can not only provide health care evaluation on our daily life, but also useful for posture correction in the rehabilitation treatment [244]. According to the position of limbs deformation, it can be classified into two major categories: (i) bending or stretching of arm or hand on the upper limb; (ii) bending and compressing of the knee or under the foot. The limbs bending and foot walking usually display large-strain variation and high-pressure impact, respectively. The strain and pressure sensors with large measuring range should be developed. To date, the strain sensors were widely used to monitor the large bending of elbow [245], wrist [246], finger [247], and knee [44, 103, 105, 224], while the pressure sensor with high measuring range were used to detect the different walking states [102, 248, 249].

**Fig. 9:**
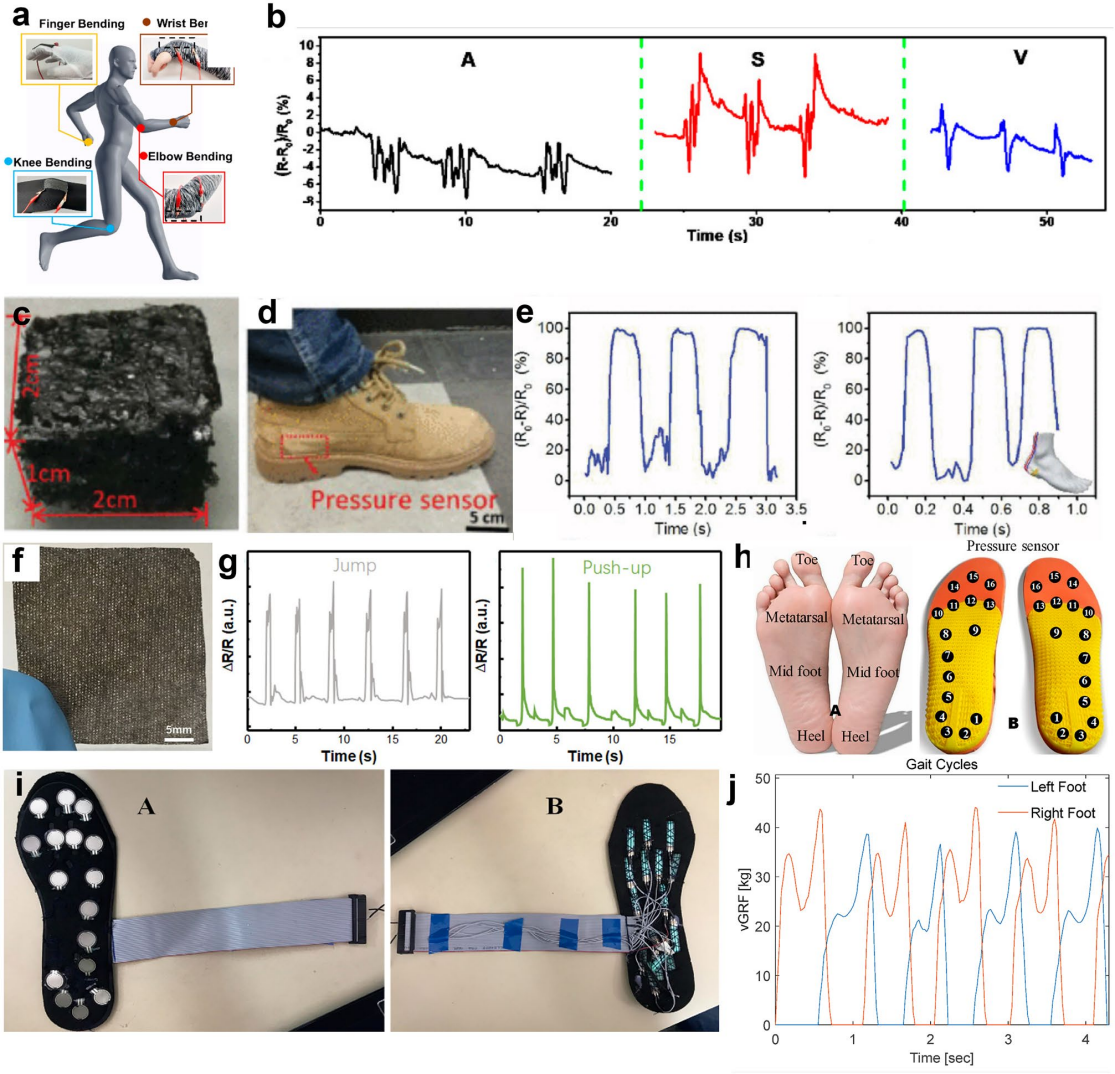
Soft sensors for human motion. **a** Detection of various human motions using the wearable graphene textile strain sensor. **b** Detection of writing English letters. Reproduced with permission [103]. Copyright (2018), American Chemical Society. **c** Photograph of the honeycomb-like graphene composite structure. **d** Photograph of the foot with the HGN pressure sensor. **e** Response of the HGN pressure sensor under walking and running. Reproduced with permission [248]. Copyright (2020), Institute of Electrical and Electronics Engineers. **f** Tissue paper with rGO. **g** Response curves for the tester’s movements of jumping and push-ups. Reproduced with permission [102]. Copyright (2017), American Chemical Society. **h** Area of foot selected for sensors and **i** array of pressure sensor. **j** Gait cycles readings for left and right foot with FSR smart insole. Reproduced with permission [249]. Copyright (2020), MDPI

The most frequently used limbs on the human body are supposed to be the arms and hands during the whole life. Almost all the actives involve the arm bending, and hand holding or releasing. Thus, it is necessary to develop highly sensitive, stable, reproducible, and durable strain sensors for this specific application. Yamada et al. reported a class of wearable and stretchable devices fabricated using thin films of aligned CNTs [44]. When stretched, the CNTs films were fractured into gaps, islands, and bundles bridging the gaps. This mechanism allows the films to be strain sensors with the measuring strains up to 280%, with high durability, fast response, and low creep. The CNTs sensors were assembled on stockings, bandages, and gloves to detect the movement. Yang et al. proposed a AgNPs bridged graphene strain sensor for simultaneously detecting subtle and intensive human motions [105]. AgNPs serve as the bridges to connect the self-overlapping graphene sheets, which endows the strain sensor with many excellent performances. With high GF of 475, it is suitable to be applied in human motion detection. Then, they fabricated a close-fitting and wearable graphene textile strain sensor based on a graphene textile without polymer encapsulation [103]. GO acts as a colorant to decorate the polyester fabric and is reduced by high temperature, which endows the graphene textile strain sensor with excellent performance. Compared with other strain sensors, the textile strain sensor exhibits a distinctive negative resistance variation with increasing strain. The graphene textile strain sensor can be knitted on clothing for detecting both subtle and large human motions, as shown in Fig. 9a, b. The wrist guard integrated with the graphene textile strain sensor can monitor wrist movement, including the resistance change with different English letters, such as “A”, “S”, and “V”. It can also be knitted on a single glove to monitor the response toward the bending of a finger.

Walking is a basic capability that allows the human to carry out daily activities. When someone suffers the pathological gait disease, the motion capture technology can provide quantitative features for its analysis and diagnosis [250]. Generally, the walking states could be monitored by the pressure sensor under the foot or strain sensor on the knee. For the pressure sensor, it measures the force directly caused by the periodic foot stepping. Due to the body weight, pressure sensors with proper sensitivity in a wide pressure range are desirable. Besides, high flexibility and low thickness would bring a comfortable experience for long-term monitoring. Tian et al. fabricated a soft pressure sensor based on the honeycomb-like graphene network (HGN) by mixing cube sugar, graphene oxide solution, and PDMS (Fig. 9c, d) [248]. Attributing to the structure, the pressure working range can cover from 0 to 150 kPa. Benefiting from its advantages in pressure range, it shows good performance when monitoring working or running form (Fig. 9e). Based on the paper tissue, Tao et al. fabricated a graphene/paper pressure sensor (Fig. 9f), which can be applied in intense motion detections [102]. As shown in Fig. 9g, the sensor shows the difference between jumping and push-ups. For the systematic approach to detection and analysis gait, Tahir et al. provided a systematic design and characterization procedure for three different pressure sensors including a soft piezoelectric sensor, which can be used for detecting vertical ground reaction forces using a smart insole [249]. The pressure sensor array was placed in a customized shoe above the control circuit. Pressure data were digitized through a microcontroller before sent wirelessly to a host computer for post processing and analysis. The subsystem was powered by a battery with the help of a power management unit. Pressure data were analyzed to extract various gait characteristics for different gait applications. As shown in Fig. 9h, i, sixteen sensors were placed on each insole to record pressure values in these areas, the inputs were multiplexed to one output through a 16-to-1 multiplexer and applied to an analog-to-digital (ADC) conversion input of the microcontroller then sent to host computer. The gait cycle of 12 subjects were recorded while walking on a 10 m walkway in self-selected walking manner. In Fig. 9j, the gait cycles readings for left and right foot were clearly recorded by the system, and can be further analyzed for assessing walking behaviors.

### Intraocular Pressure

IOP is the prime indicator for the diagnosis and treatment of glaucoma, which has circadian rhythm changes and depends on body gestures. Therefore, a single measurement in the clinic can be misleading for diagnosis. Contact lenses as a minimally invasive platform for diagnostics and drug delivery have emerged in recent years [251, 252]. Contact lens sensors have been developed for analyzing the glucose composition of tears as a surrogate for blood glucose monitoring and for the diagnosis of glaucoma by measuring intraocular pressure [253].

**Fig. 10:**
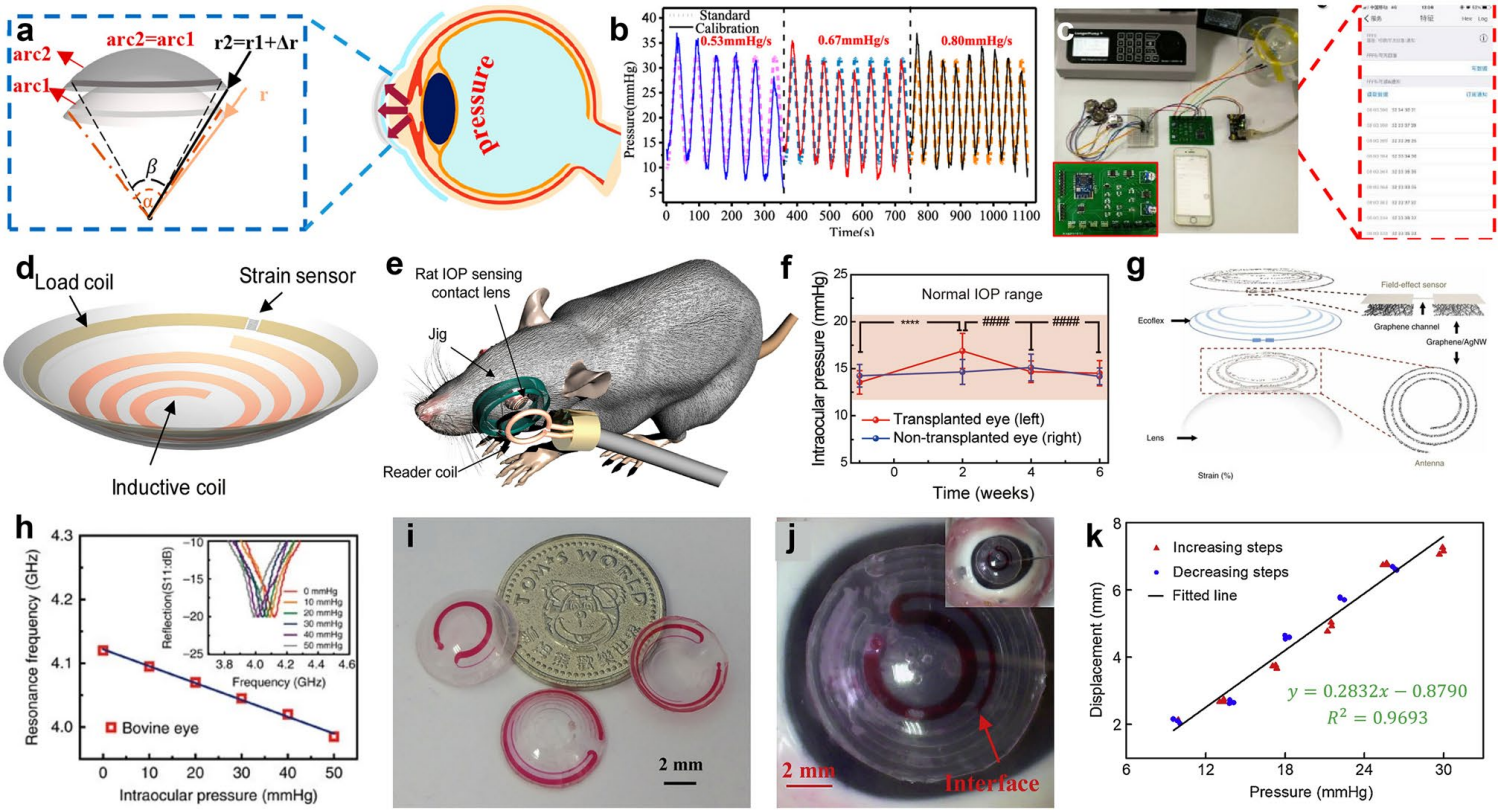
Soft sensors for IOP. **a** Schematic diagram of the change of the IOP sensor’s radius of curvature caused by the increase of IOP. **b** Comparison of calibrated IOP with the standard values at the speed of 0.53, 0.67, and 0.8 mmHg s^−1^, respectively. **c** Measurements of graphene IOP sensor with portable wireless system. Reproduced with permission [90]. Copyright (2020), American Chemical Society. **d** Schematic of the strain sensor-based contact lens. **e** Wireless real-time measurement of IOP in live rat. **f** Measurements of the IOP of normoglycemic Lewis rats. Reproduced with permission [254]. Copyright (2020), American Chemical Society. **g** Schematic of the wearable contact lens sensor, integrating the glucose sensor and IOP sensor. **h** Frequency response of the IOP sensor on the bovine eye from 5 to 50 mmHg. Reproduced with permission [155]. Copyright (2017), Nature Publishing Group. **i** Photograph of actual fabricated microfluidic contact lenses. **j** Photograph of the microfluidic contact lens wearing on the porcine eye ex vivo. **k** Displacement response of the devices on porcine eye. Reproduced with permission [255]. Copyright (2019), Elsevier

Recently, Xu et al. realized a noninvasive sensor with few-layer graphene (Fig. 10a) [90], and it shows high transparency, sensitivity, linearity, and biocompatibility for 24 h continuous IOP monitoring. The graphene Wheatstone bridge consisting of two strain gauges and two compensating resistors is designed to improve the sensitivity and accuracy of IOP measurement. Testing results on a silicone eyeball model indicate that the output voltage of the sensor is proportional to the IOP fluctuation. Under the various ranges and speeds of IOP fluctuation, the sensor exhibits excellent performance of dynamic cycles and step responses with an average sensitivity of 150 μV mmHg^−1^ (Fig. 10b). With the linear relationship, the average relative error between the calibrated IOP and the standard pressure in maintained at about 5%. Furthermore, a wireless system is designed for the sensor to realize IOP monitoring using a mobile phone (Fig. 10c). This sensor, with the average transparency of 85% and its ease of fabrication, as well as its portability of continuous IOP monitoring, brings new promise to the diagnosis and treatment of glaucoma. Intraocular islet transplantation was investigated as a new procedure to treat diabetes, the development of this procedure requires close monitoring of the function of both eye and islet graft. Based on this, Kim et al. developed a soft, smart contact lens to monitor the intraocular pressure and applied this for noninvasive monitoring (Fig. 10d) [254]. A strain sensor inside the lens can detect detailed changes in IOP by focusing the strain only in the selective area of the contact lens. In addition, this smart contact lens can transmit the real-time value of the IOP wirelessly using an antenna. The wireless measurement of IOP obtained using the contact lens has a high correlation with the IOP measured by a rebound tonometer, which proving the good accuracy of the contact lens sensor. The feasibility of the contact lens platform was tested in a rat animal model (Fig. 10e). After the transplantation, a marginal increase in IOP could be detected, and it returned to normal within a few weeks (Fig. 10f). Pang et al. fabricated a contact lens with metal electrode Wheatstone bridge circuit for noninvasive monitoring of IOP [23]. With the excellent dynamic cycling performance at different speeds of IOP variation, the contact lens sensor is promising for continuous IOP monitoring of glaucoma disease, regardless of the posture and activities of the patient. Kim et al. developed a contact lens sensor which can measure the glucose level in tear fluid and IOP simultaneously (Fig. 10g) [155]. They fabricated the strain sensor and glucose field effect sensor on the contact lens. Utilizing the two strain gauges and dielectric silicone to form sandwich structure, the inductance and capacitance circuit can transmit the IOP into variation of resonance frequency. On the bovine eyeball testing (Fig. 10h), it exhibits decreased linearity with pressure increasing and slope of 2.64 MHz mmHg^−1^. This wearable contact lens would be promising technique to monitor the IOP in wireless and real time. An et al. designed a microfluidic contact lens for unpowered continuous and noninvasive IOP monitoring (Fig. 10i) [255]. The microfluidic contact lens is comprised of a sensing layer of the micropatterned soft-elastomer and a hard-plastic reference layer. The device uses the annular sensing chamber filled with the dyed liquid and a sensing microchannel as the IOP transducer (Fig. 10j). The maximum sensitivity of the device (with the sensing chamber of 8.5 mm in diameter and the sensing channel of 100 × 40 μm^2^ in size) can achieve 0.708 mm mmHg^−1^ in the working range of 0–40 mmHg (Fig. 10k). By using theoretical analyses and experimental investigations, the IOP sensing mechanism on curved surface of the devices with different dimension parameters are explored, the test on enucleated porcine eyes show that the devices have a linear response and can track the IOP changes.

### Phonation

**Fig. 11:**
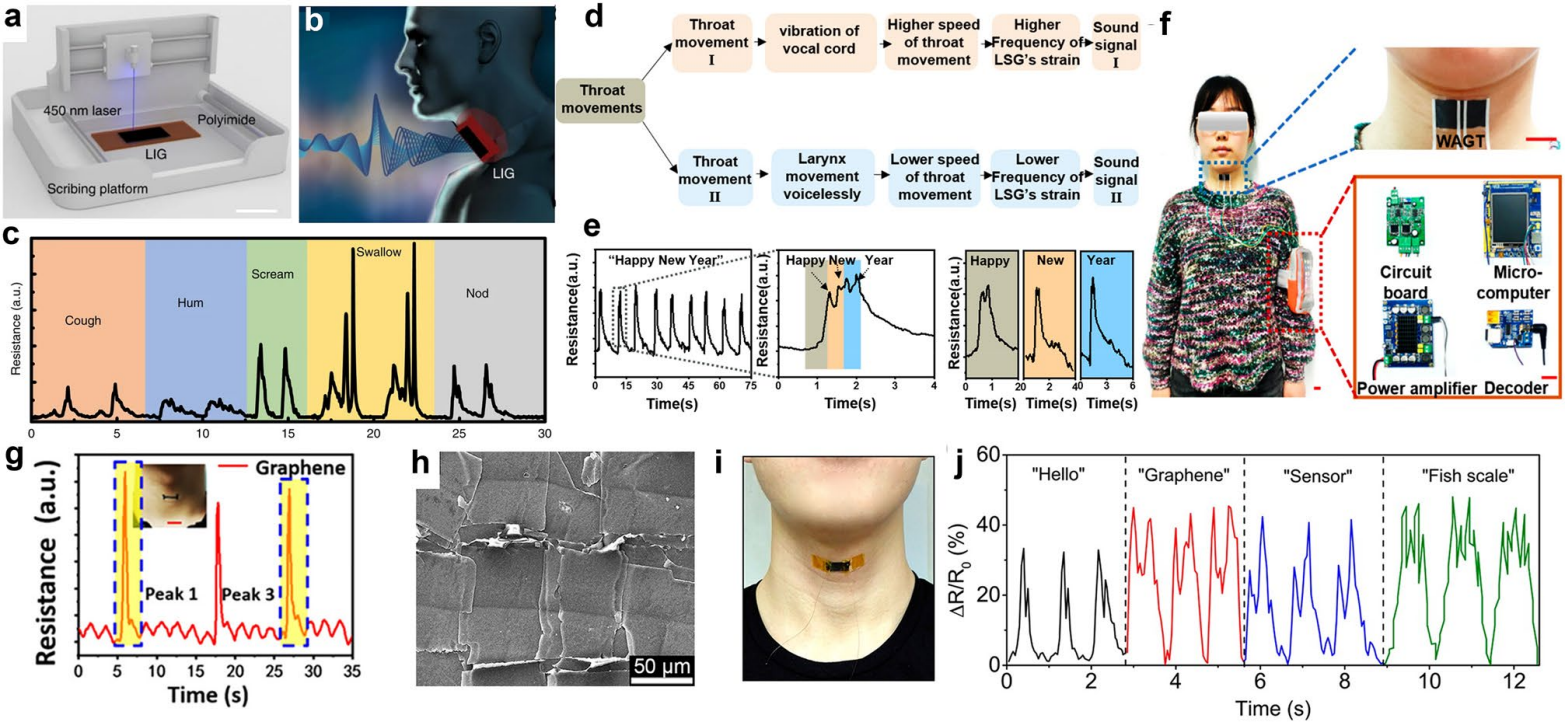
Soft artificial throat. **a** One-step fabrication process of LIG. **b** Artificial throat can detect the movement of throat and generate controllable sound, respectively. **c** LIG’s resistance changes toward the throat vibrations of the tester who makes two successive coughs, hums, screams, swallowing, and nods. Reproduced with permission [101]. Copyright (2017), Nature Publishing Group. **d** Frame diagram of how different throat movements were transformed to different sound signals. **e** Response of attached LSG toward sound, “Happy New Year”. One wave curve is magnified to be showed. **f** Artificial throat system worn by a tester. Reproduced with permission [256]. Copyright (2019), American Chemical Society. **g** Response toward the sound “graphene” and its magnified image. Inset: throat with an attached sensor. Reproduced with permission [233]. Copyright (2018), American Chemical Society. **h** Top-view SEM images of a fish-scale-like graphene strain sensor. **i** Photograph of the sensor attached to the throat of a person. **j** Responsive curves recorded during the processes of speaking “hello”, “graphene”, “sensor”, and “fish scale”, respectively. Reproduced with permission [257]. Copyright (2016), American Chemical Society

Phonation is the process by which the vocal folds produce certain sounds through quasi-periodic vibration, which is the most direct way to communicate with each other. During the voicing process, the pressure drop across the larynx can induce oscillation of vocal folds. The slight throat motion would be accompanied by the phonation. In general, it shows the characteristic signals for different throat motions, which can be used to record the word or speech [101, 256]. The features of vocalization signals include loudness, jitter, fundamental frequency, zero-crossing rate, and energy frequency ratios. For the throat motion detection, a high-sensitive, microscale, and soft strain or pressure sensor is an ideal candidate due to the small strain change for the thousands of vocabularies. Tao et al. proposed an intelligent artificial throat based on LIG [101], which can not only generate but also detect sound in a single device (Fig. 11a, b). The LIG’s resistance changes toward the throat vibrations of the tester who makes two successive coughs, hums, screams, swallowing, and nods (Fig. 11c). Wei et al. further developed a wearable skin-like ultrasensitive artificial graphene throat (WAGT) system integrated both sound/motion detection and sound emission in single device (Fig. 11d) [256]. The WAGT has a high detection sensitivity and an excellent sound-emitting ability. For sound detection, both the motion of larynx and vibration of vocal cord contribute to throat movements. Meanwhile, different human motions, such as strong and small throat movements, were also detected and transformed into different sounds like “OK” and “NO”. Therefore, the implementation of these sound/motion detection acoustic systems enables graphene to achieve device-level applications to system-level applications (Fig. 11e, f), and the graphene acoustic systems are wearable for its miniaturization and small weight. Qiao et al. demonstrated a multilayer graphene epidermal e-skin (Fig. 11g) [233]. When packed in Ecoflex, e-skin exhibits excellent performance, including ultrahigh sensitivity, large strain range, and long-term stability. Therefore, the physiological signals like phonation can be detected based on epidermal e-skin with a single graphene line pattern. Qiang et al. reported a high-performance strain sensor with a fish-scale-like graphene-sensing layer (Fig. 11h) [257]. This strain sensor can be fabricated via stretching/releasing the composite films of rGO and elastic tape, making the process simple, low cost, energy-saving, and scalable. When attached to the throat, it can detect the complicated epidermis/muscle movements during speaking (Fig. 11i). The sensor shows characteristic and repeatable signal pattern when the wearer spoke different words, such as “hello”, “graphene”, “sensor”, and “fish scale” (Fig. 11j).

### Tactile Sensation

Tactile sensation is the sensation produced by human skin when it touches physical stimuli (such as pressure, temperature, humidity, and vibration). When human skin tactile receptors receive appropriate stimuli from the external environment, action potentials are generated, which are transmitted to the cerebral cortex through nerve conduction pathways and generate corresponding perception [258]. E-skin can realize the interaction between machines or between humans and machines [259]. The main function of the soft tactile sensor is to detect the physical properties of the manipulated object or identify the manipulated object or operation state. Generally, the principle of sensing can be summed up as the measurement of strain, pressure, displacement, torsional deformation, and other parameters, and thus a series of piezoresistive, piezocapacitive, piezoelectric, and triboelectric sensors are proposed [260].

**Fig. 12:**
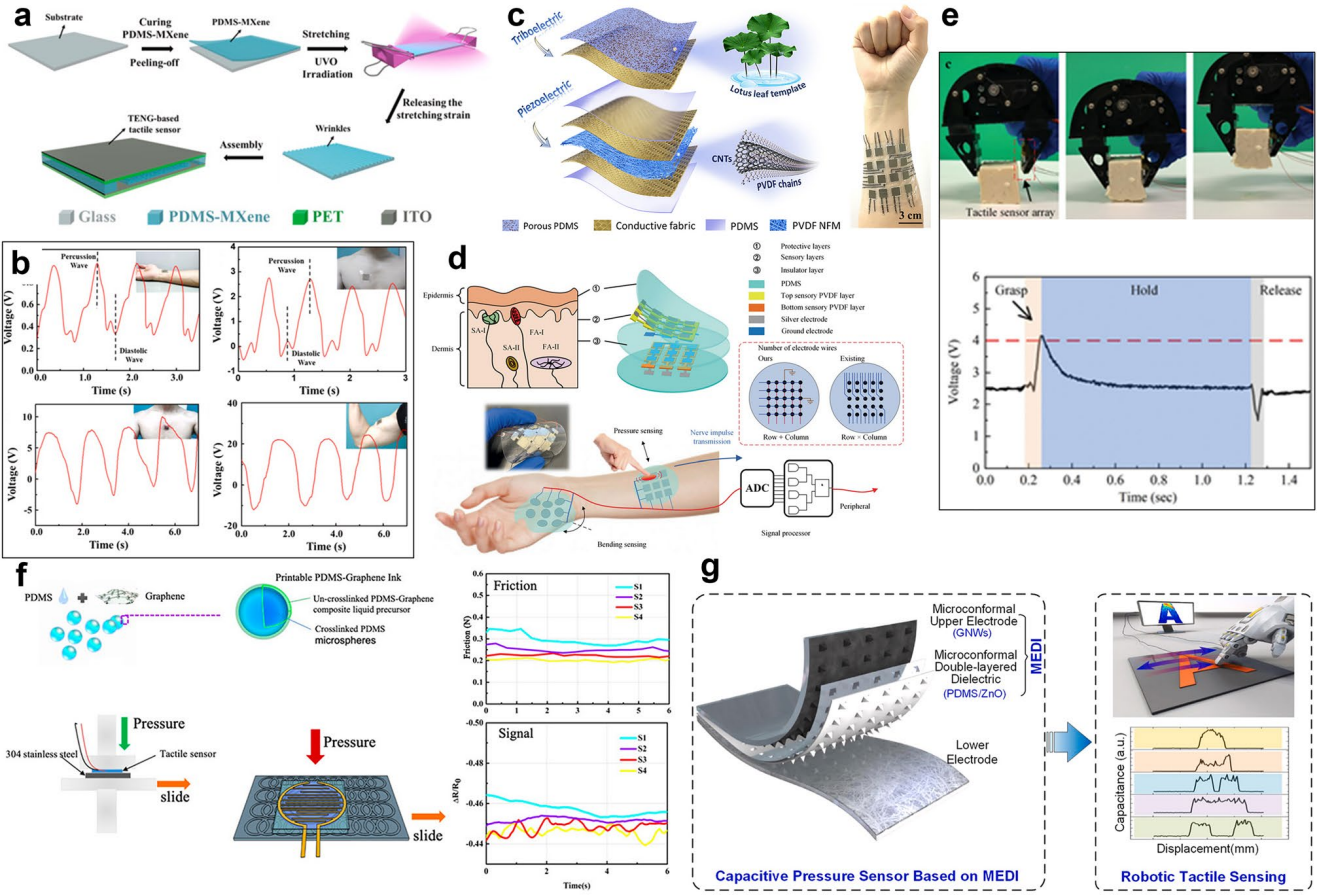
Soft tactile sensation sensor. **a** Schematic diagram of the triboelectric tactile sensor fabrication process. **b** Voltage–time waveform of the sensor under pulse, heartbeat, breath, and flexion and extension of biceps. Reproduced with permission [262]. Copyright (2021), Elsevier. **c** Schematic diagram of the battery-free e-skin structure. Reproduced with permission [263]. Copyright (2021), Elsevier. **d** Schematic diagram of the skin-inspired piezoelectric tactile sensor array. A large number of sensor pixels can be fabricated with a small quantity of wires. **e** Sensor array placed on the robot hand and the tofu block grasped by real-time feedback. Reproduced with permission [264]. Copyright (2021), Wiley–VCH. **f** Response of the graphene-PDMS microsphere structure tactile sensor to items with different roughness. Reproduced with permission [266]. Copyright (2021), American Chemical Society. **g** Schematic diagram of soft piezocapacitive sensor structure and slipping imaging of surfaces with different roughness. Reproduced with permission [267]. Copyright (2021), Elsevier

At present, many studies focus on triboelectric sensors, because of its self-powered advantage [261]. Cai et al. proposed a self-powered tactile sensor based on triboelectric [262]. Through ultraviolet ozone radiation and tensile treatment, regular folds are generated in PDMS/MXene composite film (Fig. 12a), which greatly increases the contact area and sensitivity of the friction layer. High sensitivity of 0.18 and 0.06 V Pa^−1^ are achieved in the range of 10–80 and 80–800 Pa, respectively. The sensor can distinguish four different movements (pulse, heartbeat, breath, flexion, and extension of biceps) and different weights by output voltage waveform and amplitude (Fig. 12b).

Compared with triboelectric effect, piezoelectric effect can monitor the continuous deformation process under contact condition. Zhu et al. proposed a self-powered hybrid electronic skin (HES) based on triboelectric effect and piezoelectric effect (Fig. 12c) [263]. A sensor unit is only 82.6 mg and can be kept stable at any bending, so it can be directly attached to the skin as HES sensor array. Since it combines the advantages of triboelectric effect and piezoelectric effect, it can realize sensing in the entire contact, separation, deformation, and recovery process. It has been successfully applied to measure click, distance, respiration, head motion, vocal cord vibration, and some physiological signals. It can also identify multi-point pressure distribution and realize real-time single-point touch trajectory visualization. Lin et al. proposed a soft piezoelectric tactile sensor array that can perceive and distinguish the size, position, and pattern of different external stimuli in real time [264]. In order to eliminate crosstalk interference and reduce the number of wires, they designed a comb sensor connection mode without crossing. For *n* × *m* sensor array, only *n* + *m* wires are required (Fig. 12d). The sensor consists of two protective layers (100 μm PDMS film), two silver-plated PVDF sensory layers, and one insulative layer (500 μm PDMS film) to simulate the human skin structure. The sensor can detect a weak carotid pulse and also accurately pick up the movement of a 5 mg spider, including position, resting time, and duration of passage. When mounted on a robotic hand, the feedback can accurately guide and complete a series of operations to grasp, hold, and release objects (Fig. 12e).

In addition to motion measurement, morphology roughness is also a key parameter for e-skin to obtain subject information [265]. Wang et al. proposed a 3D printed soft piezoresistive tactile sensor based on graphene-PDMS microspheres to simulate the structure of human finger fingerprints (Fig. 12f). It can not only monitor pressure, but also detect different degrees of surface roughness, and detect air fluids [266]. The sensor has a short response time of 60 ms and a sensitivity of up to 2.4 kPa^−1^ at low pressure. The sliding test shows that the surface of stainless steel with average roughness of 0.959 ± 0.005, 0.826 ± 0.08, 0.811 ± 0.04, and 0.785 ± 0.04 μm can be resolved. Luo et al. demonstrated a soft piezocapacitive sensor using a microstructured graphene nanotube (GNWs) electrode and a conformal microstructured dielectric layer (Fig. 11g) [267]. The top pyramid-structured electrode of the capacitor includes GNWs/PDMS/ZnO prepared through conformal growth and replica transfer methods, and the bottom is a plate electrode dielectric including PMMA layer and AgNWs. The microstructure of electrode and dielectric layer can not only avoid the slip between electrodes and improve the stability, but also increase the area of the air-gap dielectrics and zinc oxide film to enhance the polarized electric field, so as to improve the piezocapacitive effect of the sensor. The sensor was applied as the smart glove to successfully distinguish the amount of water in the cup and realized braille recognition and roughness detection.

### Heart Sounds

Heart sounds are the sounds produced by the heart muscle as it contracts, the heart valves and the blood hitting the blood vessel walls during heart contraction and relaxation. During this process, the signal is collected through a chest microphone or a digital stethoscope and the resulting image of the wave amplitude over time is recorded, which is called phonocardiogram (PCG) [268].

Normally, the first and second heart sounds can be heard, as well as the third heart sound that occurs in children and adolescents. But in pathological conditions, third heart sounds, fourth heart sounds, and various murmurs can also be heard in non-adolescents [269]. Because abnormal heart sounds under pathological conditions contain a lot of information about heart valves, such as arrhythmias, valvular heart diseases, and heart failure [270], cardiac auscultation is often an important basis for the early detection of cardiovascular diseases. The wearable heart sound sensor can avoid frictional sounds in the process of auscultation with the traditional stethoscope, and can carry out continuous monitoring. With the help of computers, automatic heart sound analysis does not depend on the skills, experience, and subjective feelings of the health care provider [271]. Heart sound is a signal that can be acquired in vitro and can be extracted in real time, but the signal is weak. In addition, the environmental noise is easy to be introduced in the process of signal collection, and there are many noises and motion artifacts, so high sensitivity and high SNR are required for the sensor.

**Fig. 13:**
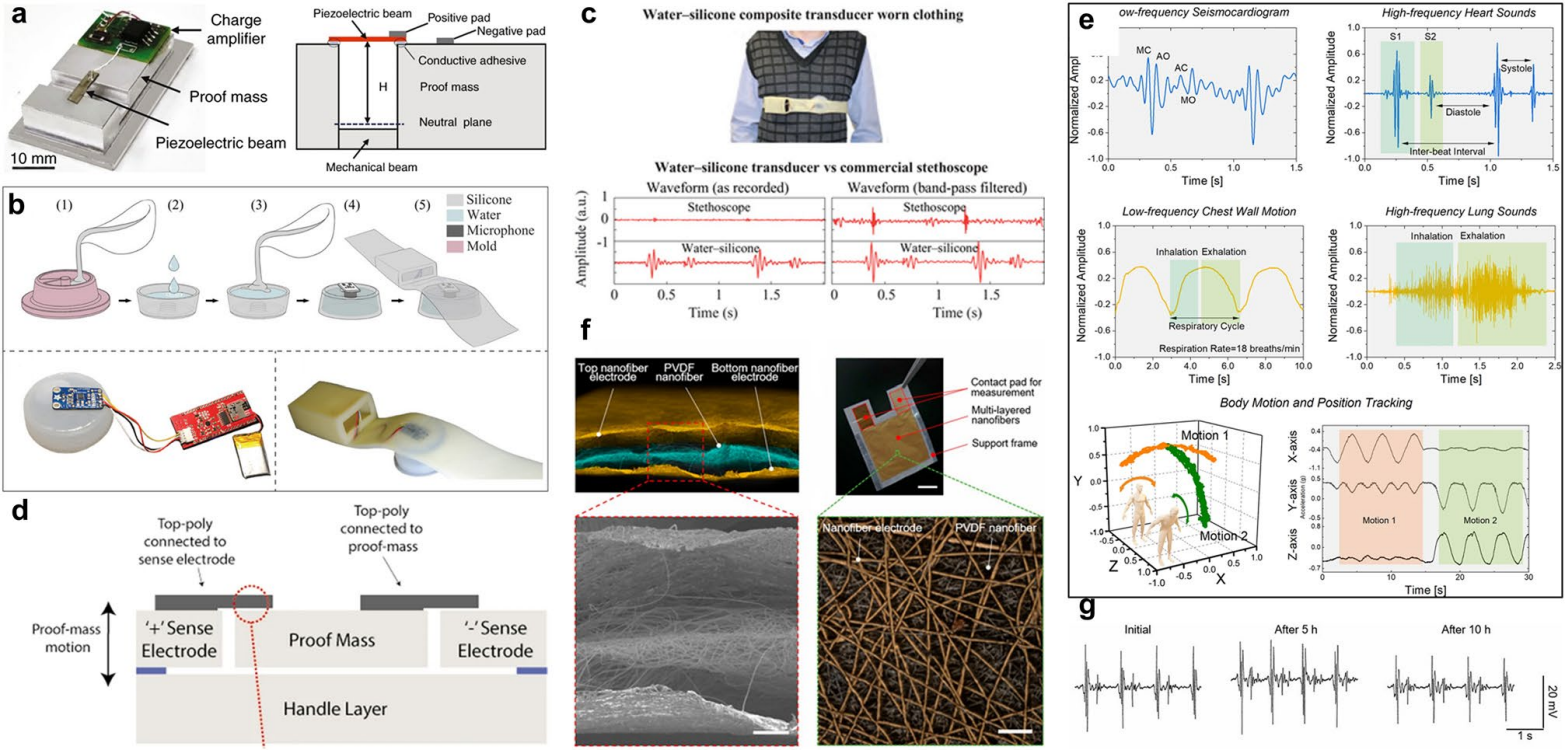
Wearable heart sound sensor. **a** Inside view of the prototype with a printed circuit board and structure of the accelerometer-based on an asymmetric gapped cantilever structure. Reproduced with permission [273]. Copyright (2021), Nature Publishing Group. **b** Preparation of the water–silicone composite materials and integration with acoustic sensors. **c** Monitoring heart sounds while clothed and comparison of water–silicone based sensor and commercial stethoscope for heart sounds detecting. Reproduced with permission [274]. Copyright (2020), Wiley–VCH. **d** Cross-sectional view of the sensor showing the ultrathin capacitive gaps, satisfying low noise and broadband. **e** Recording of cardiopulmonary vibrations, sounds, and body motion. Reproduced with permission [276]. Copyright (2021), Nature Publishing Group. **f** Structure of all-nanofiber mechanoacoustic sensor. **g** Sensor allowing for more than 10 h of continuous monitoring. Reproduced with permission [277]. Copyright (2020), National Academy of Sciences

Chen et al. based on previous studies [272], present a small-sized, ultrasensitive accelerometer for continuous monitoring of lung and heart sounds to assess the lung and heart status of patients [273]. Thanks to two-stage amplification of the asymmetric gapped cantilever structures and electric charge amplifier (Fig. 13a), the sensitivity of the sensor can meet the extraction of weak heart sound signals. Theoretical simulations showed that the response of the modified structure was about 9.7 times that of a commercially available high-end electric stethoscope with a conventional cantilever structure, and continuous monitoring of patient heart sounds was consistent with clinical reports.

In order to reduce motion artifacts, heart sound monitoring puts forward higher requirements for sensor performance. To solve this problem, Cotur et al. proposed a stretchable and mechanically robust low-cost soft composite composed of silicone and hydrogel, which can be used as acoustic propagation medium to significantly reduce noise and continuously monitor PCG in the presence of clothing isolation (Fig. 13b, c) [274]. Even in the case of exercise, the recorded PCG did not change significantly due to improved contact between the sensor and human skin.

Usually, in order to obtain more physiological information, the extraction of heart sounds and other physiological signals is often integrated in the same wearable device [275]. Gupta et al. combined the accelerometer and microphone, and presented a wearable sealed vibration sensor with high sensitivity (Fig. 13d). Due to its wide frequency range (up to 12 kHz), it can simultaneously monitor heart and respiratory frequencies, heart sounds, lung sounds, and body movements (Fig. 13e). In addition, weak pathological heart sounds were detected in patients with cardiopulmonary diseases [276]. Nayeem et al. obtained three nanofiber layers by electrospinning process [277]. The upper and lower layers were PU nanomesh sheets deposited with polyethylene and Au, and the middle layer was an ultrathin (2.5 μm) PVDF nanofiber electrode layer (Fig. 13f). Then, an all-nanomesh mechanoacoustic sensor was prepared by stacking the three together. The nanomesh structure significantly reduces its density, the total weight is as small as 5 mg, and it exhibits good air permeability and cyclic bending performance, showing a sensitivity of 10,050.6 mV Pa^−1^ in the frequency range of heart sounds (< 500 Hz). Due to the inherent piezoelectric effect of PVDF and the triboelectric effect between PVDF and the upper and lower layers of nanofibers, the sensor does not require external power supply and is suitable for long-term monitoring (> 10 h) of cardiopulmonary signals (Fig. 13g).

### Electrophysiological Signals

With the intensification of population aging in many contourites, the incidence rate of cardiovascular and cerebrovascular diseases is increasing. Therefore, an increasing amount of research has been focused on real-time sustainable long-term health monitoring. Electrophysiological signals monitoring has become an important research branch. Electrophysiological signals include ECG, EEG, EMG, and EOG, etc. Among them, ECG refers to the trend chart of the potential changes of the myocardial cell membrane inside and outside the myocardial cell membrane due to the change of the cell membrane permeability when the cardiomyocyte is stimulated by a certain intensity. ECG signal can also be used for arrhythmia, fatigue, sleepiness monitoring, etc. [278].

EEG is a graph to reflect electrophysiological changes in brain nerve cells. It can be used to migraine [279] and emotion [280] recognition. In addition to using ECG signal to detect non-convulsive seizures [281], EEG can be used to predict epilepsy [282]. Moreover, Cao et al. used the dynamic changes of the frontal lobe EEG to study the response of patients with refractory depression (TRD) to ketamine [283]. Combined with the measurement of the Hamilton Depression Scale score, it is expected to be used for depression real-time inspection and treatment.

When a potential appears on both sides of the exciting muscle cell membrane and conducts along the cell membrane to the deep part of the cell, the muscle cell contracts and generates a weak current. The graph of the current intensity over time is called EMG. Among them, surface EMG (sEMG) is a synthesis of the potentials of multiple motor units and does not need to invade the skin for measurement. Therefore, it is often used in wearable devices. The EMG signal can be used for gesture classification [284], foot gesture recognition [285], silent speech recognition [286], human–machine interfaces [287], clinical evaluations of muscle functions [288], etc. Epidermal electronic systems (EES) can form an intimate conformal contact with the skin surface through the action of van der Waals adhesion alone and is therefore an important method for measuring sEMG. Jeong et al. established a set of guidelines in materials, mechanics and geometric designs for EES configured to measure sEMG signal. During mechanical deformation of the skin, the EES demonstrated a higher SNR, by comparison to conventional electrodes [287]. Ramírez et al. proposed a wearable piezoresistive sensor composed of Palladium nano-islands on a single-layer graphene for measuring swallowing activity after radiotherapy for head and neck cancer. They combined these sensors with traditional sEMG and machine learning algorithms to achieve real-time monitoring and distinguish the signals generated by coughing, head-turning, and swallowing pills of different concentrations [289]. Ameri et al. reported a sub-micrometer thick, multimodal graphene electronic tattoo (GET) sensor with a total thickness of 463 ± 30 nm, optical transparency of about 85%, and stretchability of more than 40%. Since conformal contact increases the effective contact area, GET–skin interface impedance was on par with Ag/AgCl gel electrodes and essential for a high SNR in sEMG measurements. The open-mesh structure made the GET breathable and its stiffness negligible [290]. Jiang et al. developed a molecular engineering strategy based on a topological supramolecular network and fabricated a 64-channel microelectrode array that can record high-density spatiotemporal dynamics sEMG due to the low impedance of PEDOT:PSS and the low modulus of the entire electrode array [291]. Xu et al. presented a platform, where four transcutaneous electrical stimulation electrodes cointegrate on a common substrate with EMG sensor. Through geometry design, they simultaneously implemented the stimulation and measurement of EMG signals in a compact area of skin [288]. Choi et al. also integrated the stimulating and recording electrodes made of biocompatible Ag–Au core–sheath nanowires composite into one wearable device [166].

EOG signal reflects changes in the potential difference between the cornea and the fundus and is closely related to eye movement with a high SNR. Zheng et al. designed a four-electrode method based on forehead EOG for continuous alertness estimation, and it can be used in the actual driving environment [292]. EOG can also be used for HMI [293]. Electroretinogram (ERG) is also an electrical signal, which generated on the surface of the cornea by various neurons and non-neuronal cells in the retina in response to light stimulation. ERG can be used to assess the functional integrity of the retina. Wei et al. have produced a hydrogel contact lens to superimpose computer-generated visual information in the real world, providing instant and hands-free access to the information. They performed a full-field ERG recording on rabbits to prove that the device is suitable for daily wearing [294].

At present, electrodes are still a key tool for noninvasive wearable health monitoring devices to obtain electrophysiological signals, and many researchers are dedicating to optimizing electrodes. On one hand, reducing motion artifacts and improving skin compatibility are important factors to consider [295]. On the other hand, the latest research progress of the electrode mainly focuses on the innovations in material selection, structure, and process flow simplification to optimize electrode performance. In terms of materials, the electrode performance is optimized mainly by adding highly conductive materials or coating a highly conductive layer, such as adding PEDOT:PSS [296, 297], Ag ink [298, 299], AgNWs [88, 300], graphene [289], GO [301, 302], rGO [297, 303], Au [304, 305], and carbon nanofillers [306]. In addition, it is also important to choose suitable, soft, stretchable, and biocompatible substrates or polymer materials, such as PET [300, 307], PDMS [304, 308], and e-textiles [296, 301]. Some typical parameters of soft electrodes are listed in Table 3.

**Table 3:** Characteristics of different electrodes

Electrode materials	Signal	Type	Process	Pattern	Advantage	References
Modified PEDOT:PSS	ECG	Textile electrodes	Dip Coating	Solid circle	More than 50 washing cycles	[296]
PET, Ag Ink	ECG, EMG, EEG	Microneedle array electrodes	Laser-direct writing, Magneto-rheological drawing lithography	Serpentine	Low electrode–skin interface impedance	[298]
Graphene/Palladium Nanoislands/PMMA/PI Tape	sEMG	Piezoresistive sensor	Thermal evaporation, Sputtering, Spin coating	Dog-bone	Monitor swallowing function	[289]
rGO	EOG	E-Textiles	Chemically reduce	–	Capture EOG patterns	[301]
Ag Ink or PEDOT:PSS, PET	ECG	Textile	Screen printing	Concentric ring	High spatial resolution	[299]
rGO nanoplatelets, PET	ECG	Skin patches	Photolithography, Reactive-ion etching, Spray coating	Hexagonally symmetrical microchannels, convex cup	Greater adhesion on wet skin	[307]
AgNWs, GO, PET	ECG	Hybrid electrode	Screen printing	–	High transparency, good for mass production	[300]
AgNWs/PDMS	ECG	Composite electrode	Screen printing	Bipolar or Tripolar concentric ring	Reject powerline interference efficiently	[308]
Aproanthocyanins/rGO/PVA	ECG, EMG	Hydrogel electrode	Beak	–	Rapid self-healing, more accurate and stable detection	[303]
PMPC-Au/PDMS	ECG, EMG	On-skin electrode	Thermal evaporation, Surface-initiated atom transfer radical polymerization	Polymer brush	Clean the epidermal surface lipids by simple water rinsing	[304]
PEDOT:PSS-rGO NPs	ECG	Textile electrode	Coated knitted textile	Same as the commercial gel electrode	Combination of exhaust dyeing, Improved the washing stability and degradation of the textile electrodes significantly	[297]
Carbon Nanofillers /PDMS	ECG	On-skin electrode	Deep reactive-ion etching, Photolithography	Mushroom structure	Self-cleaning, Strong adhesion to human skin	[306]

**Fig. 14:**
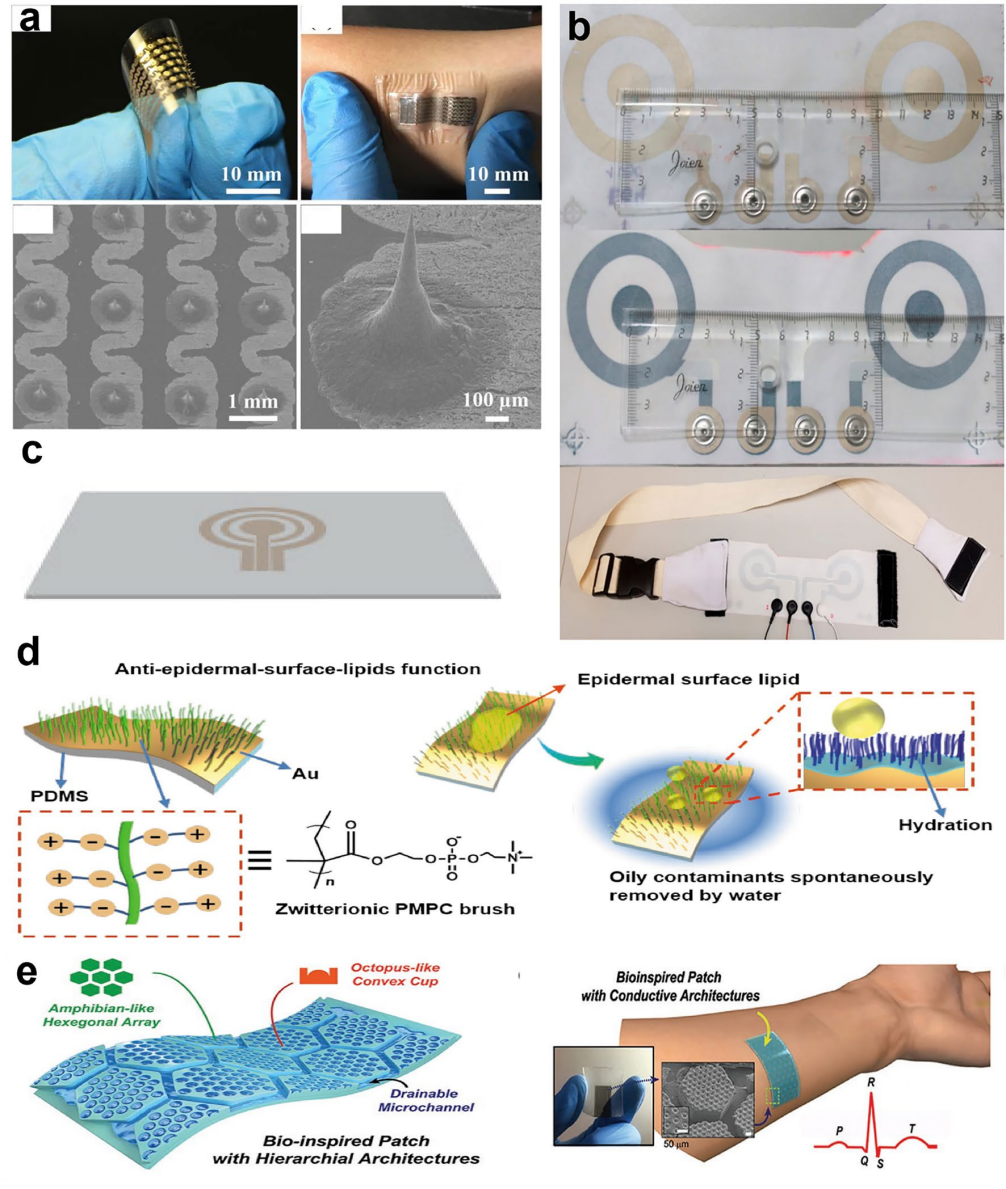
Typical electrodes with conductive patterns. **a** Image of the soft MAE. Reproduced with permission [298]. Copyright (2018), MDPI. **b** Schematic diagram of concentric ring electrodes (CREs) and CRE integrated with an adjustable belt. Reproduced with permission [299]. Copyright (2018), MDPI. **c** AgNWs paste screen printed on a glass substrate with a shape of the tripolar concentric ring. Reproduced with permission [308]. Copyright (2019), Institute of Electrical and Electronics Engineers. **d** Configuration of the designed electrode (left) and the water-enabled oil-cleaning effect (right). Reproduced with permission [304]. Copyright (2020), Wiley–VCH. **e** A skin patch inspired by the microchannel network of the tree frog toe pad and the convex cup on the octopus’s sucker. Reproduced with permission [307]. Copyright (2020), Wiley–VCH

With regards to structure, the current trend in electrodes design is shifting from a 2D structure to a 3D structure. For example, Ren et al. demonstrated that the soft serpentine-shape microneedle array electrodes (MAEs) can reduce induced strain and prevent the conductive pattern from broken (Fig. 14a) [298]. An electrode designed into a bipolar concentric shape (Fig. 14b) [299] or a dual tripolar concentric ring (Fig. 14c) [308] can obtain high-fidelity ECG signals. As shown in Fig. 14d, He et al. fabricated the detecting patch into a brush structure to reduce the influence of skin surface hair on the monitoring results [304]. To overcome the poor adhesion of electrodes in sweating or underwater environments, Kim et al. mimicked the microchannel network of the tree frog toe pad and the convex cup structure on the octopus sucker and designed similar structure to improve the adhesion of the electrode in a humid environment [307]. Similarly, Kim et al. designed a mushroom-shaped micropillar surface as the gecko to enhance the adhesion of the electrode (Fig. 14d) [306]. Yang et al. utilized the interlocking structure formed between interfacial polymerized conductive PPy and silk fibroin (SF) gel to ensure the SF biocomposite electrode still has good shape retention and adhesion even under sweating conditions [309].

In addition to improving the conductivity and reducing the electrode–skin interface impedance to obtain a high SNR, some studies also discussed other aspects of electrode performance optimization, for example the washability of the electrodes. Textile electrodes made of cotton, polyamide, and polyester coated with PEDOT:PSS can monitor ECG signals after washing 50 times [296]. Compared with pure PEDOT:PSS and rGO, PEDOT:PSS-rGO compound has better washing stability [297]. By adding super hydrophilic zwitterionic groups, epidermal lipids can also be removed by washing with water (Fig. 14e) [304]. The high-aspect-ratio micropillars with spatula tips make the electrode surface superhydrophobic [306]. Shao et al. prepared waterproof electrodes coated with dopamine-containing polymer, which can be used for real-time ECG signal monitoring underwater [310].

### Brief Summary

Up to now, many physiological signals (such as pulse, respiration, human motion, IOP, phonation, tactile sensation, heart sounds, ECG, EEG, EMG, EOG, etc.) can be detected by the contact methods. During the monitoring process, the tight interface between soft electronics and skin is important to the high signal quality. As the most devices discussed above, the sensitive material is protected by the soft package for example PDMS and Ecoflex, rather than come into contact with skin directly. Therefore, transferring the soft sensitive material to the soft package is a significant process. In addition, to realize the tight interface, the soft package material should have similar elastic modulus and Poisson's ratio with skin, which can avoid the relative motion and noise. The thickness of total device consists of soft sensitive material and package should be as thin as possible. Taking the advantage of wrinkle structure of skin, the device with the thickness less than 10 μm can grasp skin tightly. The airtight package will greatly influence the skin metabolism and wearing experience, which limits the wide application of the soft electronics. The nanomesh and other fiber package is a good solution.

## Soft Electronics Assisted by Machine Learning Algorithm

Machine learning algorithms are multidisciplinary mathematical model mapping methods, which specially study how computers simulate or realize human learning behavior, so as to acquire new knowledge or pattern, reorganize the existing knowledge structure and constantly improve its own performance. Nowadays, machine learning can help human beings in many fields. The machine learning algorithm can be divided by some methods, for example supervised learning, unsupervised learning, and reinforcement learning, or classification and regression. In 2011, Kim et al. in Rogers’ group demonstrated the epidermal electronics [311]. They used the algorithms to classify the EMG signals.

Supervised learning requires training data with labels. The algorithm is told the correct answers during the learning process. After learning for enough time and data, the algorithm then deals with the test set like an examination. Most of the algorithm used in the soft electronics is the supervised learning. Unsupervised learning does not need labels, it only has input data, and the algorithm can only find the law in the data by itself. The reinforcement learning is a kind of machine learning algorithm inspired by behaviorist psychology, concerned with how software agents ought to take actions in an environment so as to maximize some notion of cumulative reward. Through this optimal strategy, intelligent physical fitness actively adapts to the environment to maximize future rewards. The reinforcement learning has not been widely used in the soft device, which will not discussed carefully in this review.

Classification and regression model both predict the output according to the input. If the output of the machine learning model is the class belongs a discrete value, such as an integer value, the model was called a classification model, for example, forecasting the meaning of different gesture and the disease type represented by the physiological signal. If the output of the machine learning model is the value of an object, the model with continuous output values is called a regression model. For example, the algorithm predicts the current body temperature through the previous temperature and the pressure values through the skin appearance. The results of classification model are certain, which only has two conditions right and wrong. Regression model gives an approximate prediction of the real value, which is uncertain. The closer the predicted value to the real value, the smaller the error. Then the model is considered to be a good regression model. In this review, due to the classification algorithms were used in most of the research on soft electronics, the classification model was mainly discussed. In this section, many classic machine learning algorithms will be first discussed. Due to the rapid development and its tight combination with soft electronics, the neural network will be introduced carefully. Some parameters of algorithm-assisted soft electronics are illustrated in Table 4.

**Table 4:** Typical machine learning-assisted soft electronics

Material	Device	Wearing position	Classification signals	Algorithm	Data size	Dataset scale	Accuracy	References
Ag ink	Soft electrode array	Arm	sEMG	PCA	16 × 4 channel	N.A	13 classes 97.12%, 21 classes 92.87%	[312]
Stainless steel/Rubber/Polyester/PDMS	Sensor array	Hand	Hand gesture	PCA and SVM	5 channels	660	98.63%	[31]
Eastomer/IC chip/PI/Cu connection/PI	Seismocardiography (SCG), EMG, and ECG sensor	Neck	EMG for speech monitoring	LDA	100 ms window	80	90.00%	[27]
Flexible IGZO TFT processor and OFET sensor	e-nose	N.A	VOCs	GNB, SVM, MLP, DT, kNN	8 channels	N.A	92% by flexible processor	[313]
Silicone elastomer/IC chip/Cu connection/PI	Temperature sensor, accelerometers	Suprasternal notch	Cough of COVID-19 patients	SVM	0.5 s window	1943	> 90%	[348]
LIG/medical PU tape	Electrodes and Pressure sensor	Face	EOG	SVM	150	N.A	92.60%	[314]
PDMS/PI/Cu	EEG monitoring sensors	Head	SSVEP with different frequency	SVM, CNN	2 channels, window sizes of 128, 192, 256, 384 and 512 data points	2700	94.54%	[32]
Ag/AgCl	EMG sensor	Arm	Hand gesture	SVM	64 channels	N.A	96.64%	[349]
Fiber-based yarn/piezoelectric polymer	Pressure sensor	Cushion	Pressure distribution	DTW	16 × 16 channels	700	85.90%	[315]
PDMS/PI/Cu	ECG, PPG, SCG, gross acceleration sensor	Sternum	Apneas and hypopneas, Sleep stage	Morse WT, R-CNN, FFNN	4 channels	990	Apneas and hypopneas 100%, Sleep stage 82.4%	[350]
MEMs microphone and flexible circuit	SWS	Chest and back	Heart sounds	WT and CNN	8 s window	8000	94.78%	[29]
AgNWs, Regenerated edge-decorated graphene	Wearable transient epidermal electronic system	Head, arm, and chest	ECG, EMG, EOG, and Electro-bacterial-graph	WT and ANN	4 channels	160	96.90%	[351]
Silicone elastomer/IC chip/Cu connection/PI	Temperature sensor, accelerometers	Suprasternal notch	Cough of COVID-19 patients	CWT, CNN (ResNet), STFT	60 × 666 × 1	8446	> 85%	[352]
POSFETs	Capacitive-piezoelectric tandem sensors	UR5 robot arm	Naturalistic texture	Gabor WT and SNN	N.A	200	99.45%	[345]
PAMAC-L hydrogel	Strain sensor and pressure sensor	Finger	Handwriting	Machine Learning toolbox of MATLAB: DT	About 3 s windows	Paper model: around 1500; Air model: around 1000	Paper model: 88.8%; Air model 93.1%	[317]
Graphite sheets and Cu foil	ECG, strain, and galvanic skin response sensors	Chest and hand	Mental fatigue levels	DT, SVM, and kNN	1 min per data	540 min per subject	89%	[353]
BaTiO_3_/rGO/PDMS	Tactile sensor	N.A	Surface texture	MATLAB Toolbox	1 × 1000	1648	98.80%	[260]
AlN/Mo/SiO_2_/PDMS	Strain sensor	Face	Facial movements	kNN and DTW	2 × 2 channels	324	86.80%	[316]
AgNPs	EOG sensor	Face	Ocular vergence	kNN, SVM	3 channels	N.A	91.00%	[28]
Double-layer MXene-PDMS-PE (MPP) films	Pressure sensor	Ear	Sound	K-means	1.5 s	280	95.00%	[26]
Shelf knitted glove	Resistive and fluidic pressure sensors	Glove	Nodal pressure, Object stiffness, Temperature, Conductibility	MLP, LSTM	16 channels	N.A	Stiffness 99.38%, Temperature error 0.92 °C, Conductibility 84.7%, Object 99.69%, Handwritting 86.68%	[34]
Microfluidic channel, ionic liquid, conductive fabric layer	Optoelectronics, microfluidics, piezoresistivity sensor	Hand and arm	Stretching, Bending, Local compression	FNN	3 channels	N.A	> 95%	[354]
PDMS-MPU_0.4_-IU_0.6_/MIBK/Ag flakes Al/TiO_2_/Al PDMS/PI/Ag/ZnO NPs/QDs/ CBP/MoO_x_)/Ag/SiO_2_	A stretchable capacitive pressure sensor, an RRAM, and a QLED	N.A	Pressure distribution	FNN	5 × 5	160	100% and the joint weight realized by RRAM array	[346]
Au/Cr/PET	EMG sensor	Arm	Hand gesture	FNN by MATLAB	4 × 4 channels	N.A	97.40%	[338]
PAAm/PAA-Fe^3+^ hydrogels	Pressure sensor	Under foot	Pressure distribution	FNN	16 × 16	74,257	Regression model, ICC = 0.61	[355]
Piezoresistive fibers	Pressure sensor	Glove, sock, sleeve, and vest	Pressure distribution	CNN	Sock 8 × 24 Vest 32 × 32	> 1,000,000	Glove 83% Sock 95.8% Sleeve 90.6% Vest 99.66%	[33]
Textile-based TENGs	Pressure sensor	Sock	Gait	CNN	3 × 100	325	93.54%	[356]
Graphene/Ag /PI	All-printed EMG sensor and circuit	Arm and leg	EMG and acceleration	CNN	1 × 128	N.A	Arm 98.5%, leg 97.3%	[357]
Soft electrode/Ecoflex	Soft ECG and acceleration circuit system	Chest	ECG, 3-axis angular orientation, and 3-axis acceleration	CNN	4 channels	N.A	99.30%	[358]
Soft microneedle electrodes/Cr/Au	EEG sensor	Head	Event-related desynchronization and event-related synchronization	CNN	1000 × 6	2240	92.33%	[343]
Triaxial accelerometer (WHS-3 SENSOR), stretchable strain sensor (C-STRETCH)	An accelerometer and a strain sensor	Neck and chest	Acceleration and strian	CNN	4 channels 10 s window	1166	Healthy volunteers 92%, the patients with cough 96%	[359]
LSG/PU nanomesh	Pressure sensor	Finger	Tactile sensor	CNN	1 × 250	303	88.00%	[360]
ITO/PU nanomesh	Strain sensor	Throat	Artificial throat	CNN	1 × 150	1090	86.50%	[361]
LSG	Strain and ECG sensor	Chest	Arrhythmia	CNN	6 s window	N.A	92.65%	[362]
Au nanomesh, Au/PU nanomesh	Strain, ECG, and EMG sensor	Throat	Artificial throat	CNN	2 channels 1 × 20 and 1 × 400	93 for test set	98.90%	[182]
Seaweed/Gelatin/Agar hydrogel	Triboelectric sensor	Body area	Motion pattern	CNN	11 × 500	360	100.00%	[363]
PAAm gel	Pressure sensor	Contactless	3D object recognition (electroreceptor)	CNN	21 × 21	196	97.00%	[364]
Digital fibers (W wires/PMMA/PC) and electrical pads	Temperature sensor and digital chip	Cloth	Motion state of wearer	CNN and In situ calculation	N.A	2564	96.40%	[347]
Graphene/Ag/PI	All-printed EMG sensor and circuit	Arm	Hand gesture	CNN and kNN	3 channels	N.A	99.00%	[30]
GelSight sensor	Optical-based tactile sensors	Robot gripper squeezes	Object hardness	CNN-LSTM	960 × 720	7000 videos	R^2^ = 0.9564, RMSE = 5.18	[365]
LSG/PU nanomesh	Strain, EEG, EOG, and ECG sensor	Head	Attention level	FFT and CNN	1 × 256 and 1 × 512	2612	48.70%	[183]
AgNPs/PI/PDMS	EMG sensor	Arm	Hand gesture	LSTM and transfer learning	16 channels	4680	96.20%	[344]
MXene-PU nanomesh	12-lead ECG sensor	Chest	Arrhythmia	CNN & LSTM	8 channels	27,654	> 99.3%	[366]

### Classic Machine Learning Algorithms

Classic machine learning methods, such as SVM and K-mean, usually have smaller computation scale, which usually corporate with data dimension reduction or feature extraction methods such as PCA, LDA, Fourier transform (FT), and WT. The classic machine learning methods are more suitable for those with obvious features and few classification types. Therefore, the dimension reduction and classic machine learning classification algorithms will be carefully discussed.

#### Dimension Reduction Algorithms

##### Principal Component Analysis

**Fig. 15:**
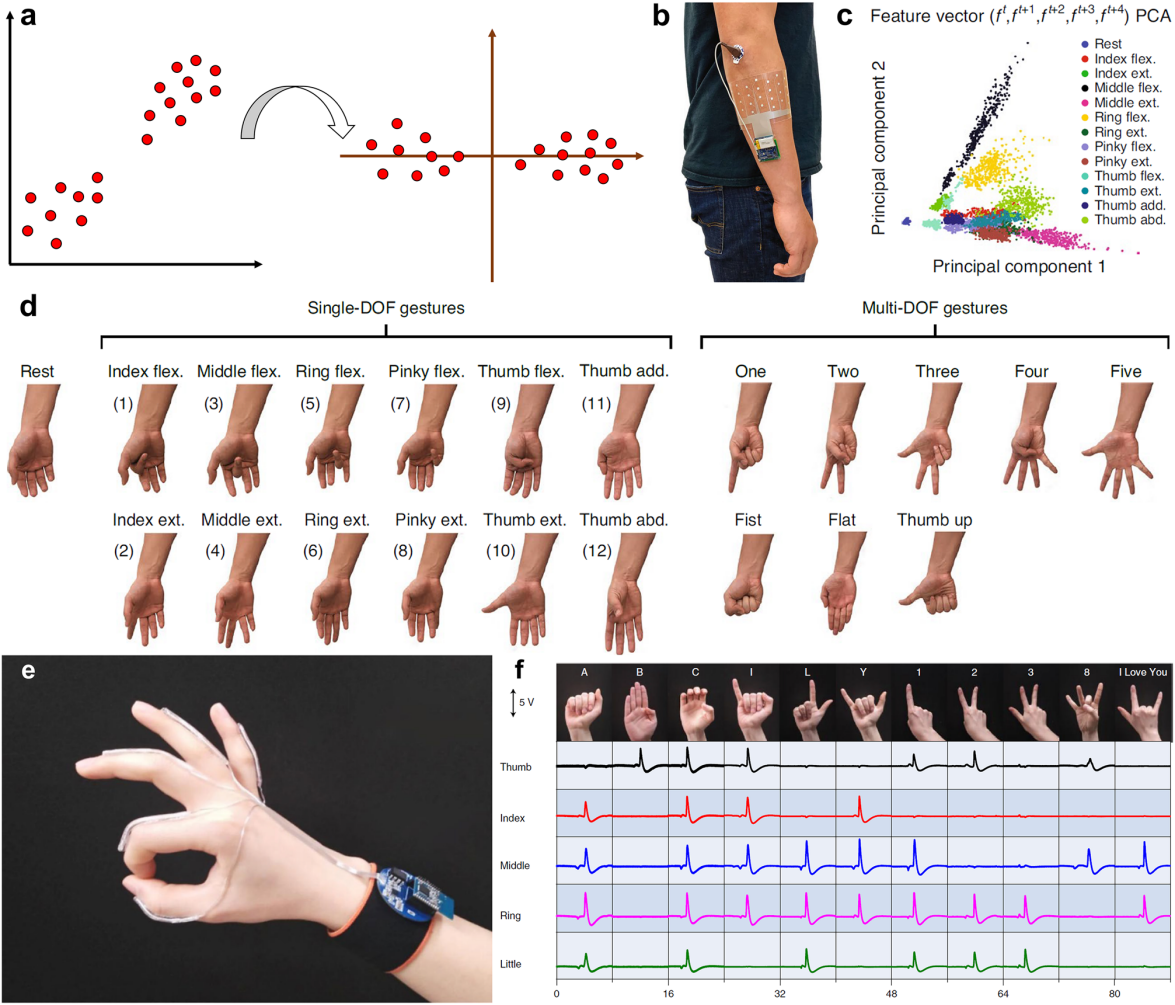
Signals detected by the soft system and processed by PCA. **a** Schematic diagram of PCA. **b** 64 channels sEMG detected system on the forearm of a participant. **c** Single-DOF gesture subset. The multi-DOF gesture subset. **d** PCA for all classification windows from five trials of 13 single-degrees of freedom gestures. The top two principal components are plotted for features. Reproduced with permission [312]. Copyright (2021), Springer Nature. **e** Photograph of YSSA and the wireless PCB attached on a subject’s hand. **f** Photographs of the sign language hand gestures according to ASL and the corresponding voltage profiles generated by YSSA as recognition pattern. Reproduced with permission [31]. Copyright (2020), Springer Nature

PCA is an unsupervised dimension reduction method, whose main function is compression and simplification. When studying multivariable problems, too many variables will increase the complexity of the work. The goal is to obtain as much effective information as possible by analyzing as few variables as possible. In many cases, there is a certain correlation between variables, that is, it can be considered that there is overlap between the information reflecting this topic. PCA is a multivariate statistical method which can analyze the correlation between multiple variables. First, it is assumed that the data follows the Gaussian distribution. Then, how to reveal the internal structure of the total variables through a few principal components is studied. A group of variables that may have correlation is converted into a group of linearly unrelated variables through orthogonal transformation. In other words, the *n*-dimensional feature is mapped to the *k*-dimensional feature, which is a new orthogonal feature, also known as the principal component. After the PCA, a group of orthogonal coordinate axes originated from the original space are found. Among them, the first new coordinate axis is the direction with the largest variance in the original data, the second new coordinate axis is the one with the largest variance in the plane orthogonal to the first coordinate axis, and the third axis is the one with the largest variance in the plane orthogonal to the first and second axes (Fig. 15a).

Moin et al. reported a wearable and high-density sEMG biosensing system that uses PCA dimension reduction for hand gesture classification [312]. 64 Ag electrodes array were fabricated on the PET substrate by screen printing and connected with a miniaturized printed circuit board (PCB) that includes complex sensing, processing and telemetry components. An ECG Ag/AgCl electrode attached on the elbow was applied as reference. The total weight of the sEMG system is only 26 g (Fig. 15b). Then, the system could be used to monitor muscle activity, which can also reflect the hand gesture. To process the hyperdimensional data detected by the sEMG system, PCA was performed on the classification windows consisting of five feature vectors, as well as on projected spatiotemporal hypervectors from trials of the single-degrees of freedom (DOF) gestures. The top two principal components show general clustering of different gestures (Fig. 15c). The system can classify 13 DOF hand gestures with 97.12% accuracy for two participants when training with a single trial per gesture. A high accuracy (92.87%) is preserved on expanding to 21 gestures (combined with the multi-DOF gesture) (Fig. 15d).

Zhou et al. realized a wearable sign-to-speech translation system based on yarn-based stretchable sensor arrays (YSSAs) and a wireless PCB (Fig. 15e) [31]. Assisted by machine learning, the system can accurately translate the hand gestures into speech. The yarn unit is composed of a conductive yarn coiled around a rubber microfiber. Then, the entire body is sheathed with a PDMS sleeve. The yarn can be self-powered with the sensitivity of 2.47 V. After attached on the hand, the system can translate the hand gesture assisted by PCA and SVM algorithm (Fig. 15f). A multi-class SVM algorithm, which will be discussed later, is applied to classify hand gesture patterns by using the extracted features with PCA dimensionality reduction. By analyzing 660 hand gesture recognition patterns, the recognition rate can be up to 98.63% and a real-time translation can be realized with the recognition time less than 1 s.

##### Linear Discriminant Analysis

**Fig. 16:**
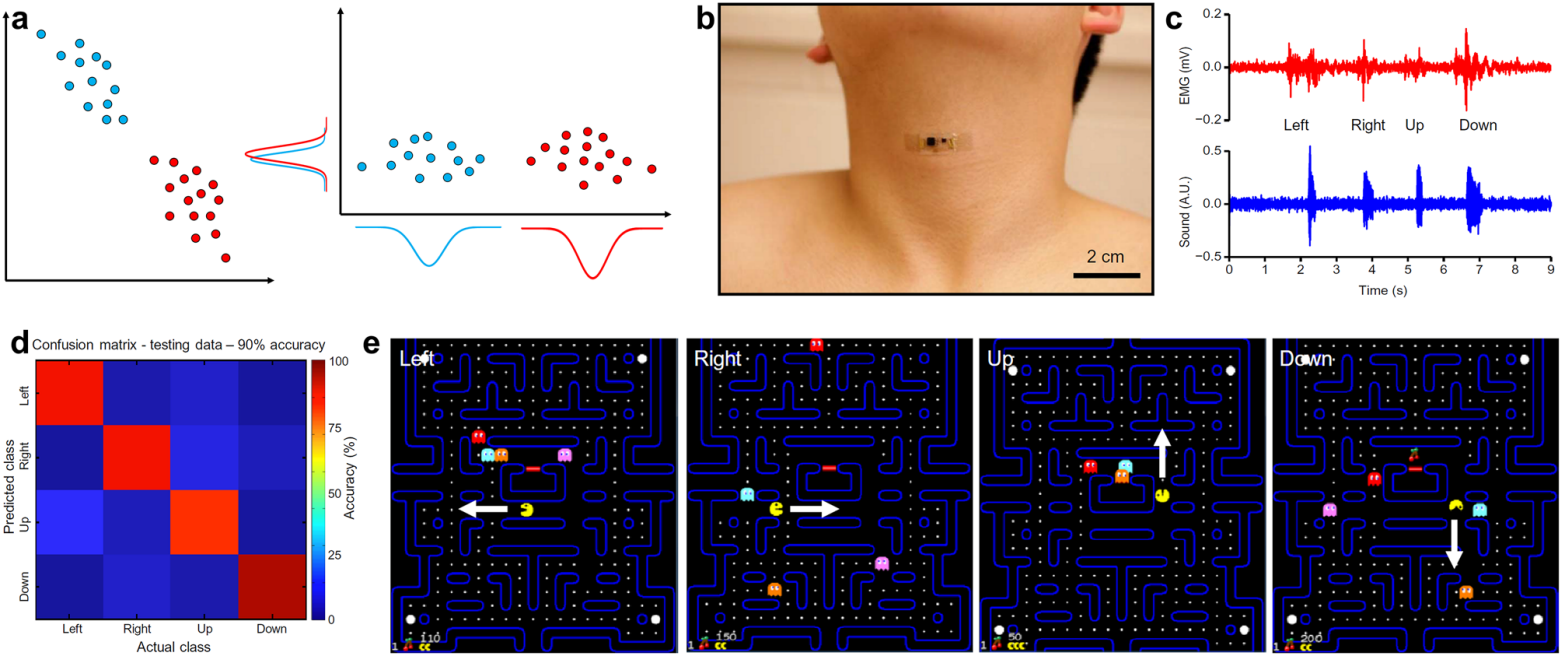
Signals detected by the soft system and processed by LDA. **a** Schematic diagram of LDA. **b** Photograph of an epidermal mechanoacoustic sensing electronics attached on the vocal cords. **c** EMG (top) signals and vocal vibrational (bottom) signals detected simultaneously from the neck. **d** Confusion matrix of the result of the speech classification. **e** Demonstration of speech recognition and classification in a Pac-Man game, which can realize left, right, up, and down instruction. Reproduced with permission [27]. Copyright (2016), American Association for the Advancement of Science

When processing a given training sample set, LDA projects the samples onto a straight line, which makes the projection points of similar samples as close as possible and the projection points of different samples as far as possible. In a word, the intra class variance is the smallest and the inter class variance is the largest after projection (Fig. 16a). When used as classification algorithm, data is projected onto the straight line, and then the category of the new sample is determined according to the position of the projection point. PCA and LDA have some similarities. For example, both PCA and LDA can be used to reduce the dimension of data. Both of them use the matrix eigen decomposition in dimension reduction and assume that the data follows Gaussian distribution. However, compared with PCA, LDA is a supervised dimension reduction algorithm. In PCA, the algorithm does not consider the label of data. LDA selects the projection direction with the best classification performance, while PCA only selects the direction with the largest variance of the sample point projection.

Liu et al. reported a mechanoacoustic monitoring platform for multimodal operation, which can record EMG and sound from the skin (Fig. 16b) [27]. The mechanoacoustic–electrophysiological sensing platform consists of circuit interconnects, accelerometer, amplifiers, resistors, capacitors, small-scale chip, a pair of electrophysiological measurement electrodes (Au), etc. All components are encapsulated above and below by an ultralow-modulus elastomeric core (Ecoflex, Smooth-On), which can realize the robustness of adhesion to the skin. The intimate contact between the sensors and the skin renders the signal unaffected by ambient acoustic noise. With appropriate placement, the platform can simultaneously capture both EMG signals from articulator muscle groups and acoustic vibrations from the vocal cords (Fig. 16c). Combining the platform and a standard microphone, speech recognition can be realized. The signals were averaged and reduced in dimensionality by PCA to form a feature vector. Then, the feature vector was finally classified using LDA (Fig. 16d). Finally, the system can be used in real time to play a Pac-Man game and express four commands: “left,” “right,” “up,” and “down.” (Fig. 16e).

##### Fourier Transform and Wavelet Transforms

FT decomposes the signal into the superposition of a series of trigonometric functions of different frequencies for analysis. However, for non-steady state signals, FT cannot reflect the frequency change under different time. When FT is applied, each frequency component calculated corresponds to the time range of the whole signal, which makes the time information of the original signal lost, and the change of frequency with time cannot be analyzed, and the sudden change occurring at a certain time cannot be located. In order to overcome the shortcomings of FT, the whole time-domain signal is decomposed into numerous smaller processes of equal length (windowed). Each process is approximately stable, and then FT is used to obtain the frequency spectrum, which called short-time Fourier transform (STFT). The narrow window has high time resolution and low frequency resolution, and the wide window has low time resolution and high frequency resolution. Therefore, for time-varying non-steady signals, the high-frequency part is suitable to be analyzed by small windows, and the low-frequency part is suitable to be analyzed by large windows. However, during one time of STFT, the width of the window is fixed. Therefore, STFT also has its limitations.

**Fig. 17:**
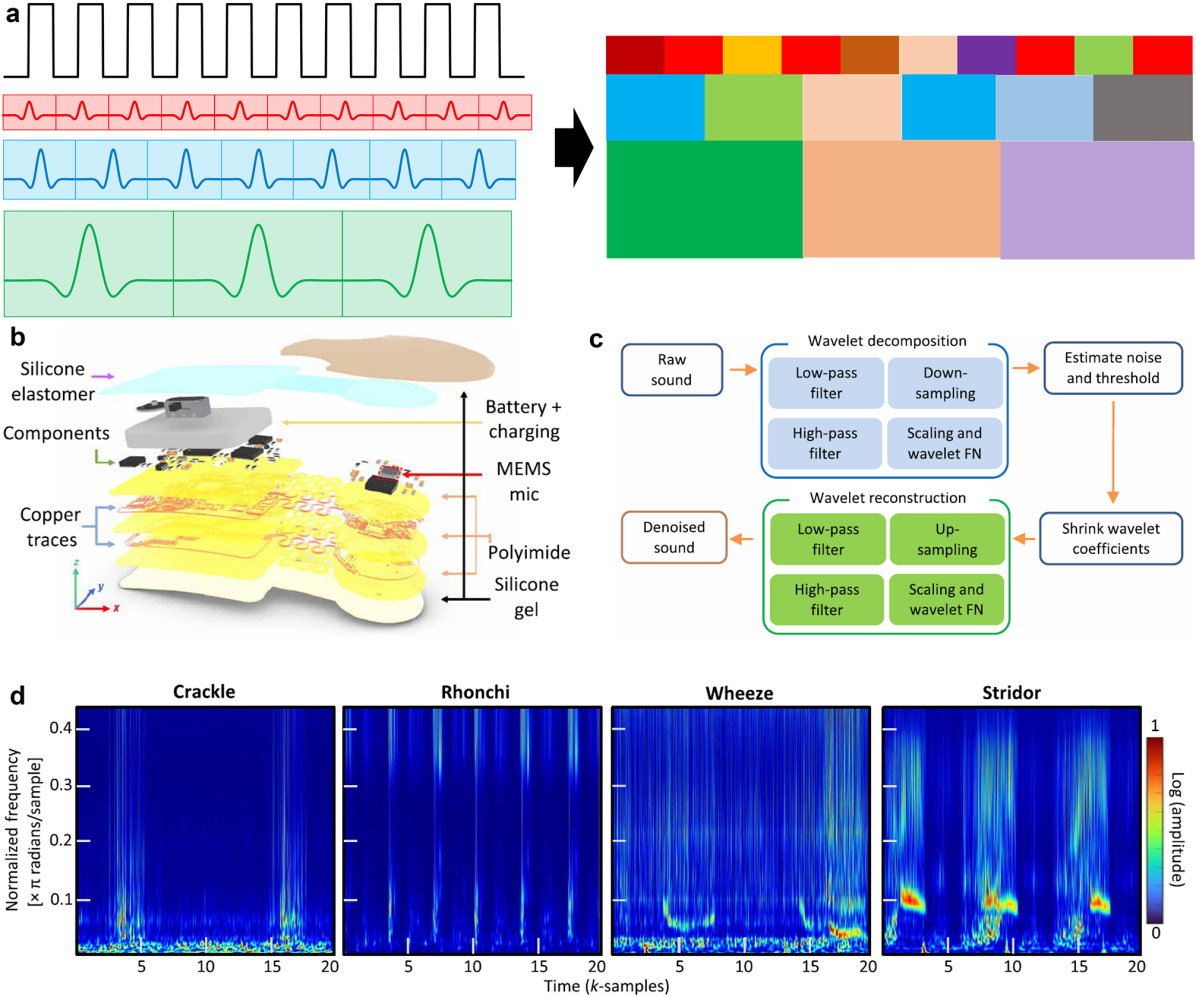
Signals detected by the soft system and processed by WT. **a** Schematic diagram of WT. **b** Exploded view of the SWS with multiple layers of deposited materials. **c** Schematic illustration of the flowchart of the wavelet denoising algorithm to realize decomposition and recomposition of collected sounds. **d** Scalogram of crackle, rhonchi, wheeze, and stridor data in sample series versus normalized frequency with density after the wavelet denoise for each sample. Reproduced with permission [29]. Copyright (2022), American Association for the Advancement of Science

WT can be regarded as the base transform of FT, which transforms the infinitely long trigonometric function base of FT into a finite long attenuated wavelet base. WT decomposes the signal into a series of wavelet functions with different scales and different times, and these wavelet functions are obtained from a mother wavelet through translation and scaling. The energy of wavelet base is limited and concentrated near a certain point. In addition, the integral value of wavelet base is zero. Therefore, WT can be used for time–frequency analysis to obtain the time–frequency spectrum of the signal. Wavelet can also be considered as a band-pass filter, which only allows signals whose frequency is close to the center frequency of wavelet after scaling (Fig. 17a).

Lee et al. reported a digital stethoscope using a soft wearable system as a quantitative disease diagnosis tool for various diseases [29]. The soft wearable stethoscope (SWS) system includes a microelectronic mechanical system (MEMs) microphone sensor, a soft thin film circuit wiring, a rechargeable battery, and a Bluetooth-low-energy (BLE) unit for wireless data transmission. Then, the system was packaged in an elastomeric enclosure with an inner silicone gel (4 kPa in Young’s modulus) (Fig. 17b). Compared with other substrates (3 M 2476P tape, 3 M Tegaderm tape, and 3 M Micropore tape), the silicone substrate can realize better device-skin contact, which can improve the SNR to 16 dB. WT was used to the noise filtering processes of heart and lung sound signals, which was crucial in the signal processing and classification because the microphone captures all sounds from the body and the surrounding (Fig. 17c). Then, each sample (Crackle, rhonchi, wheeze, stridor, and normal case, Fig. 17d) is clustered into 2-s packets and fed into CNN algorithm. The classification results show a high accuracy of 94.78%.

#### Classic Machine Learning Classification Algorithms

##### Gaussian Naive Bayes

Gaussian Bayes model refers to the assumption that the conditional probability of each feature dimension of the sample follows the Gaussian distribution. Then, the posterior probability of the new sample belonging to each category was calculated according to the Bayesian formula (Eq. 1). Finally, the category of the sample was obtained by maximizing the posterior probability.

1 $$P\left( {A|B} \right) = P\left( {B|A} \right) \times P\left( A \right)/P\left( B \right)$$

Naive Bayesian method is a simplification on the basis of Bayesian algorithm, where the distribution is assumed to be conditionally independent when the target value is given (Eq. 2).

2 $$P\left( {AB} \right) = P\left( A \right) \times P\left( B \right)$$

**Fig. 18:**
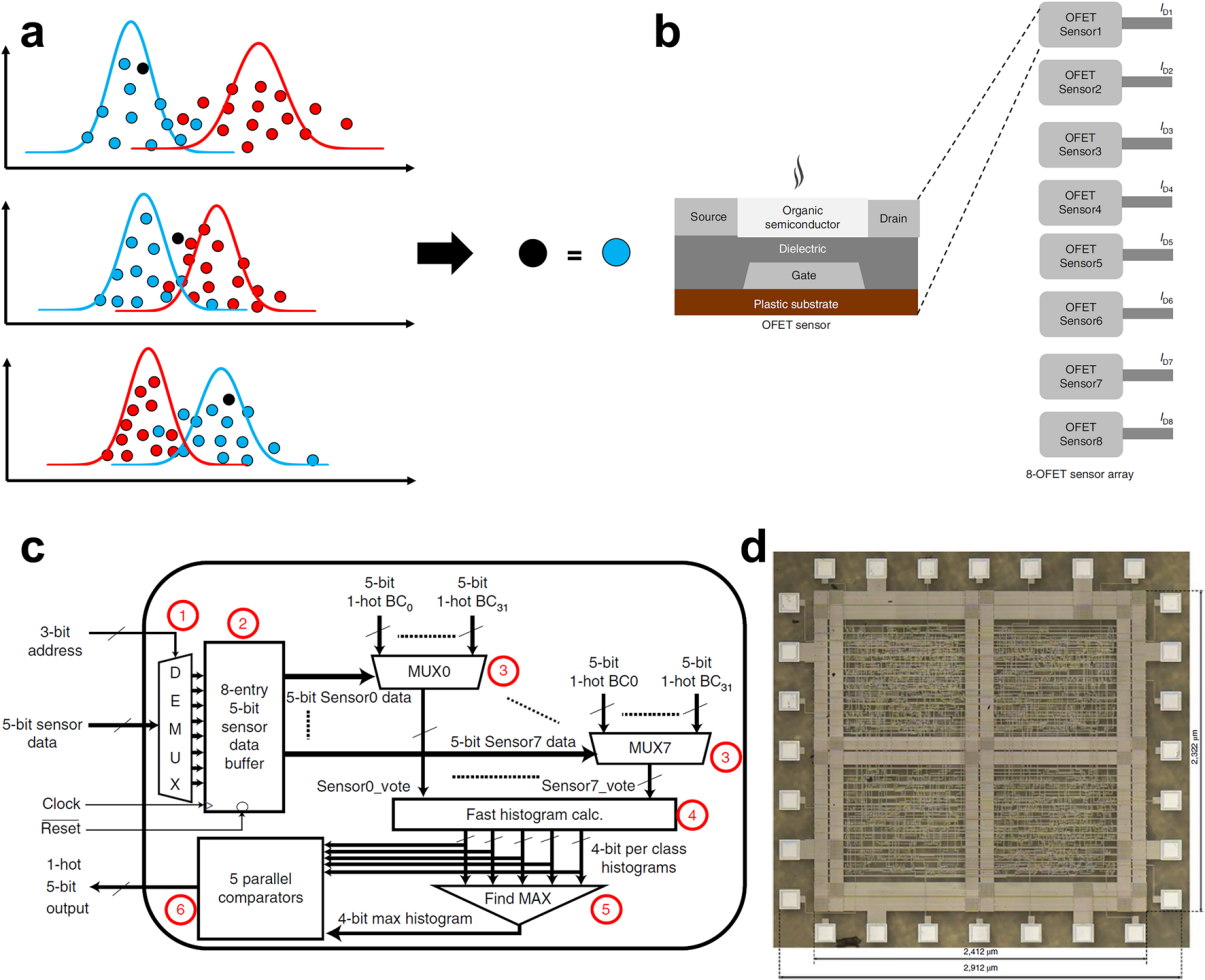
Signals detected by the soft system and processed by GNB based on soft chip. **a** Schematic diagram of GNB. **b** Schematic diagram of a single OFET sensor and an e-nose sensor array containing eight OFET sensors. **c** Microarchitecture of the UB-FVC inference stage. Five-bit sensor data are received serially and demultiplexed (block 1) into the sensor data buffer (block 2). Each feature is implemented as a multiplexor (blocks 3). Then, a fast histogram count calculation is performed by Block 4 for the eight BCs or votes. The highest histogram value is calculated through a comparator reduction tree (‘Find MAX’ block 5). Five parallel comparators (block 6) take the five histogram values and compare each one with the highest histogram value from block 5 to find the statistical mode. **d** Micrograph of the NFPE implementing the UB-FVC microarchitecture. Reproduced with permission [313]. Copyright (2020), Springer Nature

GNB assumes that each parameter satisfies Gaussian distribution and has independent ability to predict output variables. The probability of the dependent variable classified into each group was calculated, and the final classification is the combination of all parameters and assigned to the classification with higher probability (Fig. 18a).

Ozer et al. developed a soft processing engine named ‘natively soft processing engine’ (NFPE) that operates as a central processing unit (CPU), where machine learning algorithms can be implemented for an odor recognition application [313]. The NFPE is fabricated using 0.8-μm IGZO TFT technology, and contains 1024 logic gates. The *n*-type TFT circuits are made on a soft PI substrate with a minimum supply voltage of 3 V. A soft e-nose sensor array consisting of multiple organic field effect transistors (OFETs) (Fig. 18b). Each OFET sensor has an organic semiconductor channel that is sensitive and selective to volatile organic compounds (VOCs) in odor and can generate a current when exposed to odor. To develop ML hardware to classify odor, a number of standard ML algorithms, such as SVM, multilayer perceptron (MLP), DT, kNN, and GNB were investigated, and the GNB has the best performance with a prediction accuracy of 92%. Finally, the ‘univariate Bayes feature voting classifier’ (UB-FVC) was implemented in the NFPE for sweat odor classification (Fig. 18c, d).

##### Support Vector Machine

SVM is a generalized linear classifier that classifies data by supervised learning. The decision boundary is used to make the classification, which is the maximum margin hyperplane. When the data is linearly separable, the optimal classification hyperplane of two types of samples can be found in the original space. When the linearity is inseparable, the slack variable is added and the samples in the low dimension input space are mapped to the high dimensional space by using nonlinear mapping to make them linearly separable, so that the optimal classification hyperplane can be found in the new feature space. The tool to rise the dimension is kernel function.

**Fig. 19:**
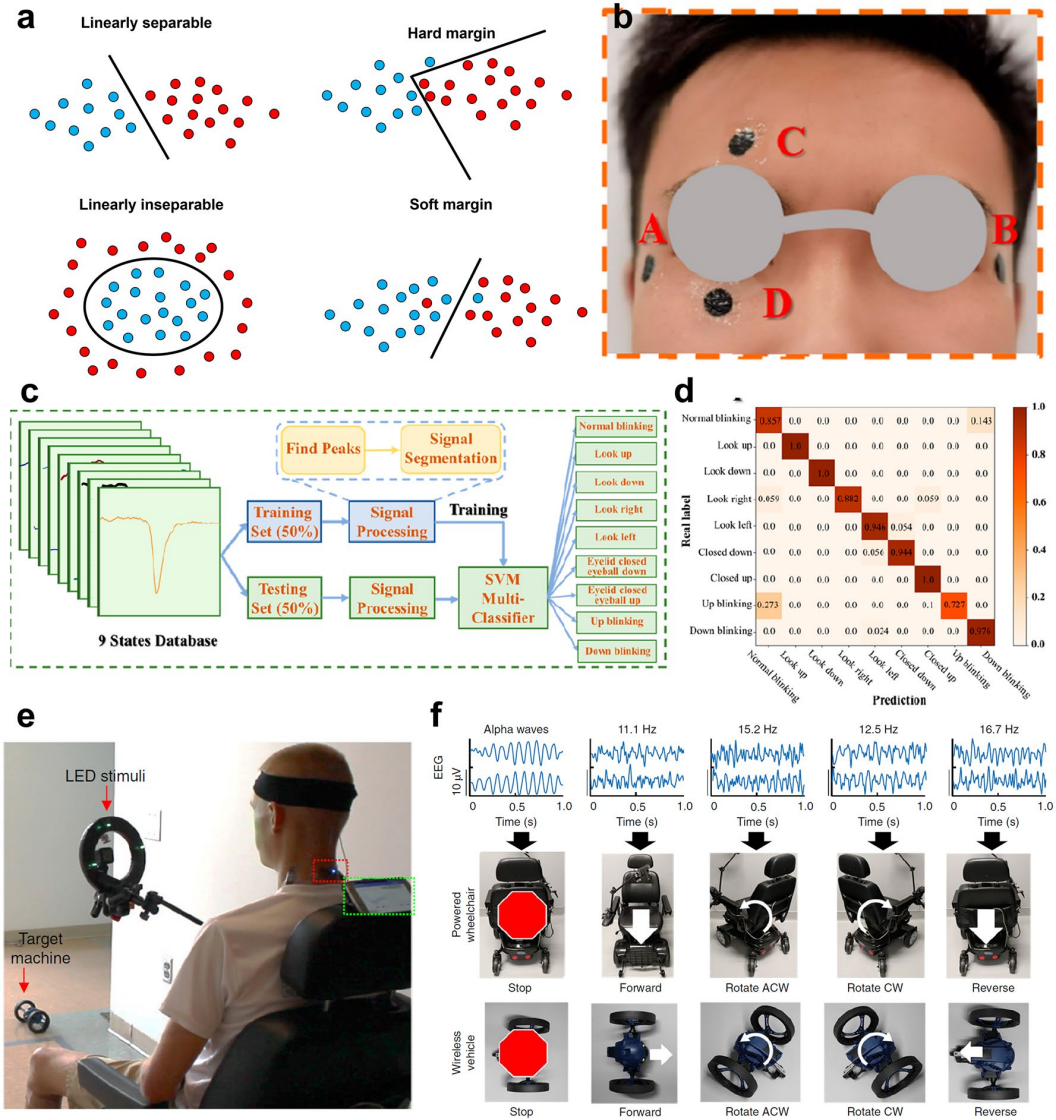
Signals detected by the soft system and processed by SVM. **a** Schematic diagram of SVM. **b** Position of honeycomb graphene electrodes attached around eyes of tester. **c** Flow chart of the SVM algorithm for eye movement classification. **d** Confusion matrix of SVM classification training recognition accuracy. Reproduced with permission [314]. Copyright (2022), American Chemical Society. **e** A tester seated in a powered wheelchair with the LED stimulus array in front of the tester. **f** EEG data recorded at each state, labeled as alpha rhythms, 11.1, 15.2, 12.5, and 16.7 Hz SSVEPs respectively, which corresponds to different commands. Target machines to be controlled by SSVEPs signals, including a wireless electric wheelchair with five classes (no action, forward, rotate anticlockwise, rotate clockwise, and reverse), and a wireless vehicle with the same commands as the wheelchair. Reproduced with permission [32]. Copyright (2019), Springer Nature

SVM can also be used for multi classification problems by combining many two classifiers to construct multiple-class classifier. During the training process, the samples of a certain class are classified into one class, and the remaining samples are classified into another class. Thus, K SVMs are constructed from the samples of K classes. The unknown samples are classified into the class with the largest classification function value (Fig. 19a).

As mentioned above, Zhou et al. developed a multi-class SVM algorithm to classify hand gesture patterns using the extracted features with PCA [31]. Then, each class of the acquired hand gesture recognition pattern is set as a classifier sample, and the other remaining samples are set as another classifier sample obtain a binary-class SVM classifier and then create a multi-class classifier.

Xu et al. proposed a collaborative interface including EOG and tactile perception for fast and accurate 3D HMI [314]. The EOG signals are mainly used for the 2D (*XY*-axis) interaction, and the tactile sensing is utilized for the *Z*-axis control in the 3D interaction. The EOG electrodes and tactile sensor are based on the honeycomb LIG. Patterned LIG produced on the commercial PI film was transferred to the medically nonsensitive PU film as the electrodes, which are attached around the eyes for monitoring nine different eye movements (Fig. 19b). The open eyelids, the left and right movements of the eyeball can make changes in EOG that reflect the potential difference between the retina and cornea. Nine eye movement signal sets were trained and verified by the lightweight SVM classification algorithm that can be easily implemented into wearable electronics (Fig. 19c). The confusion matrix results show that the average prediction accuracy of the algorithm is 92.6% (Fig. 19d). To realize 3D HMI, a 4 × 4 capacitive tactile sensor array was realized with the LIG as the electrodes, which can control the *Z*-axis, which can provide the simplest and most convenient interaction for people with mobility difficulties.

Mahmood et al. developed a fully portable, wireless, and soft scalp electronic system (referred to as ‘SKINTRONICS’), containing an ultrathin aerosol jet-printed skin electrode, three soft conductive polymer electrodes and a soft membrane circuit [32]. The EEG recording setup for two channels (O1–Oz and O2–Oz) incorporates an aerosol jet-printed skin-like electrode. Due to the extreme mechanical compliance and small form factor, SKINTRONICS exhibits a significant reduction of noise and electromagnetic interference, compared to the existing portable EEG systems with rigid electronic components. The system can realize real-time long-range wireless data acquisition and accurate classification of steady state visually evoked potentials (SSVEPs) with a high information transfer rates from only two recording channels. During the experiments, the testers were seated in front of the LED stimulus, where all four frequencies (11.1, 12.5, 15.2, and 16.7 Hz) are presented simultaneously (Fig. 19e). The testers conducted five tasks, including a null task (eyes closed for alpha rhythms) and gazing at four different LED locations. Then, SVM and CNN models are able to achieve the EEG classifying with high accuracies using frequency–domain features. Finally, the subjects used the system and algorithm to control three target machines, including a wireless electric wheelchair, a wireless mini-vehicle, and presentation software (Fig. 19f).

#### Dynamic Time Warping

**Fig. 20:**
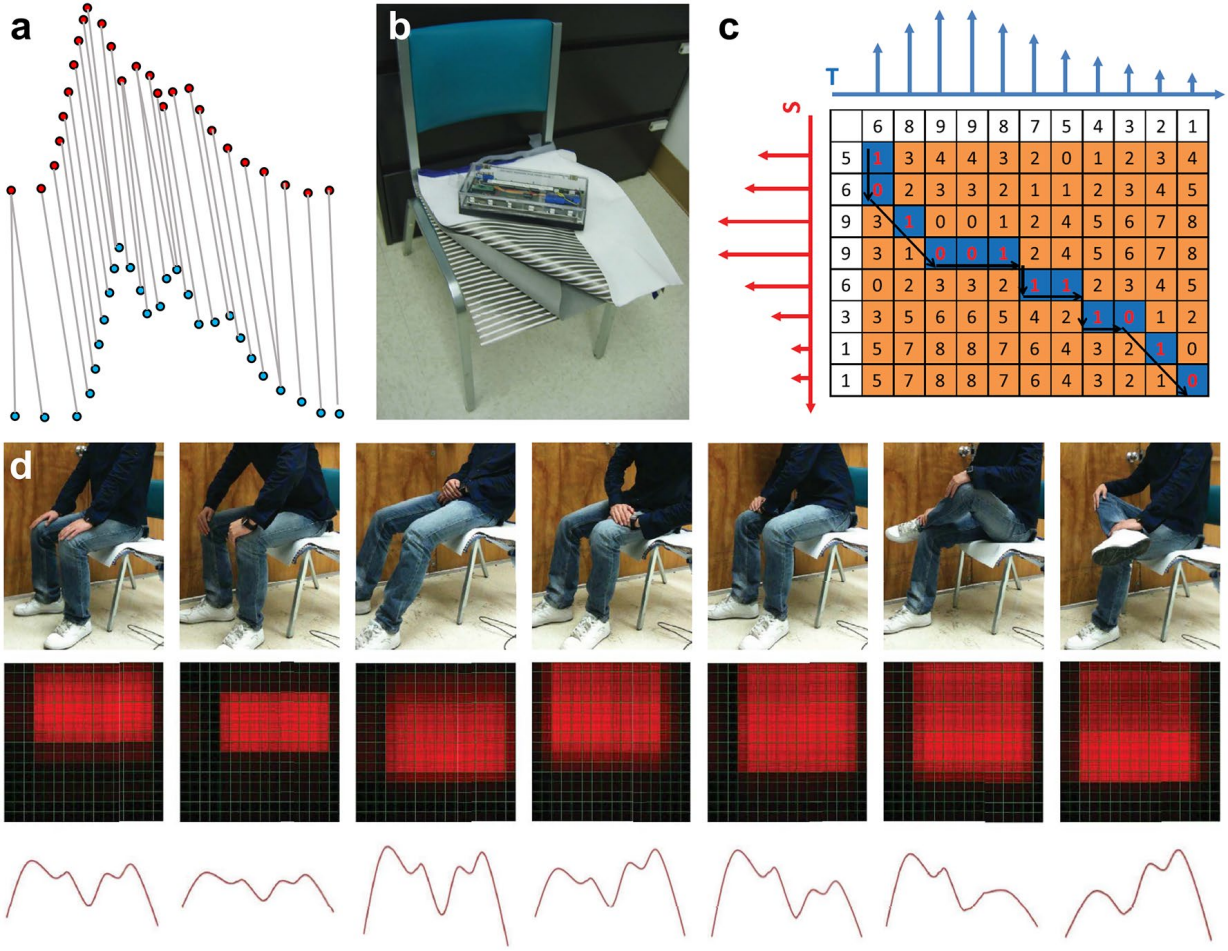
Signals detected by the soft system and processed by DTW. **a** Schematic diagram of DTW. **b** Photograph of Smart Cushion with textile sensor array. **c** A DTW example of two pressure profile sequences. **d** Sitting posture analysis: seven sitting postures (top) are evaluated and each eTextile pressure map (middle) is transformed to a corresponding pressure profile sequence (bottom). Reproduced with permission [315]. Copyright (2013), Institute of Electrical and Electronics Engineers

During machine learning process, the length of the two data that need to be analyzed may not be the same. For example, the duration of each pulse wave may be different and the speech speed of different people is different. Dynamic time warping (DTW) is a method to measure the similarity of two data with different lengths. In complex cases, the distance between two data series that cannot be effectively obtained using the traditional Euclidean distance. Comparing arrays having different lengths can be realized by constructing one-to-many and many-to-one matches in order to minimize the total distance between the two series (Fig. 20a). DTW is widely used in speech recognition, gesture recognition, data mining, and information retrieval.

Xu et al. presented a textile-based sensing system, named Smart Cushion, which can analyze the sitting posture of tester [315]. The single sensor in the array is a fiber-based yarn which is coated with piezoelectric polymer. The total sensor surface area is 10 × 10 inches, where the area of each square sensor is 5/8 × 5/8 inch. Each bus is 5/8 inch in width, and the space between sensors is 1/8 inch (Fig. 20b). Each sensor has an independent ADC to sample the pressure. Instead of processing pressure map (2D image with 256 pixels in total) directly, the data was converted into a pressure profile sequence (1D time series), which can reduce the dimension of the data and tackle the rotation issue easier (Fig. 20c). Then, the DTW was used to classify different sitting postures (Fig. 20d). The overall accuracy of the algorithm over all sitting postures can be 85.9%.

##### k-Nearest Neighbor

**Fig. 21:**
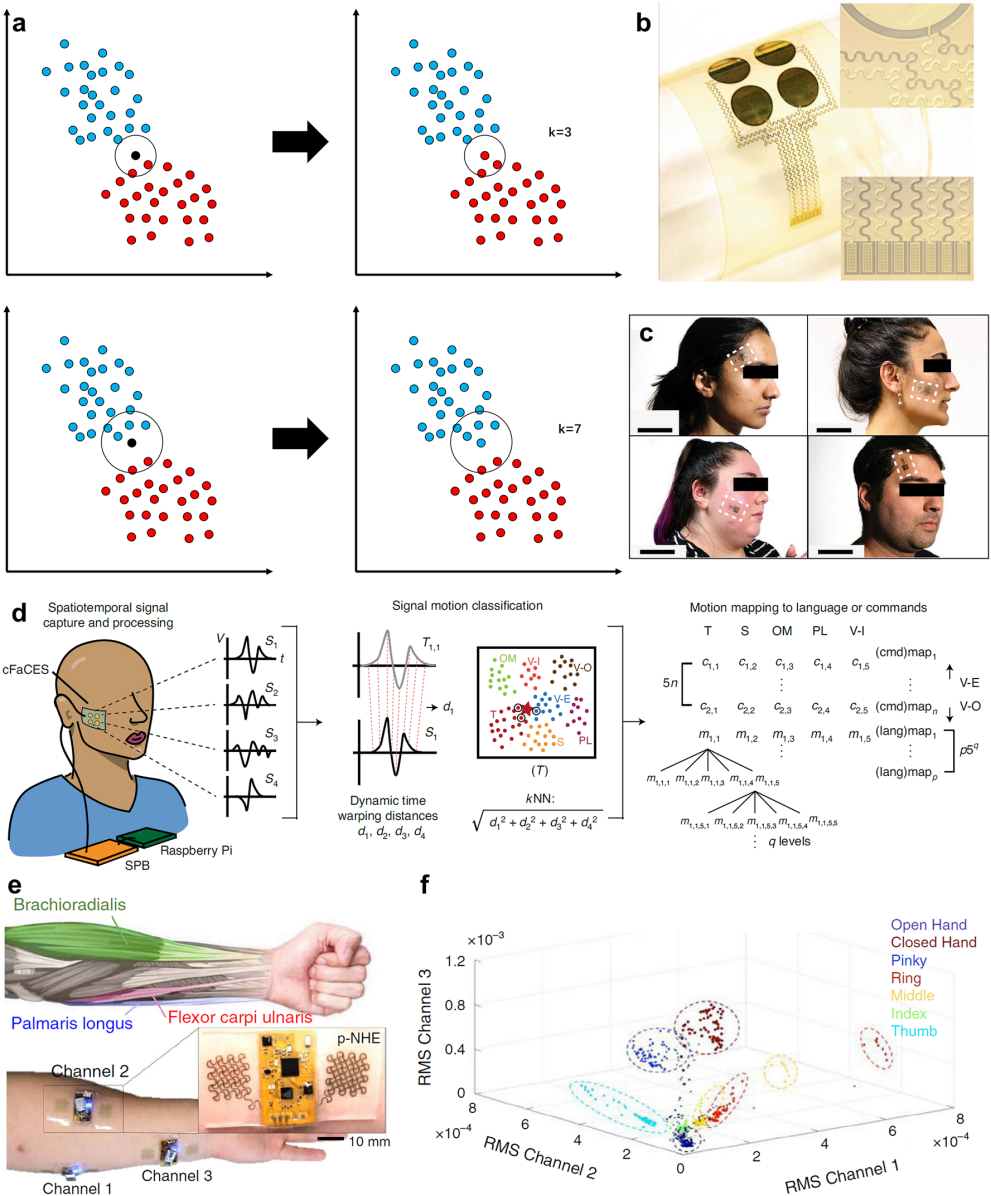
Signals detected by the soft system and processed by kNN. **a** Schematic diagram of kNN (*k* = 3 and *k* = 7). **b** Conformable sensor laminated onto a curved glass cylinder. Insets: the edge of the AlN sensing element and serpentine electrodes (top right); and the set of eight serpentine electrodes from four sensing elements connecting to Al bonding pads (bottom right). **c** cFaCES (white dashed box) laminated onto various testers at different positions of the face. **d** Schematic of the EMG RTD system. A cFaCES with four sensing elements is laminated onto the face and connected to a signal processing board (SPB) for differential signal amplification and analog-to-digital conversion. Then, the digital signal from the SPB is fed to the Raspberry Pi, which automatically detects facial motions and classifies it. The classification is based on a kNN-DTW algorithm, where the *k* = 3. The dataset contains seven motions: twitch (T), smile (S), open mouth (OM), pursed lips (PL), mouthing the vowel ‘I’ (V–I), mouthing the vowel ‘E’ (V–E), and mouthing the vowel ‘O’ (V–O), which can be mapped to five selector motions (T, S, OM, PL, and V–I) to select options within each command or language message menu. Reproduced with permission [316]. Copyright (2020), Springer Nature. **e** Schematic illustration (top) of target muscles on forearm to recognize multiple gestures and photographs (bottom) capturing three p-NHE positioned on targeted muscles, including palmaris longus, brachioradialis, and flexor carpi ulnaris. Enlarged image of the system with a circuit and electrodes. **f** 3D plot of three-channel, EMG root-mean-square (RMS) signals for clear differentiation of seven different gestures. Reproduced with permission [30]. Copyright (2020), Springer Nature

kNN is a supervised learning classification method. It has a wide range of applications and high accuracy when the sample size is large enough. When new data without labels is input, each feature of the new data is compared with the corresponding feature of the data in the training set, and then the algorithm calculates the classification label of the most similar data (nearest neighbor) of the sample. Only the first k-nearest data in the training set was selected, which is why the algorithm is called kNN. *k* is usually an integer less than 20. Finally, the most frequent label among the k-nearest data is selected as the classification of the new data. The schematic diagram of kNN (*k* = 3 and *k* = 7) is shown in Fig. 21a.

Sun et al. reported the design of an integrated system for decoding facial strains and for predicting facial kinematics [316]. Aluminum nitride (AlN) piezoelectric thin films sandwiched between two molybdenum (Mo) electrodes and encapsulated with a layer of silicon dioxide (SiO_2_) was applied as strain sensor on compliant PDMS substrates. The low-modulus substrate is comparable to the human epidermis which enables soft reversible lamination of the conformable facial code extrapolation sensor (cFaCES) (Fig. 21b). When laminated onto the facial skin, the cFaCES enables the creation of a library of motions from which a large subset of human language could be inferred (Fig. 21c). Each motion can be classified as one of the motions in the library by a real-time decoding (RTD) algorithm. The kNN–DTW model was used and runs by the onboard processor of the Raspberry Pi (Fig. 21d).

Kwon et al. developed an all-printed nanomembrane hybrid electronics system (referred as “p-NHE”), incorporating machine learning, offers multi-class and versatile HMI [30]. Ag was used as the conductive circuit traces, functionalized conductive graphene was used as the oxidation barrier for Ag as well as sensing electrodes, and PI was applied as the insulating and structural support layers. The aerosol jet-based printing method with two atomizing modes (ultrasonic and pneumatic) can realize the deposition of inks with a wide range of viscosity without the use of pattern masks or screens. The all-printed EMG devices can be used as the HMI scenarios including hand gesture-controlled wireless target controls, such as drones and a computer software. The tester wore the p-NHE to generate several motions, including open hand, closed hand, flexion of index finger, and wrist flexion (Fig. 21e). Two types of machine learning algorithms including kNN and CNN. A 3D, three-channel RMS plot from three devices shows seven distinctive clusters, generated by motions of individual fingers and hand gestures over repeated trials (Fig. 21f).

##### K-Means

**Fig. 22:**
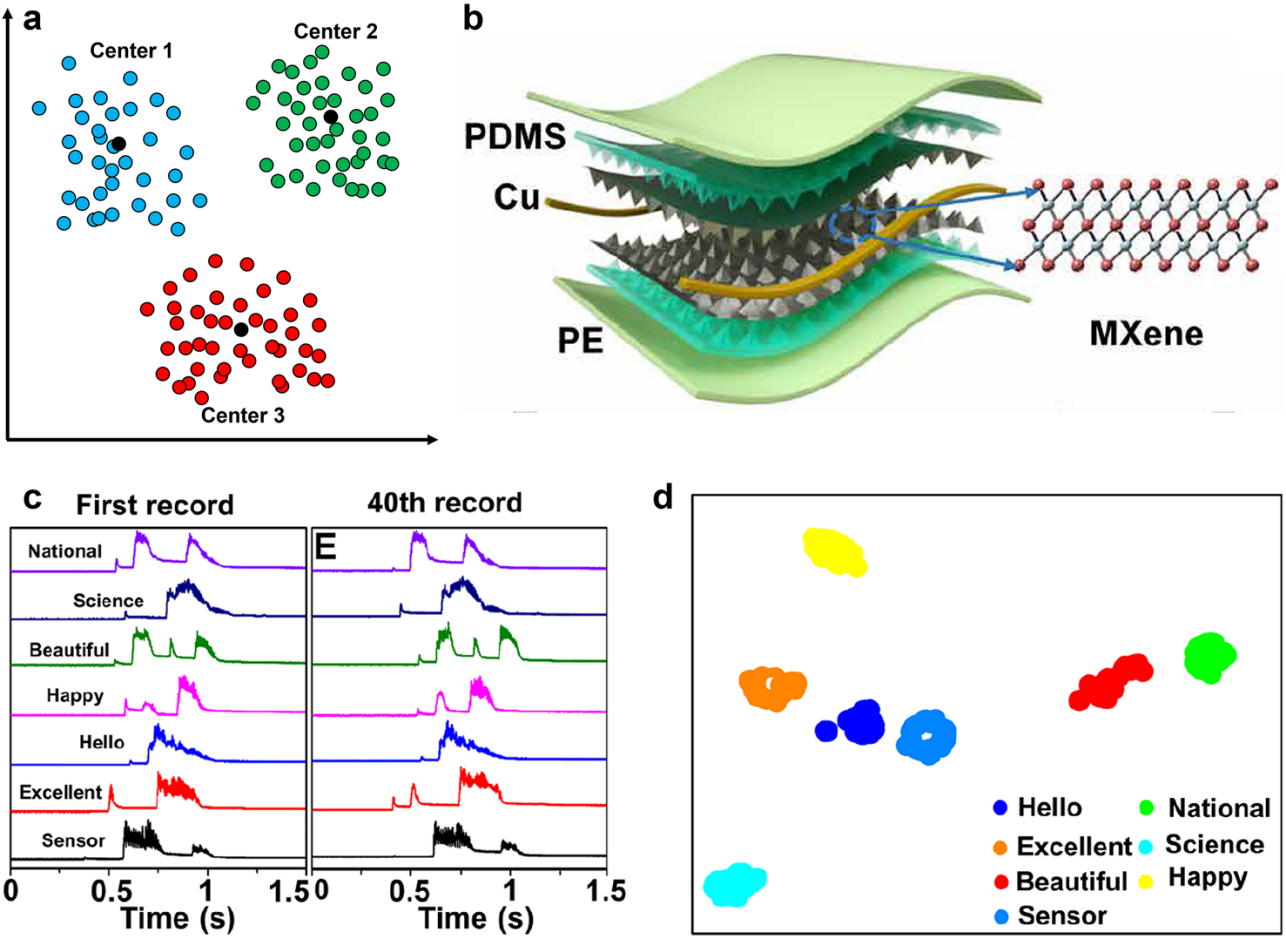
Signals detected by the soft system and processed by K-means. **a** Schematic diagram of K-means. **b** Schematic diagram of the MXene eardrum. The device consists of two layers of MXene-PDMS-PE film. **c** Normalized response waveform of seven kinds of words recorded for the 1st and 40th times by the MXene eardrum. **d** Visualizing the pronunciation information of seven kinds of words within 280 voices after dimensionality reduction. Reproduced with permission [26]. Copyright (2022), American Association for the Advancement of Science

K-means algorithm is a classical clustering method, and it is an unsupervised learning classification method. The basic idea of K-means algorithm is to cluster with *k* points as the center and classify the objects closest to them. After the iteration, the value of each cluster center is updated one by one until the best clustering result is obtained. Firstly, K objects are randomly selected from N data objects as the initial clustering centers. For the remaining objects, they are assigned to the nearest clusters according to the distance between them and the cluster centers. Then, the cluster center of each new cluster is updated to the mean value of all objects in the specific cluster. This process is repeated until the convergence of standard measure function. The K clusters have the following characteristics: the internal data of each cluster is as compact as possible, and the distance between clusters is as large as possible (Fig. 22a).

Gou et al. reported an artificial eardrum using an acoustic sensor based on 2D MXene (Ti_3_C_2_T_*x*_), which can realize the function of a human eardrum for voice detection [26]. Besides, it can also be used for the voice classification by combining with the machine learning algorithm. The artificial eardrum has two-stage amplification for the voice signals. The interlayer spacing of Ti_3_C_2_ nanoflakes (1.31 nm) can be greatly changed under an external pressure, indicating that MXene can give a sensitive mechanical response. Hence, the MXene can be regarded as the first-stage enhancement of sound sensing. In addition, the surface of PDMS substrate was fabricated into micropyramid array structure, which can provide a second-stage enhancement of sound sensing (Fig. 22b). Therefore, the MXene eardrum shows an extremely high sensitivity of 62 kPa^−1^ and a detection limit of 0.1 Pa. It can maintain a higher SNR of 50 dB from 200 to 900 Hz and remains 40 dB at higher frequencies up to 2.5 kHz. Finally, the K-means algorithm was used to classify 280 voices (seven kinds of words, namely, “sensor,” “excellent,” “hello,” “happy,” “beautiful,” “science,” and “national”) with a high accuracy of 95% (Fig. 22c, d).

##### Decision Tree

**Fig. 23:**
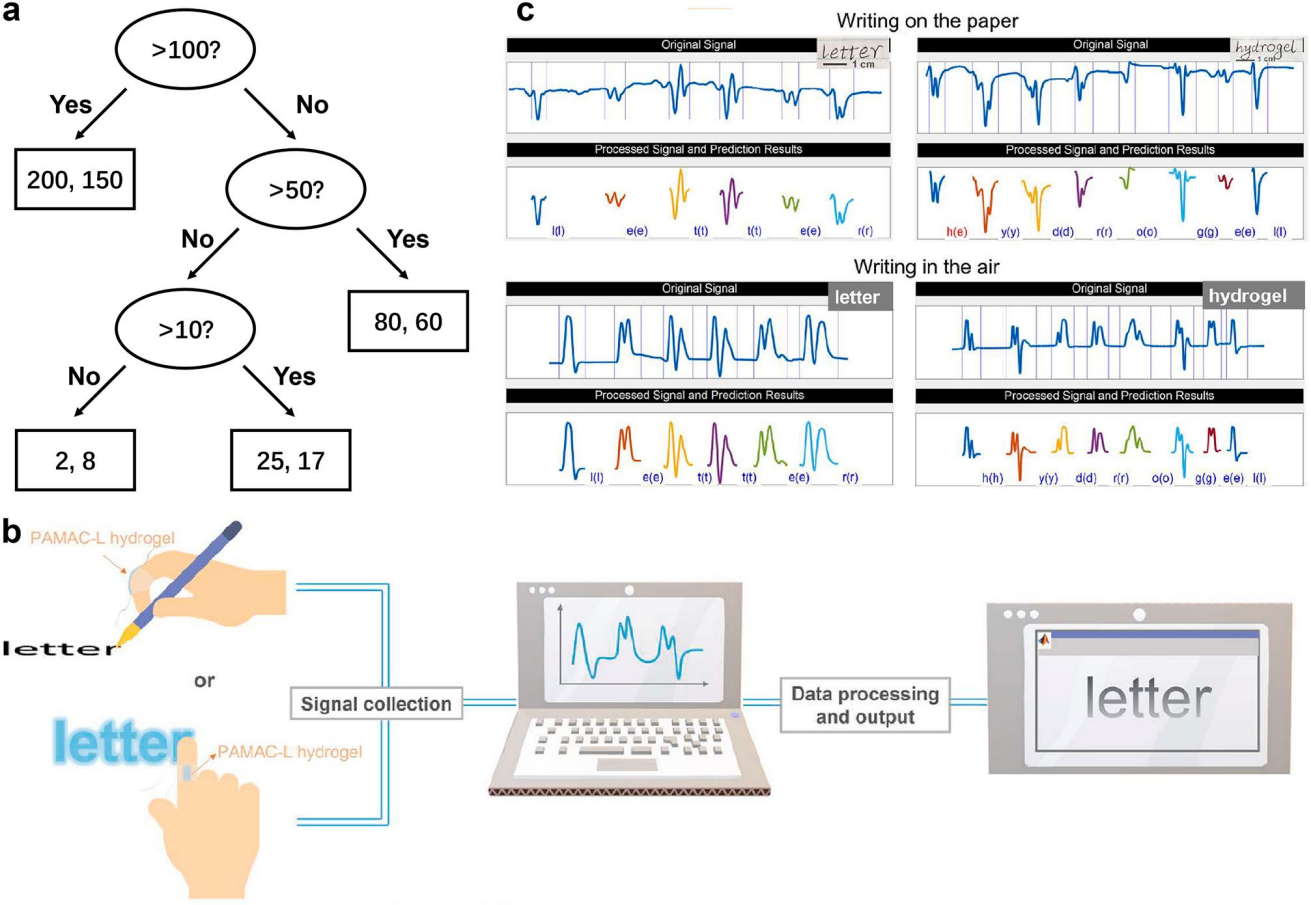
Signals detected by the soft system and processed by DT. **a** Schematic diagram of DT. **b** Schematic diagram of the handwriting recognition system. **c** Recognition results from the software for handwriting of the word “letter” and “hydrogel” on the paper and “letter”, and “hydrogel” in the air. Reproduced with permission [317]. Copyright (2022), Elsevier

A DT is a tree structure (binary tree or non-binary tree), which includes a root node, several internal nodes and several leaf nodes. The leaf node corresponds to the decision result, and each other node corresponds to an attribute test. Samples contained in each node are divided into child nodes according to the attribute test. The process of decision-making starts from the root node. Then, the classification is made according to the feature attributes, and select the output branch until reaching the leaf node, whose category is the decision result (Fig. 23a).

In the DT flowchart, the ellipse represents the judgment module, and the rectangle represents the termination module. It indicates that the conclusion has been reached and the operation can be terminated. The left and right arrows are called branches. The most important part of the DT algorithm is the construction of the DT, which is measuring the attribute selection and determine the topological structure between each feature attribute. The construction process usually has three steps: feature selection, generation of the decision tree, and pruning of the DT. The key to constructing a decision tree is the split attribute, which construct different branches at a node according to different divisions of a characteristic attribute. The goal of DT is to make the items to be classified in a split subset belong to the same category as much as possible.

Wu et al. developed an ionic conductive nanocomposite hydrogel (PAMAC-L) with ultra-stretchability and self-healing functions [317]. The PAMAC-L was fabricated by a facile one-step process. MWCNT was selected as a reinforcement agent with large aspect ratio and ultrahigh mechanical strength, which endows the hydrogel networks with excellent mechanical performances (i.e., tensile strength, stretchability and toughness up to 1.09 MPa, 4075%, and 12.8 MJ m^−3^, respectively). The reversible physical crosslinks including ionic interactions and hydrogen bonding endows the PAMAC-L hydrogel with autonomous self-healing capability. Combining machine learning algorithm, the hydrogel-based platform exhibits great recognition accuracies to human handwriting motion. Traditional handwriting on paper using a pen hold by tester was investigated. By writing 26 English letters from “*a*” to “*z*” in the size of daily handwriting, the slight movements of the finger were sensitively detected by the hydrogel sensor attached on the finger, and a series of current signals was gathered. The average recognition rates calculated from 10 writings of each word reached 87%. Another handwriting manner, writing in the air using the forefinger was monitored and recognized, by which the writing motion can also be translated into digital text without the keyboard or touch panel (Fig. 23b, c). The average recognition accuracy of 26 English letters can be 91.8%. The Machine Learning toolbox of MATLAB was used to run all available algorithms and the model showing the best predicted accuracy (Ensemble Classifier: Bagged Trees) was selected.

### Neural Network

With the development of big data, internet of thing, and computer science, neural network has been proven to be a powerful algorithm in many fields. Besides, computing science is becoming more and more powerful. Graphics processing unit (GPU), which was mainly used to display high-quality images, was found to be able to provide strong support for training neural networks on large data sets. Neural network usually consists of the three parts: input layer, hidden layer, and output layer. The input layer connects each point of the input data. Therefore, the number of neurons should be as many as the number of pixels in the input image. In the hidden layer, the data is transformed layer by layer to improve the overall similarity with the images whose labels are known. In the output layer, the final prediction results are produced. In the classification problem, the number of neurons usually equal to the kinds of labels. In the regression problem, the number of neurons usually equal to the predicted parameter numbers. Besides, there is a loss layer behind the output layer which is not usually illustrated. It compares the prediction results with the labels to provide feedback on whether the input is correctly identified or not. The comparison results are usually called loss. The number of losses is depended on the loss function such as Mean square error, Mean absolute error (for regression problem), Cross entropy, Softmax (for classification problem), etc. If the prediction is correct, the feedback from the loss layer will strengthen the activation path of the prediction result; if the prediction is wrong, the error will return along the path in reverse, and the activation conditions of the neurons in this path will be readjusted to reduce the error. This process is called back propagation, which usually based on the gradient descent method and its optimized method such as Momentum, Adagrad, and Adam.

Each layer consists of units and each unit can also be called a neural node, which is defined according to biological sources. The neural nodes also have input, output, and calculation functions. Input can be compared to dendrites of neurons, output can be compared to axons of neurons, and calculation can be compared to nuclei. At each neural node, the calculation function is shown below:

3 $$O_{j} = \sigma \left( {\mathop \sum \limits_{i} w_{ij} I_{i} + b_{j} } \right)$$

where *I*_*i*_ is the input data (the output of each neural node in the last layer), *b*_*j*_ is the bias of the neural node. *w*_*ij*_ are the connection weight from each neural node in the last layer to each neural in the current layer, which is the most important training target in the neural network algorithm. $\sigma$ is the activation function, which is the nonlinear function such as Sigmoid, Tanh, ReLU, etc. *O*_*j*_ is the output of the neural node.

There are three kinds of commonly used neural networks, fully-connected neural network (FNN), CNN, and recurrent neural network (RNN). The FNN is all built by neural nodes mentioned above, which also called the MLP. However, the calculation load of FNN is much high. For example, if we want to classify a picture with the size of 768 × 1024, we need an input layer with 786,432 neural nodes, if the hidden layer also has 786,432 neural nodes. More than 6 × 10^12^ weights are needed to calculate in this layer. If we have more layers, this is really a huge work. Besides, too many neural nodes may also cause the overfitting problems.

To solve the calculation load problem, CNN and RNN are proposed. The core of CNN is the convolution kernel, which can be the filter in the digital image processing. The convolution kernel can be used to take the local features of pictures such as the edge, texture, brightness, etc. The CNN layer can greatly decrease the computation load and increase the accuracy. Therefore, many classic CNN have been demonstrated such as LeNet [318], AlexNet [319], VGGNet [320], and ResNet [321]. The CNN is widely used in the computer vision field. To further identify individual objects and their position in the pictures, some target recognition algorithm such as You Only Look Once (YOLO) [322] and Faster R-CNN [323], where the classic CNNs are usually applied to be the backbone. With the increasing number of neural network layers, the algorithm is also called deep learning.

**Fig. 24:**
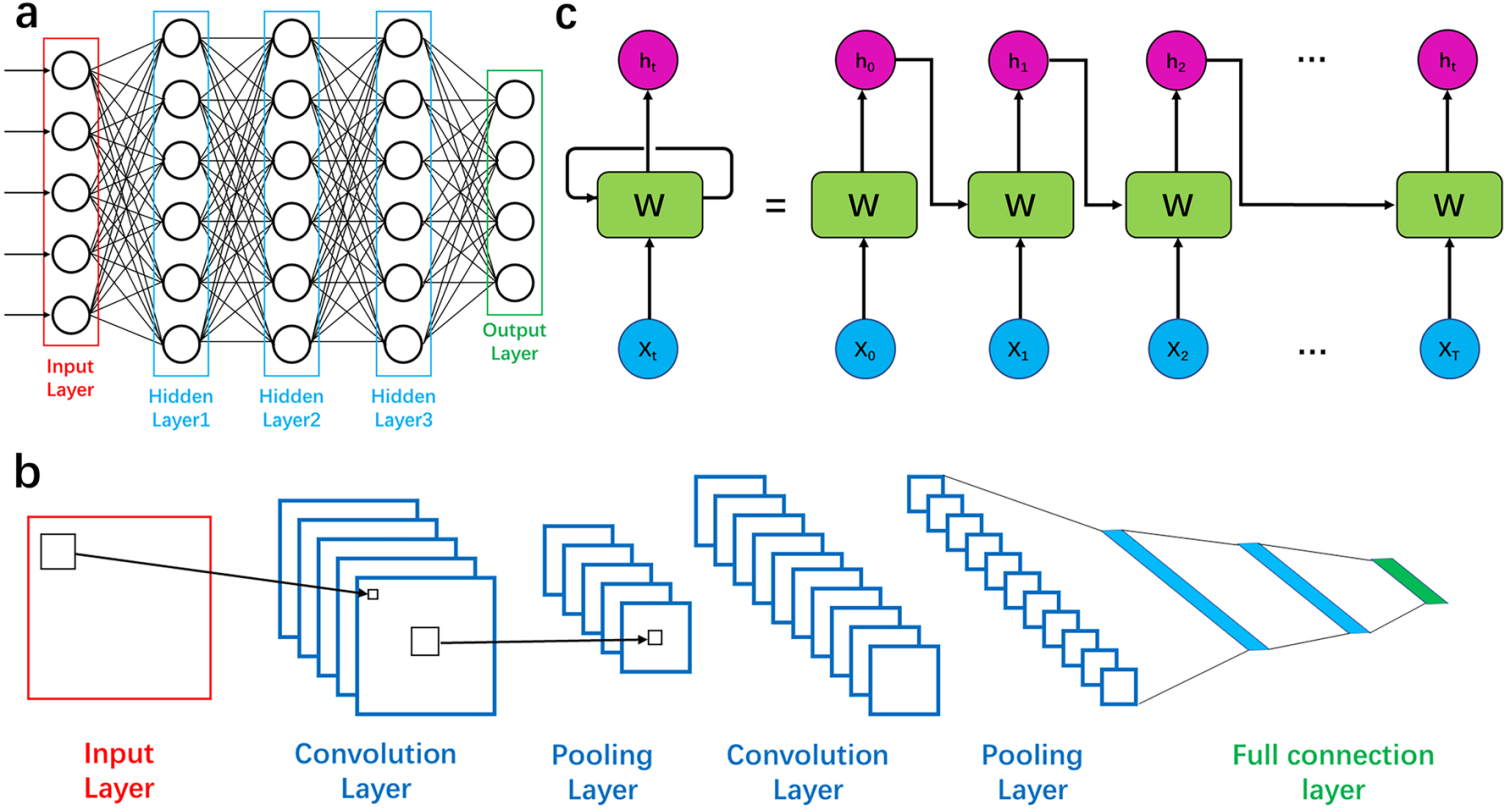
Diagram of **a** FNN, **b** CNN, and **c** RNN

Another strategy to decrease the computation load is RNN, where a unit structure can be shared repeatedly. The hidden state produced by last time can be the input to the next time. Therefore, RNN have the memory and association function, which can be used in translation.

4 $$h_{t} = \sigma \left( {w_{f} *\left[ {h_{t - 1} ,x_{t} } \right] + b_{f} } \right)$$

where *h*_*t*__ −_ _*1*_ is the hidden state of time *t* − 1, *x*_*t*_ is the input of time* t*, *w*_*f*_ is the weight of the RNN, *b*_*f*_ is the bias, and $\sigma$ is the activation function. Besides, to solve the forgotten problem, some gate-controlled RNNs have proposed, such as long short-term memory (LSTM) [324] and gated recurrent unit (GRU) [325]. The diagrams of FNN, CNN, and RNN are shown in Fig. 24.

Another popular deep learning algorithm is transformer [326], which is based on the attention mechanism to accelerate the neural network. Due to the transformer is not widely used to assist the soft device, the transformer algorithm is not discussed in this review.

In addition to building the neural network by yourself, the classic neural network can be modified to realize your functions. In other word, the neural network system can recognize and apply knowledge and skills learned in previous domains/tasks to novel domains/tasks. This process is called transfer learning.

The common physiological signals can usually be divides into two kinds, 1D statistics and 2D pictures. 1D CNN and RNN [327] is useful to the 1D statistics. 2D CNN is powerful to the pictures. The combination of CNN and RNN can improve the performance [328, 329]. Over the past decades, the ECG and imaging diagnosis (CT, MRI, and ultrasound) were widely used in the hospital, which provides huge amount statistics to the machine learning [330]. For the soft electronics, the electrophysiology sensors have been studied a lot. Besides, many neural network algorithms have been demonstrated to analyze the electrophysiology signals such as EEG [331], ECG [332], EOG [333], EMG [334], and ECoG [335]. Other signals like pulse, tactile, and respiration can also be analyzed with the neural network [336, 337]. It is a trend to combine the soft electronics with the neural network.

However, the neural network is not omnipotent, especially for the “simple” tasks. Neural network has a strong learning ability, but for some “simple” tasks, it is redundant to use this powerful tool. For example, classic machine learning methods can be used for samples with obvious features and few classification types. Neural network can be used when the features are not obvious (such as ECG and EEG) with many classification types (gesture recognition).

#### Fully-Connected Neural Network

**Fig. 25:**
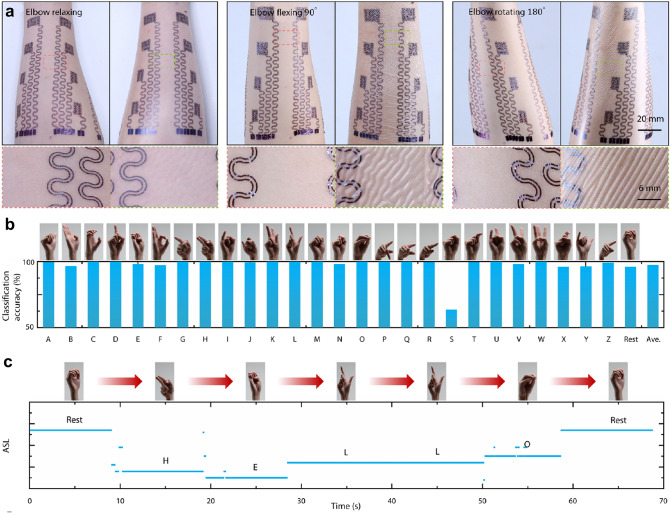
FNN for recognizing ASL. **a** Photographs and corresponding optical micrographs of large-area epidermal electrodes with and without substrate attached on forearm. **b** Classification accuracy of 26 ASL alphabets and a rest gesture, respectively. **c** One trial of the continuous recognition of the sign language saying “HELLO” consists of single letter. Reproduced with permission [338]. Copyright (2020), American Association for the Advancement of Science

Wang et al. reported a large-area, soft, breathable, substrate- and encapsulation-free electrodes, which can be designed into transformable filamentary serpentines and rapidly fabricated by cut-and-paste method [338]. The epidermal electrodes can capture various biopotentials (16-channel sEMG) in high fidelity at scale (Fig. 25a). The Cartan transfer printing method can realize an open-mesh filamentary serpentine network to be transferred on human skin (whole chest, forearm, and neck.) conformally without any substrate, even on the deformed skin. 16-channel sEMG on the forearm was recorded using the electrodes, which can be used to recognize the American Sign Language (ASL) (Fig. 25b). All electrophysiological data were filtered with a high-pass filter. Then, two typical time–domain features, including mean absolute value and root mean square and mean frequency, were extracted and imported to an FNN-based pattern recognition in MATLAB. After trained with half of the data, the network was validated with the remaining and then used to carry out the continuous recognitions or the manipulation of a robotic hand. The classification accuracy of 26 alphabets and the rest gesture after verification can be very high accuracy (over 96%) and some even 100%. The average classification accuracy is as high as 97.4%. Real-time ASL recognition through sEMG was demonstrated by a participant continuously expressing “HELLO” (Fig. 25c).

#### Convolutional Neural Network

**Fig. 26:**
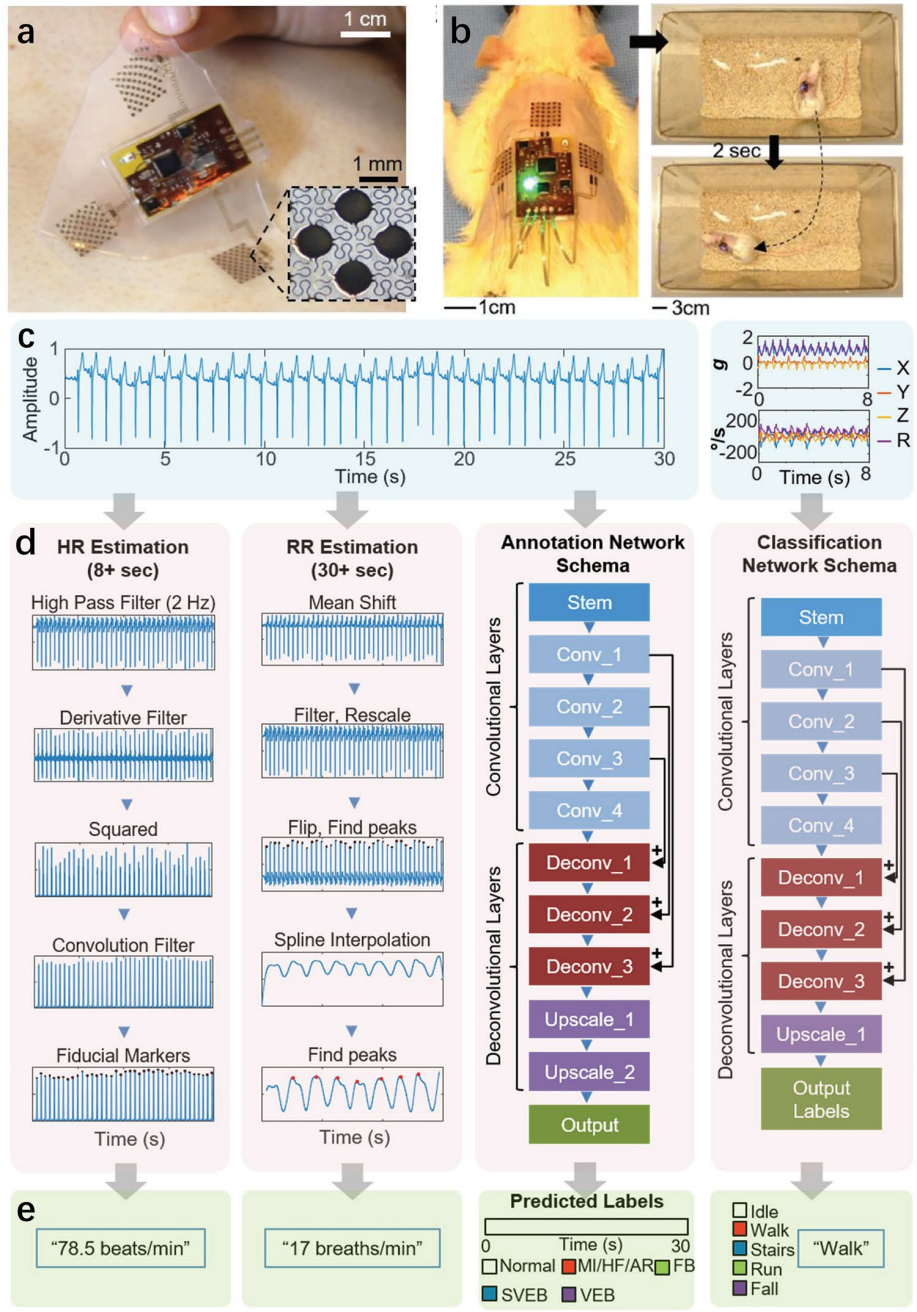
ECG signals detected by soft system and analyzed by CNN. **a** Photograph of an SHE system laminated on the skin without the use of adhesives. Inset is the patterned Au electrodes. **b** In vivo animal study of the SHE system, involving ambulatory ECG monitoring of a rat model on its shaved back. **c** A set of ECG signals measured by SHE system (left) and motion activity, measured by change of acceleration (top right) and orientation (bottom right). **d** Overview of data processing methods consisting of four columns: HR estimation process, RR estimation process, ECG classification CNN, and Motion classification CNN. **e** Each column showing the outputs from the column in **d**, in the order of HR, RR, ECG annotation, and motion activity class. Reproduced with permission [339]. Copyright (2019), Wiley–VCH

In 2019, Kim et al. developed an all-in-one, wireless, stretchable hybrid electronics (SHE) system to detected motion, respiration, and the ECG signals [339]. The Au/Cr electrodes and Cu connector between electrodes and flexible printed circuit boards (FPCB) are fabricated by the soft process. With the extremely low-modulus Ecoflex 1:2 as the substrate, the system can realize an intimate contact intimate skin contact (Fig. 26a). With the Tegaderm as the substrate, the SHE system can realize the in vivo cardiac monitoring of rat (Fig. 26b). To analyze the ECG, acceleration, and orientation data automatically, CNN with sequence-to-sequence annotation concept has been used to predict the motion state such as idle, walk, run, and fall. Besides, four cardiac diseases can also be classified by the CNN such as (1) myocardial infarction (MI), heart failure (HF), and miscellaneous arrhythmia (AR), (2) fusion beat (FB), (3) supraventricular ectopic beats (SVEB), and (4) ventricular ectopic beats (VEB). Heart rate (HR) and respiratory rate (RR) can also be extract from the ECG signals (Fig. 26c–e).

**Fig. 27:**
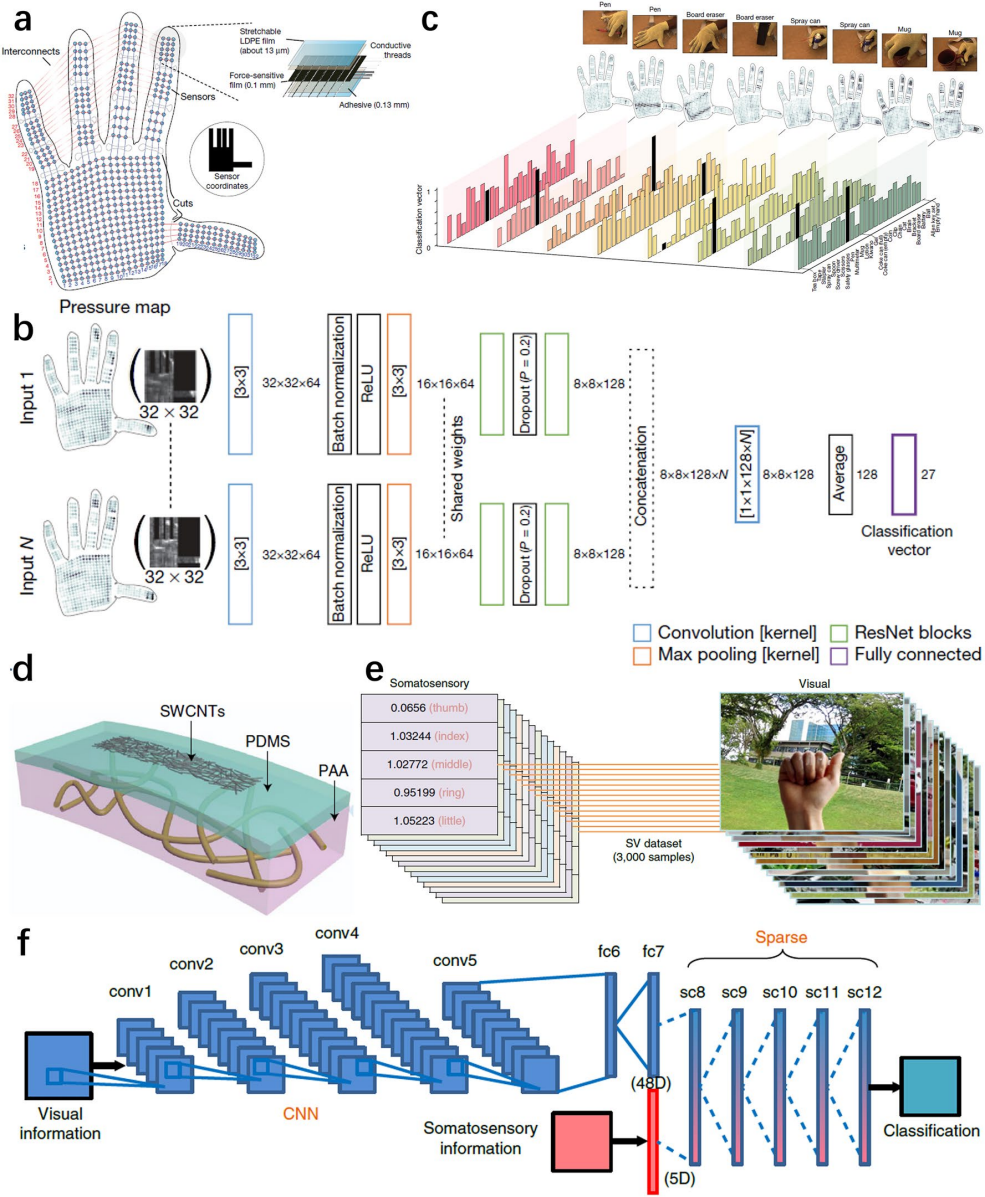
Mechanical signals detected by soft sensor and analyzed by CNN. **a** STAG architecture consisting of the individual locations of the 548 sensors, along with the interconnects, slot, and 64 electrodes. The piezoresistive sensor array can be divided to different architectures. **b** CNN architecture applied for identifying objects with the input N arrays of tactile data (32 × 32 arrays). **c** Tactile maps, corresponding visual images, and the classification results from single tactile map inputs. The ground-truth object labels are marked in black. Reproduced with permission [340]. Copyright (2019), Springer Nature. **d** Diagram of the conformable, transparent, and adhesive stretchable strain sensor. **e** Illustration of the SV dataset containing 3000 SV samples. **f** Diagram of the BSV algorithm. Reproduced with permission [342]. Copyright (2020), Springer Nature

Sundaram et al. fabricated a scalable tactile glove (STAG) covering the full hand with 548 press sensors based on commercial force-sensitive film (FSF) as shown in Fig. 27a [340]. The FSF is patterned by the laser cutting, and the total cost of STAG is only about US$10. When touching different objects, the stress distribution map recorded by the STAG are different. Then, they used the transfer learning method mentioned above to identify 26 objects. The 32 × 32 map is put into the adjusted ResNet-18 CNN (Fig. 27b). However, the held methods will influence the classify result (Fig. 27c). In addition to identifying objects, the weights of held objects can also be estimated. Li et al. fabricated a multisensory tactile system to recognize objects, which combined the soft temperature (Pt/Cr thermosensitive ribbons) and pressure (porous PDMS/AgNPs) sensors [341]. They built a 3-layer FNN to identify cotton, sponge, tangerine, human hand, mango, and napkin.

Inspired by the somatosensory-visual (SV) fusion hierarchy in the brain, Wang et al. built a bioinspired somatosensory-visual (BSV) algorithm consists of three neural networks [342]. The somatosensory signals were detected by transparent and skin-like stretchable strain sensors consisted of SWCNT as the sensing component, PDMS layer and adhesive poly (acrylic acid) hydrogel layer (Fig. 27d). Then, they built a dataset consists of 3000 SV samples with 10 categories of hand gestures. Each sample containing an image of a hand gesture with a complex background and one group of strain data detected by the five strain sensors over the knuckle of the thumb, index, middle, ring, and little finger, respectively (Fig. 27e). The BSV algorithm with five-dimensional somatosensory vector, an AlexNet CNN and a five-layer sparse neural network can achieve a recognition accuracy of 100% (Fig. 27f). Finally, they built an auto-recognition and feedback system based on BSV algorithm to guide a robot by hand gesture even in non-ideal conditions such as dark.

**Fig. 28:**
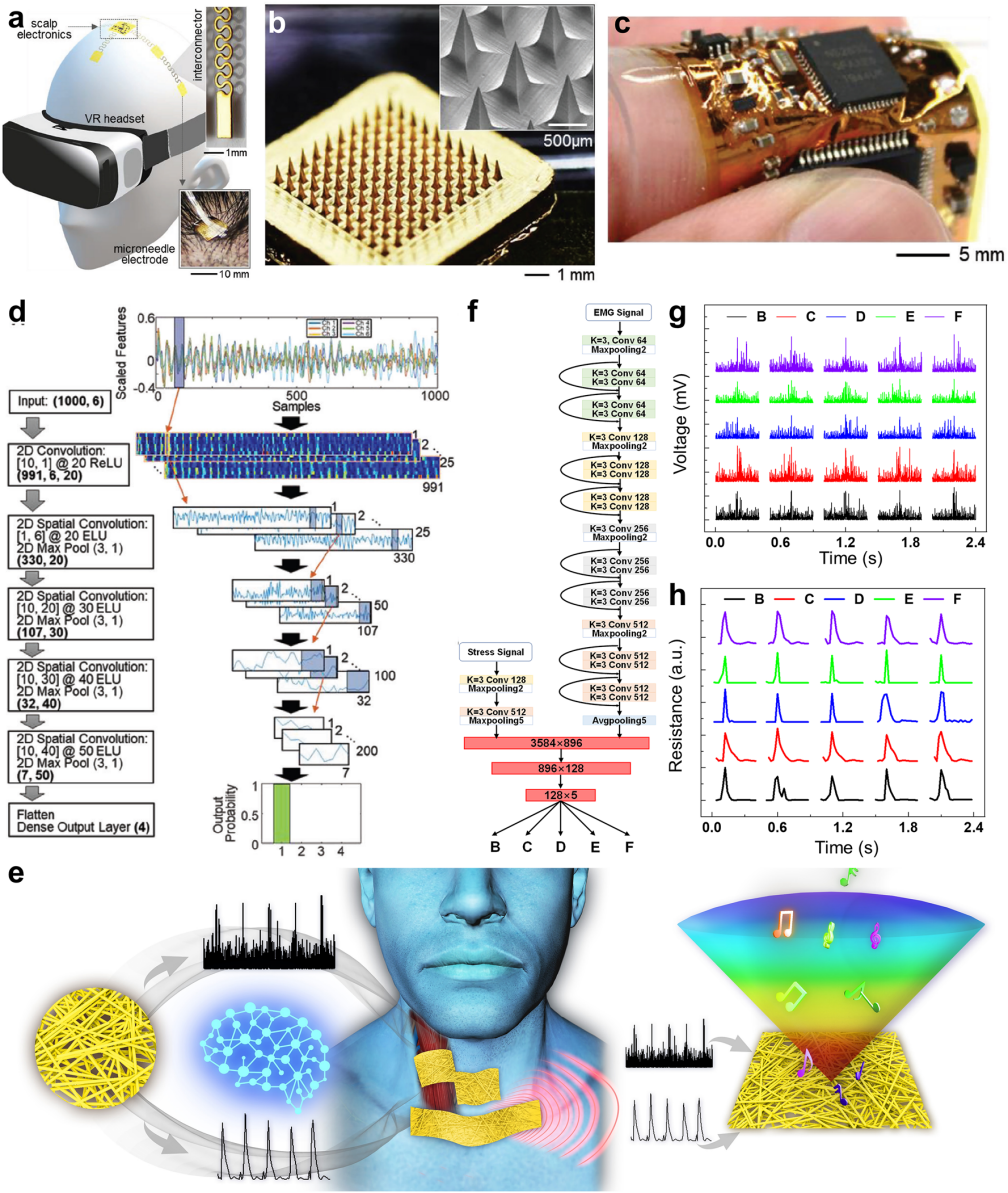
CNN for classification task. **a** Illustration of a tester wearing VR headset and scalp electronics with stretchable interconnectors and a soft microneedle electrode. **b** Zoomed-in photograph of a microneedle array along with a magnified SEM image (inset). **c** Photograph of a soft wireless circuit with integrated chips, which has mechanical compliance. **d** Detailed illustration of a spatial CNN model with hidden layers of brain signals acquired from six EEG channels, which demonstrates the capability of decomposing spatial features. Reproduced with permission [343]. Copyright (2021), Wiley–VCH. **e** Diagram of the intelligent nanomesh artificial throat, including nanomesh voice detecting part (strain sensor and EMG electrodes), electromyogram-strain synergetic CNN algorithm, and nanomesh sound source. **f** Diagram of the CNN algorithm consisting of modified 1D ResNet18 (EMG part) and common two-layer CNN (strain part). **g** Typical EMG signals detected by the Au nanomesh electrodes when tester spoke “B”, “C”, “D”, “E”, and “F”. **h** Typical normalized strain signals detected by the Au/PU nanomesh strain sensor when tester spoke “B”, “C”, “D”, “E”, and “F”. Reproduced with permission [182]. Copyright (2022), Elsevier

Mahmood et al. developed a wireless scalp electronic system with virtual reality (VR) for real-time, continuous classification of motor imagery brain signals (Fig. 28a) [343]. The system consists of three major components: multiple and high-density soft microneedle electrodes (The area of each electrode set is about 36 mm^2^) (Fig. 28b) for mounting on the hairy scalp, laser-cut stretchable and soft interconnects, and a low-profile, soft circuit (Fig. 28c). In addition, the inclusion of a VR component was used as a convenient and immersive training environment to assist with motor visualization. The approximate positions of the six electrodes corresponding with the standard 10–10 electrodes placement system are Fz, C5, C3, C4, C6, and POz, with the reference electrode at Cz, and the ground electrode placed at the mastoid. Assisted by a VR interface, motor imagery (MI) tasks were realized by combining the system with the CNN-based machine learning algorithm (Fig. 28d). Testers were asked to imagine the actions of opening and closing their hands, as well as depressing a pedal with both feet in the first person for the tasks. In the VR examination, the testers were provided with clear visual guidance on what they should be imagining by VR, using animated disembodied limbs within the normal field of view. Finally, the system with only 6 EEG channels can realize a high accuracy of 93.22 ± 1.33% for four classes.

Our group developed an intelligent artificial throat by nanomesh containing strain sensor part (Au/PU nanomesh), EMG sensor part (Au/PU nanomesh), sound source (Au/PVA nanomesh) (Fig. 28e) [182]. The nanomesh was fabricated by electrospinning and sputtering. The Au/PU nanomesh can be used as the strain sensor with high sensitivity, large work range, and good stability. The Au nanomesh can be applied as the physiological electrodes whose impendence is even lower than the commercial gel electrodes to detect the ECG and EMG signal. The Au/PVA nanomesh with good low heat capacity, high thermal conductivity, and electronic conductivity can be used as sound source. In addition, the nanomesh has good water permeability, stability, and conformal property with skin. Taken the advantage of nanomesh, the EMG-strain synergetic artificial throat was realized. Combined with the intelligent synergetic convolution neural network (SCNN) algorithm (Fig. 28f), the artificial throat can distinguish the transient voice. After attaching nanomesh on the neck of tester without any tape, the EMG part using Au nanomesh was used to monitor the EMG signals when tester was speaking letters of alphabet “B”, “C”, “D”, “E”, and “F”. The strain part using Au/PU nanomesh was used to monitor the vibration of throat when tester was speaking letters of alphabet “B”, “C”, “D”, “E”, and “F” (Fig. 28g, h). Finally, a SCNN algorithm built by 1D ResNet18 (EMG part) and two-layer CNN (strain part) is demonstrated to distinguish the transitory voice signals detected by the nanomesh strain sensor and electrodes with the high accuracy of 98.9%.

#### Recurrent Neural Network

**Fig. 29:**
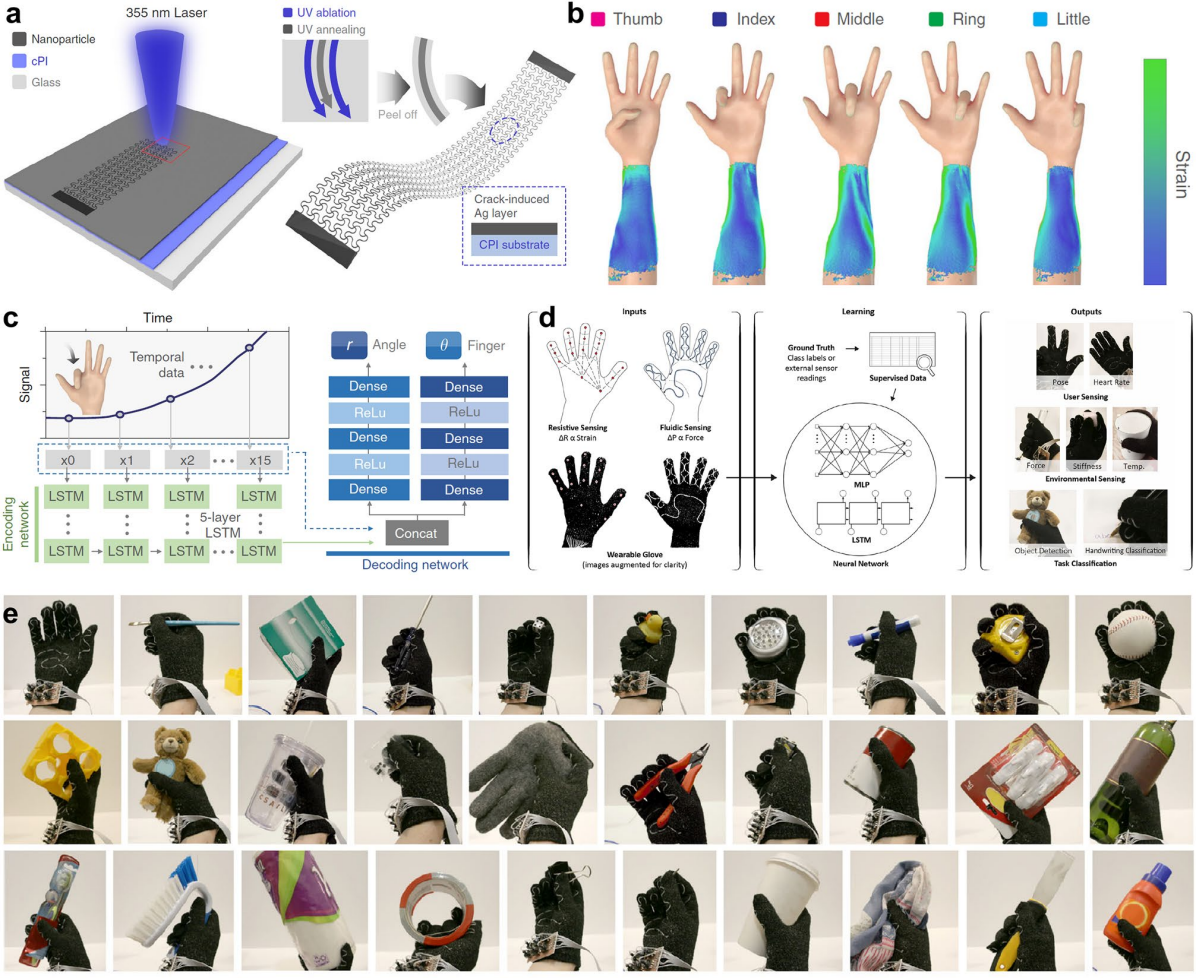
RNN for the classification task. **a** Schematic depicting the patterning and crack fabrication by laser fabrication. **b** Depiction of skin deformations for different finger bending motions. **c** Neural network composed of an encoding network and a decoding network. LSTM layers are used in encoding network to analyze temporal sensor patterns to generate latent vectors. Two independent dense layers map created latent vectors to the metric space expressing hand motions. Dropout is used as the regularization technique to prevent the network to be overfitted to a single use case. Reproduced with permission [344]. Copyright (2020), Springer Nature. **d** Resistive sensing and fluidic sensing are combined to provide signals for strain and contact force information, respectively. These signals are fed into a pretrained neural network architecture for inference, which is trained from captured data labeled with ground-truth knowledge. Depending on the neural network, the output of the network provides inference for a wide variety of downstream tasks. Electrode points which are usually not visible have been colored and enlarged in the left-hand images for clarity. **e** Object classification dataset, with associated grasps. Reproduced with permission [34]. Copyright (2020), Wiley–VCH

Kim et al. developed a novel e-skin sensor system integrated with a RNN that captures dynamic finger motions without creating a sensor network [344]. The sensor can detect subtle deformations from the unique laser-induced crack structures. Colorless polyimide (CPI) is first uniformly coated on a glass substrate and AgNPs ink is then spin-coated over the CPI layer. The bilayer of AgNPs and PI is patterned into the serpentine structure by laser ablation, and then the AgNPs was selectively converted into a crack-induced layer (Fig. 29a). After transferring the patterned structure to the PDMS, the sensor can be directly mounted on the skin. A topographical movement of the wrist can be triggered by the epicentral finger motion, with the attached crack-based sensor producing a signal, which contains the information of finger motion (Fig. 29b). Then, LSTM was designed to accomplish two tasks: analyzing sensor signal patterns into a latent space encapsulating temporal sensor behavior and mapping latent vectors to the finger motion metric space corresponding to encoding and decoding network. To perform the classification task, the decoding network was modified to a three-layered dense block producing 8-dimensional vector output, which corresponds to eight classes (Fig. 29c). The classifying accuracy of finger motions and noises is 96.2% in average and 92.9% in the worst case for little finger motions.

Hughes et al. developed a fully soft, wearable glove, which is capable of real-time hand pose reconstruction, environment sensing, and task classification (Fig. 29d) [34]. The wearable glove incorporates two novel sensing technologies: a resistive sensing architecture (strain sensor) and a fluidic sensing architecture (pressure sensor). A fully conductive thread glove as a substrate, and two kinds of sensors was sewed onto it. Electrode connections have been made at points between joints to optimize the capture of strain caused by joint bending. The 16 connection points (corresponding to 16 strain sensors) chosen provide one connection point per major facet of the hand, consisting of one connection per finger joint and two on the palm. The pressure sensors use a soft tube with 2 mm outer diameter. One end is sealed by knotting, and the other end is given an airtight connection to the pressure transducer. Six pressure sensors were used: one on each of the five fingers, and one on the palm. By using MLP and LSTM, the regression and classification function can be both realized. Although not a mechanical task, both pressure sensors and resistive sensors can be used to estimate the temperature of grasped object. Changes in temperature have a direct correlation with the resistivity of the knit glove. In addition, the pressure in the fluid is correlated temperature through the ideal gas law. The LSTM network realize the regression task, for example estimating an average temperature with the estimation error of just over 1 ℃. The MLP and LSTM can identify the classification of the natural grasps of 30 objects with almost 100% classification success (Fig. 29e).

#### Spiking Neural Network

**Fig. 30:**
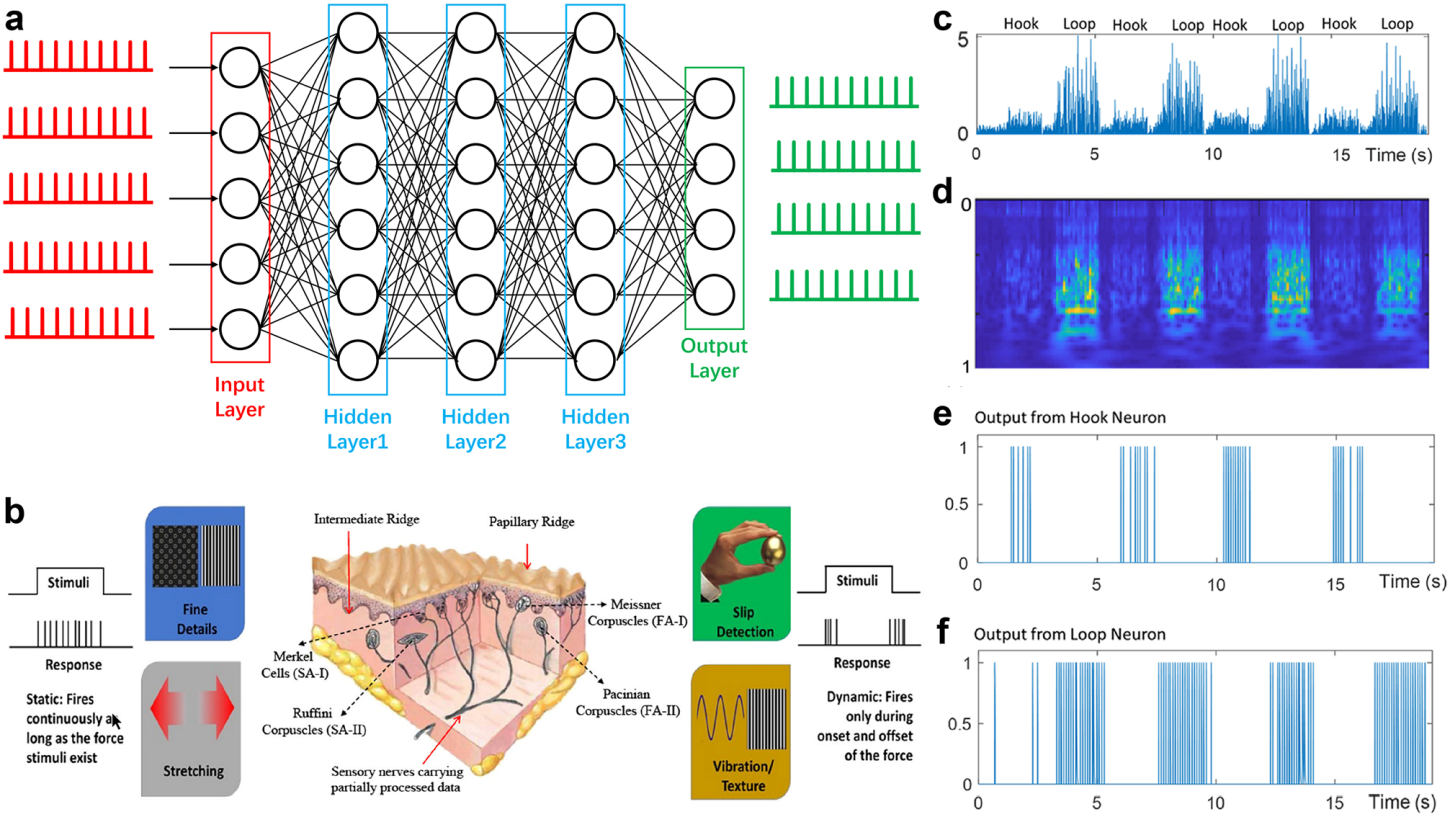
Signals detected by the soft system and processed by SNN. **a** Schematic diagram of SNN. **b** Mechanoreceptors in the human skin which enable the tactile sensation. SA mechanoreceptors respond with continuous spikes during the static stimuli and the FA mechanoreceptors respond with spikes during the transition or the dynamic part of the stimuli. **c** A typical recorded signal from the dynamic scan when the sensor touching different texture (Hook and Loop). **d** Gabor wavelet scalogram of different texture signals (Hook and Loop) corresponding to **c**. Output from the tempotron SNN classifier neuron corresponding to **e** Hook and **f** Loop. Reproduced with permission [345]. Copyright (2019), Wiley–VCH

As mentioned above, the current artificial neural network (ANN) usually receives continuous values and outputting continuous values. Although ANN has enabled us to achieve breakthroughs in many fields, they are not precise in biology and cannot imitate the operation mechanism of biological brain neurons. Spiking neural network (SNN) also calculate the appropriate synaptic weight matrix for the given multiple input pulse sequences and multiple-target pulse sequences, so that the output pulse sequences and the corresponding target pulse sequences are as close as possible. In other words, SNN also tries to minimal the error evaluation functions. Since the transmission of information is based on pulses, the input of the network needs additional coding, such as frequency coding and time coding, to convert the data into the pulses form (Fig. 30a). Therefore, the energy consumption of SNN is lower, and each neuron works independently. Some neurons will not work when they do not receive input strong enough.

Navaraj et al. developed a tactile sensor based on piezoelectric oxide semiconductor field effect transistors (POSFETs) for tactile sensing [345], which exhibits a sensitivity of 2.28 kPa^−1^. The tactile sensor can mimic the static and dynamic force feedback from the slow-adapting (SA) receptors and fast-adapting (FA) receptors of the skin (Fig. 30b). The sensor stack was integrated on the distal phalange of the index finger of a robotic/prosthetic hand, which was mounted as an end effector with a custom 3D-printed fixture to a 6-DOF UR5 robot. Biological research suggests the tactile information is processed with the temporal frequency channels. Inspired by this, windowed Gabor wavelet transform (GWT) was used to processed the data, which offers localization in time and frequency, thereby capturing temporal variation and biologically plausible. Various textured surfaces (Hook and Loop) were classified by SNN tempotron classifier system. The information about fingertip touching different texture was assumed to be transmitted within a single spike when this fast response was considered together with the various associated delays such as peripheral nerve conduction, the generation of muscular force, and processing. By temporal coding, the signal was encoded and transmitted into latency-coded spike trains. Stronger the amplitude, faster the spike was elicited within the time span (Fig. 30c–f). The weights of the input synapses were trained to emit an output spike to match the target category using a supervised learning strategy. The signal processed by STFT-based approach provides a maximum classification accuracy of 95.3%, whereas the GWT-based approach can be up to 99.45% for the same windowed time.

### Neural Network Run on Soft Electronics

The algorithms discussed above are run in CPU or GPU of computers or servers. Therefore, the data interaction is inevitable during the practical application, which complicates the circuit of soft system and increases power consumption. It’s meaningful to run the algorithms in site by the soft devices. For the neural network algorithm, the training process can be in situ or online. The weight and bias can be saved in the soft electronics.

**Fig. 31:**
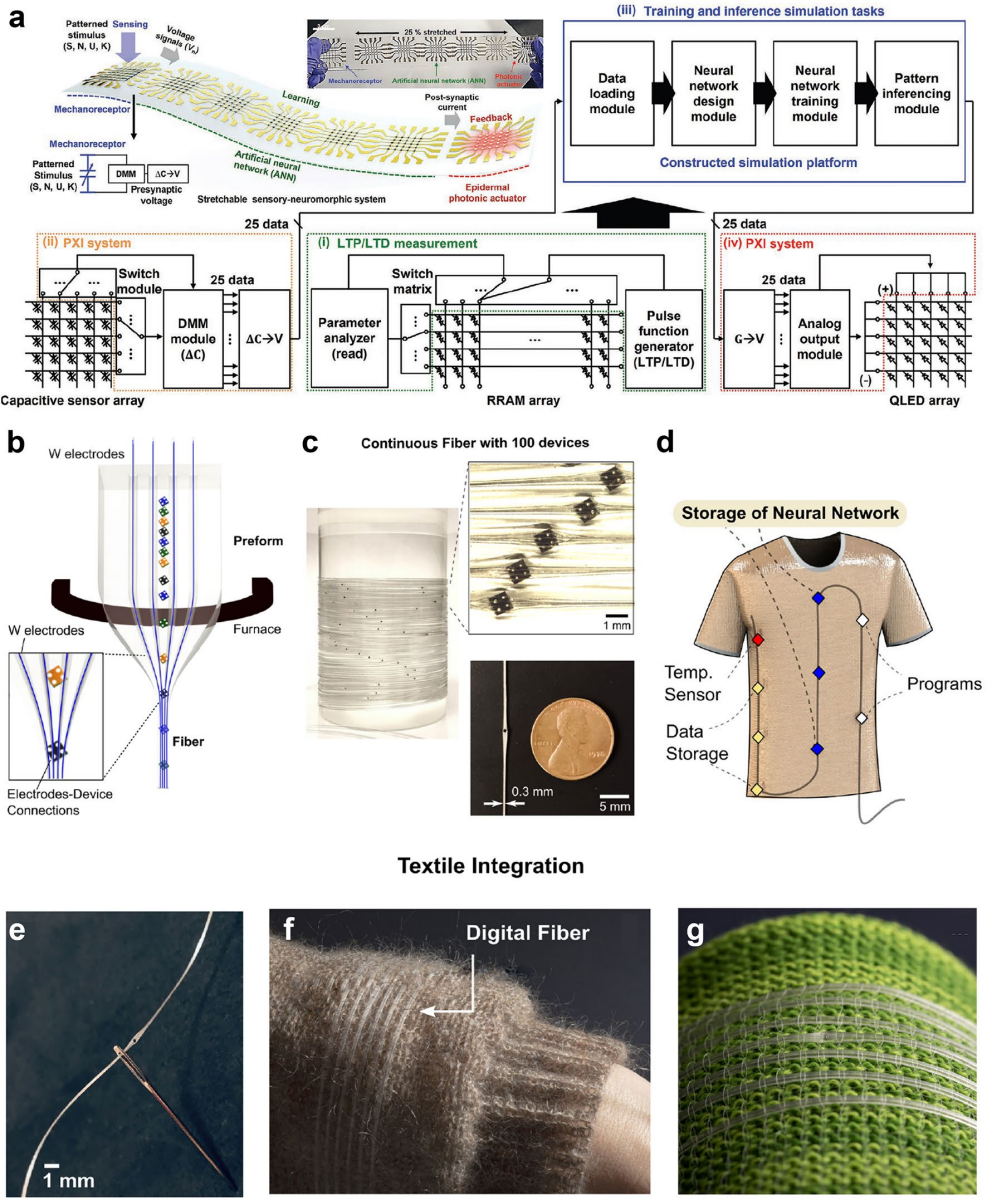
Neural network worked on soft device. **a** Schematic of the SSNS (top left, inset image denotes photographs of the stretched SSNS) and overall operation process of the SSNS. (i) Extraction of LTP/LTD data from the stretchable artificial synapse. (ii) Capacitive measurement by the pressure sensor array. (iii) Training/inferencing process in online MLP using the extracted LTP/LTD data. (iv) Inferencing process in online MLP to deliver feedback processed information to the QLED photonic actuator. Reproduced with permission [346]. Copyright (2021), Wiley–VCH. **b** Thermal drawing of the digital fiber performed by feeding conductive W wires into the empty channels. At the preform level, the W wires are spaced far apart with a polymeric barrier separating the device and the wires. The inset showing the converging of the four W electrodes toward the four pads at the necking region of the preform-to-fiber transition. **c** Photograph of a spool containing continuous digital fibers with 100 embedded devices (left). Magnified optical image of the fiber array showing that the digital devices are all rotated to the critical angle with connections to wire electrodes (right). Photograph showing the size difference between the fiber and a coin (bottom). **d** Schematic of the shirt integrated with a digital fiber including sensors, data storage, customizable programmers, and a neural network stored within its memory devices. Integration of the digital fiber **e** through a needle, **f** in the sleeve of a sweater, and **g** in a cotton-based fabric. Reproduced with permission [347]. Copyright (2021), Springer Nature

Kim et al. realized a bioinspired stretchable sensory-neuromorphic system (SSNS), comprising an artificial mechanoreceptor, artificial synapse, and epidermal photonic actuator (constructed using a capacitive pressure sensor array, RRAM array, and quantum dot light emitting diode (QLED) array, respectively) (Fig. 31a) [346]. The system has three vital functions: (i) the artificial mechanoreceptor for converting physical input into electrical potential, (ii) the artificial synapse that uses a neural network based on training/inferencing, and (iii) the epidermal photonic actuator for color change. An stretchable printable conductor that consists of PDMS, 4,4-methylenebis(phenyl urea), and isophorone bisurea (PDMS-MPU_0.4_-IU_0.6_) and Ag flakes was developed. Based on the stretchable printed conductor, an artificial mechanoreceptor (5 × 5 capacitive touch sensing array) was fabricated, which can significantly improve the cell density and scalability of the biomimetic sensor. To impart synaptic functionality into SSNS, an RRAM module was used with an Al/TiO_2_/Al layered structure (cross-sectional area of active region: 250 × 250 μm^2^) as a neuromorphic device featuring cognitive computation. The RRAM cells were fabricated on the SiO_2_/Si wafer, and transfer printed onto the stretchable substrate. Finally, each island (500 × 500 μm^2^) was then bridged via stretchable printed interconnects. The QLED featured a PDMS/PI/Ag/ZnO NPs/quantum dots/4,4-bis(9-carbazolyl)-biphenyl)/Molybdenum oxide/Ag/SiO_2_ structure. Au auxiliary electrodes were used to reduce the contact resistance between the QLED electrodes and the sinter-free stretchable interconnects. To train the neural networks, the synapse weights were updated using a backpropagation algorithm. The applied voltage signals were multiplied by synapse weights (*W*_*n,m*_) and summed at the output neurons, where the synapse weight was defined as synapse conductance (*W* = *G*). The output neuron layer was transformed using a sigmoid activation function to obtain the output neuron signals. After trained for 140 epochs, a 100% pattern recognition accuracy was realized. Finally, the feedback actuation was visualized via the 5 × 5 QLED arrays in response to inference results (“S,” “N,” “U,” and “K” patterns).

Loke et al. developed a scalable preform-to-fiber approach to producing tens of meters of soft fiber containing hundreds of interspersed, digital temperature sensors, and memory devices with a memory density of ~ 7.6 × 10^5^ bits per meter [347]. The fiber has the ability to measure and store physiological parameters and harbors the neural networks required to infer sensory data. During thermal drawing, four 25-μm diameter tungsten wires are fed into the preform. Hundreds of individually addressable digital devices (square silicon microscale digital sensor and memory chips) are electrically connected in situ during the fiber drawing process, with all devices accessible on the same in-fiber digital communication bus (Fig. 31b, c). Each chip with four corner-positioned contact pads is first placed into slots within a polymeric preform and placed at angle of 26.56° with respect to the fiber axis and the slots in the preform are milled to the exact dimensions of the chips. The PMMA is chosen to be the barrier between wires and devices. Discrete in-fiber electronic devices are positioned uniformly at different spatial positions along the fiber. Each device offering different functions such as sensing, data storage, or storage of in-fiber algorithms. By sending a predetermined serial combination of digital 1 and 0, the device with the correct matching digital address along the fiber can be switched ‘on’ to activate its internal functionality including memory or sensing modalities (Fig. 31d). To realize the information interaction, the I2C protocol was implemented into the fiber. The equivalent logic circuit of each device within the fiber is composed of XNOR and AND gates. The digital fibers are also thin and soft enough to be passed into a needle and sewn into textiles (Fig. 31e–g). The fiber permits for large memory storage in a single strand of fiber. A 767-kilobit full-color (red–green–blue) 8-frame movie file can be stored within a meter of fiber stored for 2 months without power. A digital fiber, composed of a hybrid of memory and temperature-sensing functionalities along the same strand, was fabricated. The fiber temperature sensor (thermistor device) is in direct contact with the skin of the armpit. Body temperature measured every 0.5 s is converted from analog-to-digital signals and communicated to the fiber memory to store the temperature under different physical activities: sitting, standing, walking, and running. The body temperature dataset is then used to train a neural network to detect and classify four distinct activities (sitting, standing, walking, and running). To train this network, ~ 1800 data sections of temperature values, each spanning 12 s corresponding to the four classes, are provided as input into a CNN. The CNN is optimized to provide a high training accuracy (average of 97.9 ± 0.7%). After training, the values of the weights and biases are extracted and reduced to produce a compressed neural network, including mathematical equations for feature selection, weights, biases, and ReLU functions (1650 neuronal connections), which are all stored into the digital memory of the fiber.

### Brief Summary

Assisted by the machine learning algorithms, the physiological signals introduced above can be not only monitored but also diagnosed by the soft electronics. Up to now, many algorithms such as PCA, LDA, FT, WT, GNB, SVM, DTW, kNN, K-means, DT, FNN, CNN, RNN, etc., have been demonstrated to coordinated with the soft electronics. Limited by the database size, the advantage of deep learning algorithms especially neural network has not been fully utilized. In some works, classic algorithms even have better performance than the neural network. As the table we summarized, the function of the most of algorithms are classification, which is a qualitative analysis. More qualitative analysis based on the regression models should be studied.

The soft electronic can be used to build large-scale database, which containing more physiological information to analyze. In addition, most of the current researches are based on the supervised learning. The complex data calibration process is inevitable. More unsupervised learning model should be further studied. For the better wearing experience, both front-end sensor and rear-end circuit should be designed in soft form. For the larger and more correct database, the interface between sensor and circuit should be optimized to decrease the noise, and the interface between soft system and skin should also be designed. With the development of soft electronics, the powerful function of neural network can be fully taken. With the development of the microcontroller unit, more chips can support the in situ operation of machine learning algorithm, which are suitable to the small-scale algorithms (Table 5). How to choose the operation method and chips depends on the algorithm size and power dissipation. In addition, during practical application, the interface between the hardware and algorithms in situ or on cloud should also be designed.

**Table 5:** Typical chips supporting algorithm implantation

Type	Core	Frequency	Memory	Computation	Website link
STM32WB55	Arm 32-bit Cortex-M4 CPU	64 MHZ	256 KB RAM, 1 MB FLASH	219.48 CoreMark	[367]
STM32L4S5VIT6	Arm 32-bit Cortex-M4 CPU	120 MHz	640 KB RAM, 2 MB FLASH	409.20 CoreMark	[368]
ESP32-C3	RISC-V 32-bit CPU	160 MHz	400 KB RAM, 4 MB FLASH	407.22 CoreMark	[369]
ESP32-S3	Xtensa Dual-core 32-bit LX7 CPU	240 MHz	512 KB RAM, 8 MB FLASH	1181.60 CoreMark	[370]
BCM2711	ARM Quad-core Cortex-A72 64-bit CPU	1.5 GHz	8 GB RAM, 1 MB L2 CACHE	15,600 CoreMark	[371]
nRF52840	ARM Cortex -M4 32-bit CPU	64 MHz	256 KB RAM, 1 MB FLASH	212 CoreMark	[372]
Kendryte K510	Tripe-core RISC-V 64-bit CPU	800 MHz	4 GB eMMC, 128 M FLASH	3 TOPS	[373]

## Challenge, Outlook, and Conclusions

### Soft Neuromorphic

To some extent, the organism can be regarded as a neural network. The initial neural network information was saved in the gene. The living of organism is a transfer learning process. The organism was “told” the “label” to know the correct answers and got the feedback to train its own neural network. Finally, the new neural network was passed the information to the next generation for the next transfer learning process. In addition, the SNN discussed above mimic the performance of the nerve cell. Therefore, the soft electronics have great potential in the HMI and neuromorphic field. The soft HMI is usually noninvasive can attached to the skin, where the soft character can realize the tight contact with the skin and further improve the SNR. The HMI application has been discussed above. Therefore, the neuromorphic will be introduced below, which can link the organisms and circuits.

An important application of the soft HMI is the intelligent prostheses. However, the signals picked by sensors are not suitable to the nerves. Therefore, the signals need to be processed by the ICs chip. Besides, there are complex connections between circuits and sensors. A solution of this problem is to fabricate neuromorphic devices with the sensors together in the same substrate. The sensors are connected to neuromorphic devices which can convert the mechanical or optical signals detected by sensors into the neural signal like a synapse. In the synapse, the signal amplitude and frequency are controlled by the neurotransmitter emitted by the presynaptic membrane to synaptic space and accepted by postsynaptic membrane. Besides, there are many neuromorphic characters such as long-time potentiation (LTP), long-time depression (LTD), paired pulse facilitation (PPF), spike-timing-dependent plasticity (STDP), etc. For the soft neuromorphic devices, they generally divided into two kinds, memristor (or RRAM) and synapse transistor. Some integrated sensor and neuromorphic device system are summarized in Table 6.

**Table 6:** Typical biomimetic systems

Bionics object	Substrate	Sensor	Sensitive material	Cascade device	Neuromorphic device	Neuromorphic material	Application	References
Somatosensory system	SEBS	Pressure	CNT/Au	Organic ring oscillator	Synaptic transistor	Pentacene/Ion gel liquid	Driving cockroach leg	[378]
Tactile sensory organs (Merkel cells)	PI	Pressure	Pentacene/BT NPs/P(VDF-TrFE)	None	Synaptic transistor	Pentacene/BT NPs/ P(VDF-TrFE)	Expecting touching order	[380]
Human visual system	PI	UV detector	In_2_O_3_ SMWs	None	Memristor	Ni/Al_2_O_3_/Au	UV imaging and pattern recognition	[382]
Haptic memory system	PI	Pressure	AgNWs/PDMS	None	Memristor	Ag/SiO_2_/Au	Reconstructing pressure distribution	[381]
Stretchable memory system	PDMS	Strain	Au	None	Memristor	Ag/ZIF-8/Au	Detecting and recording joint movement	[384]
Light sensory synapse	SEBS	Photodetector	Organics/ZnO	Polymer actuator	Synaptic transistor	CNT/NW/Ion gel liquid	Optical driving controlling	[383]

The memristor usually has two resistive state, the high resistance state (HRS) and the low resistance state (LRS), which can be used as the memory. There is a gradually changing state between HRS and LRS. The HRS and LRS can be regarded as the “0” and “1” in the digital circuit. The process converting HRS to LRS is named “SET”. On the contrary, the process converting LRS to HRS is named “RESET”. The gradual state can be regarded as the continuous signal in the analog circuit. During the working process of memristor, the conductive filament is induced by the applied voltage. The pre-electrode, resistive layer, and post-electrode can be regarded as presynaptic membrane, synaptic space, and postsynaptic membrane, respectively. The gradual forming and vanishing of filament would increase and decrease the conductance of the memristor, which can imitate the excitation and inhibition of synapses. Up to now, many materials have been used to fabricate the memristor, such as transition metal oxide [374], TMD [95], and organic materials [375].

For the synapse transistor, the gate electrode, semiconductor channel, and source electrode can be regarded as presynaptic membrane, synaptic space and postsynaptic membrane, respectively [376]. In the metal oxide semiconductor field effect transistor (MOSFET), there is an electrical double layer (EDL) at both sides of the dielectric layer. The EDL can adjust the electron density of state and the conductance of the channel, which can influence the current of the source and drain (postsynaptic current). To improve the controlling of channel, electrolyte such as the ionic liquid can be used as the dielectric layer. There are two EDLs at electrolyte/semiconductor interface and electrolyte/gate electrode, which can have a much higher gate capacitance. Therefore, the gate signal can control the channel better [377].

**Fig. 32:**
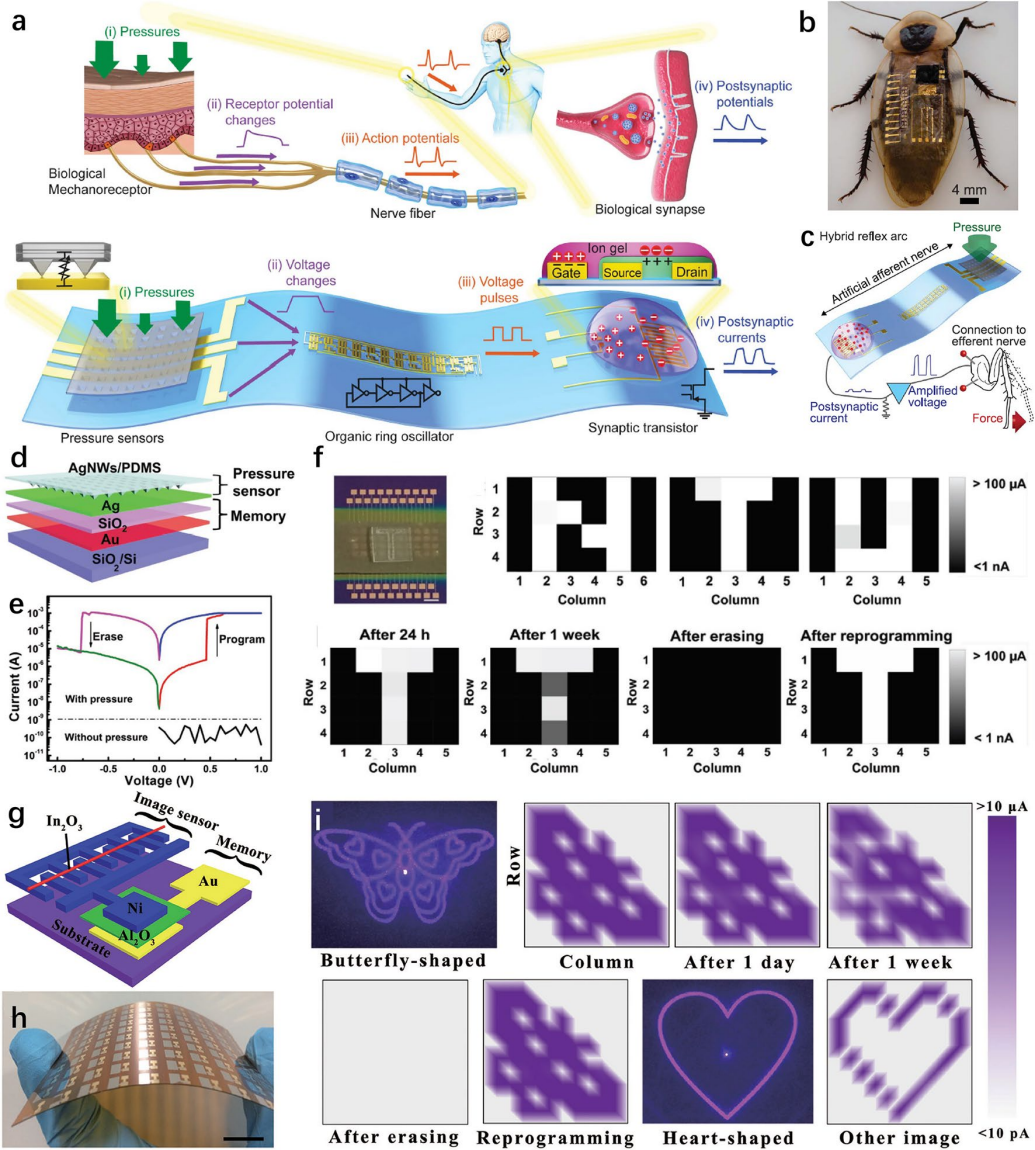
Biomimetic systems containing soft sensor and neuromorphic devices. **a** A biological afferent nerve stimulated by pressure and corresponding an artificial afferent nerve made of pressure sensors, an organic ring oscillator, and a synaptic transistor. The parts with the same colors in (upper) and (lower) correspond to each other. **b** Discoid cockroach with an artificial afferent nerve on the back. **c** Hybrid reflex arc with the artificial afferent nerve. Pressure stimuli from sensors can be converted into postsynaptic currents. Postsynaptic currents are amplified to stimulate biological nerves and cockroach muscles to initiate movement. Reproduced with permission [378]. Copyright (2018), American Association for the Advancement of Science. **d** Diagram of the haptic memory device comprising of pressure sensor and memristor. **e** Typical* I–V* curves of the integrated device with pressure and without pressure. The device can only be programmed and erased with the pressure applied. **f** Photograph of the mold (letter “T”) put on integrated haptic memory arrays. Scale bar represents 1 mm. Mapping of letters “N”, “T”, and “U”. Only device cells beneath the letters can be programmed. Demonstration of the device arrays to memorize the applied pressure and multicycle usage. Reproduced with permission [381]. Copyright (2016), Wiley–VCH. **g** Diagram of the bioinspired visual memory unit consisted of an image sensor and a memristor. **h** Photograph of the integrated devices arrays on soft PI substrates. Scale bar represents 1 cm. **i** Information storage performance and effective reusability of the soft visual memory device arrays. Reproduced with permission [382]. Copyright (2018), Wiley–VCH

Kim et al. realized an artificial afferent nerve containing three parts: resistive pressure sensors array, organic ring oscillators, and a synaptic transistor (Fig. 32a) [378]. The pressure sensors are composed of a conducting pyramid-structured elastomer and Au electrodes. Higher pressure would increase the contact area and decrease the resistance. Then, the sensors are connected to an organic ring oscillator, which can convert tactile stimuli into voltage pulses. The output frequency of the oscillator is proportional to the input voltage. In addition, the oscillator performance is similar to the mechanoreceptor of the human skin [379]. Finally, the oscillator is connected to the ion gel-gated synapse transistor. They connected the artificial afferent nerve to biological efferent nerves of a discoid cockroach (Fig. 32b) to realize a biological reflex arc. The pressure sensors can lead to the actuation of the tibial extensor muscle in the cockroach leg (Fig. 32c). Lee et al. mimicked the Merkel cell function in human skin by a soft ferroelectric organic field effect transistor (Fe-OFET). The gate dielectric is barium titanate NPs and poly (vinylidene fluoride-trifluoroethylene). This single soft transistor can convert the mechanical energy to potential like the Piezo-2 channels in Merkel cell [380].

Zhu et al. demonstrated haptic memory devices for the mimicry of human haptic memory [381]. They integrated memristors with resistive pressure sensors, where the resistance states in a memristor can be recognized. By applying pressure on the pressure sensor. The pressure sensor is based on pyramid-structured PDMS film embedded with AgNWs as sensitive layer. Then pressure sensor is on the top of the Ag/SiO_2_/Au memristor (Fig. 32d). When there is no pressure, the device has a very high resistance because both the pressure sensor and the memristor are in the HRS. When the pressure is applied, the memristor can be switched from HRS to LRS by a sweeping voltage (Fig. 32e). These haptic memory devices can map and memorize the external pressure distribution, when different letter molds of “N”, “T”, and “U” applied on the device arrays (Fig. 32f). The arrays can retain the pressure distribution for a week with little decay, and the pressure information can be easily erased by the voltage sweep.

In addition to the mechanical memory devices, Chen et al. designed an artificial soft visual memory system by integrating a ultraviolet (UV) image sensor and a memristor, where top electrode of the memristor is replaced by one electrode of the two-terminal image sensor (Fig. 32g, h) [382]. The UV image sensor is based on the In_2_O_3_ semiconductor micrometer-sized wires fabricated by printing process, and the memristor is based on the Ni/Al_2_O_3_/Au architecture. The system can be applied to detect the UV laser pattern and recorded it. The pattern can retain in the visual memory array for 1 week with little attenuation. Besides, the state of memristor can also be erased. Lee et al. realized a light sensory synapse, consisted of an organic photodetector (OPD), a stretchable organic nanowire synaptic transistor (s-ONWST), and a polymer actuator [383]. The optical signals detected by the OPD and processed by the s-ONWST can be applied to drive the actuator.

In all, the soft neuromorphic can be the hardware form of neural network. The sensor, the processor, and the memory can be integrated into a single device, which can improve the computational efficiency and decrease the power dissipation.

### Soft Integrated Circuit Hardware for Algorithms

One of the most important inventions of twentieth century is the ICs. It is the fundament of the modern information technology [385]. The ICs is the hardware foundation of algorithms. As discussed in “Neural network run on soft electronics” part, the neural network has been run on the soft system. However, for the stronger computing power, the computing device density of the soft system should be further improved. The past decades have witnessed the explosive development of ICs following the Moore’s law [386]. However, in the recent years, due to the leaky current in the MOSFET, it is hard to decreasing the feature size of silicon transistor in the nanometer level. At the meaning time, some new materials show the potential in the next-generation transistor. With the 1D SWCNT as the gate and 2D MoS_2_ as the semiconductor channel, the gate length of the transistor can be 1 nm [387]. With the single-layer graphene edge as the gate and 2D MoS_2_ as the semiconductor channel, the gate length of the transistor can be as small as 0.34 nm [388]. With the s-SWCNT as channel, the gate length of the CMOS can be down to 5 nm. ICs Chips based on the SWCNT has also been developed, which can be used to the CNT computers [66, 389] and sensor system [390]. Although found later than CNT, 2D semiconductor like MoS_2_ can also be applied in the ICs chips. Polyushkin et al. designed a operational amplifier circuit based on the MoS_2_ [391].

**Fig. 33:**
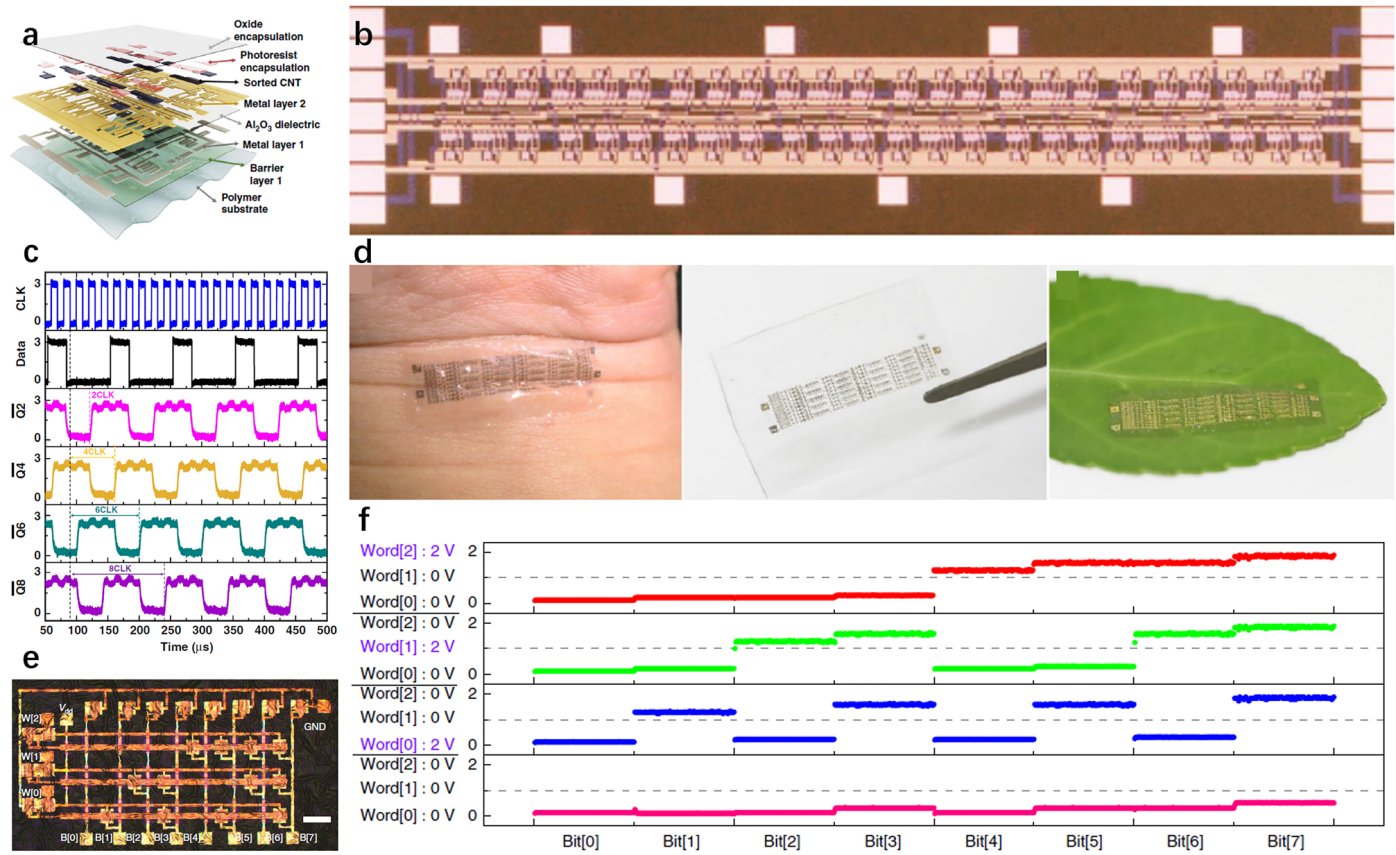
Soft IC. **a** Layer structures of the CNT TFT. **b** Photograph of an eight-stage SR containing 304 CNT TFTs. **c** Measured waveforms of the eight-stage SR with CLK at 50 kHz and input data at 10 kHz. Reproduced with permission [392]. Copyright (2019), Springer Nature. **d** Images of the soft electronic devices transferred onto a wrist, a biodegradable PVA film, and a plant leaf. **e** Photograph of a NAND-ROM. Scale bar represents 200 μm. **f** 8-bit readout voltage of NAND-ROM under different 3-bit addresses of ‘000’, ‘001’, ‘010’ and ‘100’. Reproduced with permission [393]. Copyright (2018), Springer Nature

Due to the flexibility of nanomaterial, it also has the advantage than silicon in the soft electronics. Up to now, many soft transistors based on the nanomaterials have been proposed. The integration and ICs design may be the next step. The soft ICs chips will solve the problem that the mismatch of interface between the soft sensor and rigid chip, and really realize the soft system. Lei et al. reported a series of soft ICs devices based on the s-CNT with an ultrahigh selectivity of 99.997% and a high sorting yield of 19.9% (Fig. 33a) [392]. By increasing CNT density, the mobilities of CNT TFT can be up to 49 cm^2^ V^−1^ s^−1^, however with larger device variations. With the pseudo-CMOS design style, they realized basic logic gates, including inverters, NAND, and XOR logic gates, five-stage ring oscillators running up to 3.5 MHz, and a tunable-gain amplifier with voltage gain of 1000 at 20 kHz. Based on the positive edge triggered D flip-flop (DFF), they realized an eight-stage shift register (SR) containing 304 CNT TFTs operating at 50 kHz clock rate (Fig. 33b). Figure 33c shows the measured waveforms of the SR. This work has the potential in wearable or IoT applications. Xiang et al. also realized CNT-based TFTs and ICs with bio-integration capability [393], which can be transferred to arbitrary surfaces such as a wrist, a biodegradable PVA film, and a plant leaf by wet and dry approaches (Fig. 33d). The statistical distribution of the mobility The CNT TFTs is 48.9 ± 7.8 cm^2^ V^−1^ s^−1^. Based on the CNT TFTs and inverters, basic PMOS logic gates such as NOR and NAND gates were fabricated. A half-adder was realized, which consisted of 18 TFTs and 7 basic logic gates. In addition, A NAND read-only memory (ROM) was constructed using 29 transistors to map a 3-bit address input onto 8-bit data output values (Fig. 33e). The data output read from the NAND-ROM circuit shows correct logic values (Fig. 33f).

**Fig. 34:**
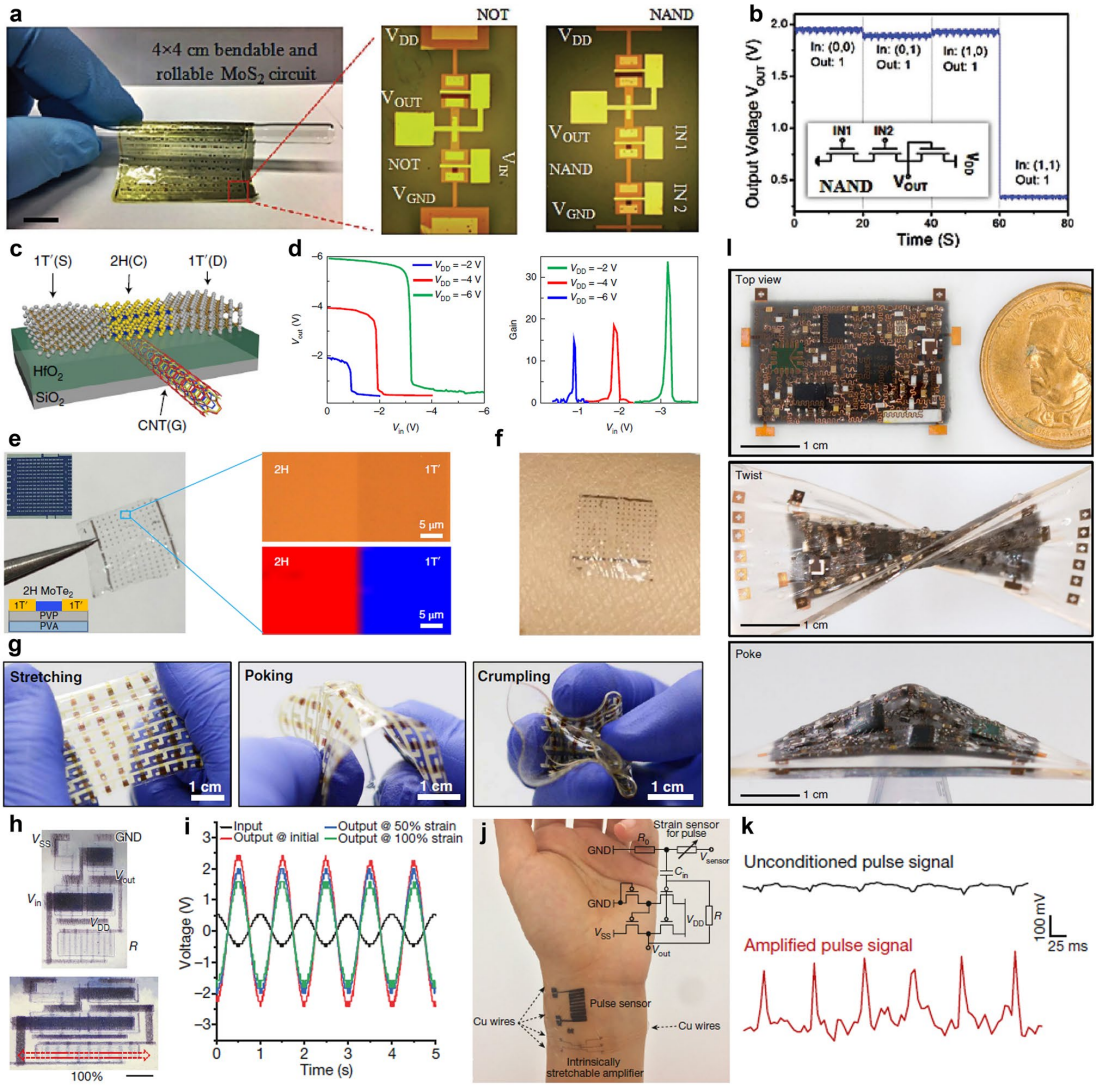
Soft ICs. **a** Photograph of the rollable logic circuits wrapped around glass pipette together with magnified NAND and NOT structure. Scale bar represents 1 cm. **b** Schematic and measurement results of the NAND gates on the PI substrate. Reproduced with permission [394]. Copyright (2018), Wiley–VCH. **c** Schematic of a 1T/2H MoTe_2_ FET with a CNT gate. **d** Transfer characteristics of the inverter operating at *V*_DD_ =  − 2, − 4, and − 6 V. Gains of the inverter for the same vales of V_DD_. **e** Self-supporting substrate with an array of 144 stretchable transistors. Insets: photograph of the device array on SiO_2_/Si substrate before peeling-off (top) and cross-sectional schematics of the devices (bottom). Optical and Raman mapping image of a 1T/2H MoTe_2_ junction on PVP/PVA film, respectively. **f** MoTe_2_ devices with PVP/PVA substrate on skin. Reproduced with permission [395]. Copyright (2019), Springer Nature. **g** Transistor arrays under different mechanical deformation such as stretching, poking, and crumpling. Reproduced with permission [396]. Copyright (2019), American Association for the Advancement of Science. **h** Optical microscope images of a stretchable amplifier in initial state (top) and stretched to 100% strain (bottom). **i** Input and output signals after amplification at 0%, 50%, and 100% strain. **j** Stretchable amplifier applied to amplify arterial pulse signals measured by a stretchable strain sensor. The devices are attached on skin side-by-side. **k** Pulse signals before and after amplification. Reproduced with permission [398]. Copyright (2018), Springer Nature. **l** Optical micrographs of the system when freestanding (top), twisted (middle) and poked (bottom). Reproduced with permission [399]. Copyright (2018), Springer Nature

TMD materials can also be used to fabricated the soft ICs. Shinde et al. developed a water-assisted method of transferring wafer-scale MoS_2_ films, where the ultrathin PI film (1.5 μm) can be the carrier layer and substrate at the same time for a soft device [394]. The rollable integrated circuit consisted of inverter, NAND, and NOR can be embedded on a glass pipette (Fig. 34a). Besides, the performances of the FETs on the PI were similar to those on Si substrate. The performance of NAND gates based on MoS_2_ transistors on a PI substrate shown in Fig. 34b, and the output functionalities were measured in flat states. There are usually two typical phase, semiconducting hexagonal (2H) and metallic monoclinic (1T) phase. Zhang et al. chemically synthesized 2H and 1T MoTe_2_ using MoO_2.0–2.5_ and MoO_3_ thin films at 650 °C in a single step (Fig. 34c, d) [395]. 2H phase was applied as the semiconducting channel and 1T phase used as the source and drain, which can realize the seamless connection (Fig. 34e). The transition voltage of MoTe_2_ inverter approached the ideal voltage (*V*_DD_/2) and the voltage gain can be about 35. Compared with traditional process, the one-step fabrication of FETs can avoid interfacial contaminations and material degradation without the post-synthesis lithographic process. The device array can be transferred to the PVP and PVA thin films as the mediator and obtained a freestanding thin film with device arrays (Fig. 34f).

Compared with the inorganic semiconductor, the organic semiconductors are not only flexible but also stretchable. Sim et al. developed fully rubbery integrated electronics and logic, which can still work when stretched by 50% [396]. The metallic CNT (m-CNT) doped P3HT–nanofibrils (NFs) is exploited as the rubbery semiconductor. AuNPs–AgNWs/PDMS elastomeric conductor was applied as the source and drain electrodes, and ion gel was used for the gate dielectric. Due to the rubbery character of the materials, the transistor array can sustain mechanical deformations without any physical damage, such as stretching, poking, and crumpling (Fig. 34g). The fully rubbery logic gates, including inverters, NANDs, and NORs were also realized. Wang et al. developed transistors based on conjugated polymer/elastomer phase separation induced elasticity, which can still work under 100% strain only with little shift in the transfer curve. 6300 stretchable transistors can be integrated on an area about 4.4 × 4.4 cm^2^. A digital NAND gate and an analog amplifier circuit are realized by the stretchable transistors, both can still work under 100% strain (Fig. 34h, i). Finally, an on-skin amplifier was used to be applied to amplify raw detected physiological signals successfully (Fig. 34j, k). Kwon et al. developed an approach to integrating soft organic transistor 3D monolithically [397]. A large-scale soft logic circuitry was realized using a 12 × 8 3D NAND gate array. In addition to the MOS-level, the soft ICs can also be more integrated into the PCB-level. Huang et al. developed a multilayer PCB system (Fig. 34l). The interlayers were connected using vertical interconnect accesses formed by laser ablation.

With the development of soft semiconductor materials such as CNT and TMD, and the fabrication process, the function single soft CMOS transistor and integration of the soft ICs can be further improved. The ability of machine learning algorithm to process physiological signals in situ will be greatly enhanced.

### Microfluidic Channels for Collecting Chemical Signals

The signals and devices discussed above are most physical physiological signals. In addition, most of the algorithms cooperated with soft electronics reviewed above only analyze single signal. Considering the complexity of human body, only single signal, even only physical signals cannot fully reflect the state of human body. When we go to the hospital for physical examination, chemical signals are also indispensable. Therefore, the soft system integrating physical and chemical signals can obtain more information about human body. In addition, assisted by the powerful machine learning algorithms, the potential information hidden in multimodal signals can help the diagnosis of the disease.

The chemical physiological information is usually saved in the body fluids, such as sweat [400], tears [401], saliva [402], and interstitial fluid [403]. Among these body fluids, due to the easy to collection, sweat has been studied most. Compared with the physical physiological signals, the chemical physiological signal is easier to be contaminated during the sample collection process. Microfluidic channels can be used to collect the fluids, and wildly used in the biochemical analysis field.

Sweating is the basic biological activity of the human body, which plays an important role in keeping the stability of the internal environment. There are many functions of the sweat. Heat can be taken away from the body surface through the evaporation of sweat, thus maintaining a constant body temperature. Some metabolism and toxins can also be excreted by sweating.

Sweat has complex components. In addition to water, the most abundant substance in sweat is NaCl. Some trace elements such as Ca, Zn, and Mg can also be found in sweat. Besides, amino acids, hormones, urea, uric acid, lactic acid, glucose, and creatinine are also the components of sweat. Therefore, much human health information can be obtained by analyzing sweat, such as electrolyte imbalance, glucose level, lactic acid index, dehydration status, and calorie burning value [404, 405]. Compared with the blood analyzing, it is a noninvasive and in vitro method, which makes it more convenient and healthier.

**Fig. 35:**
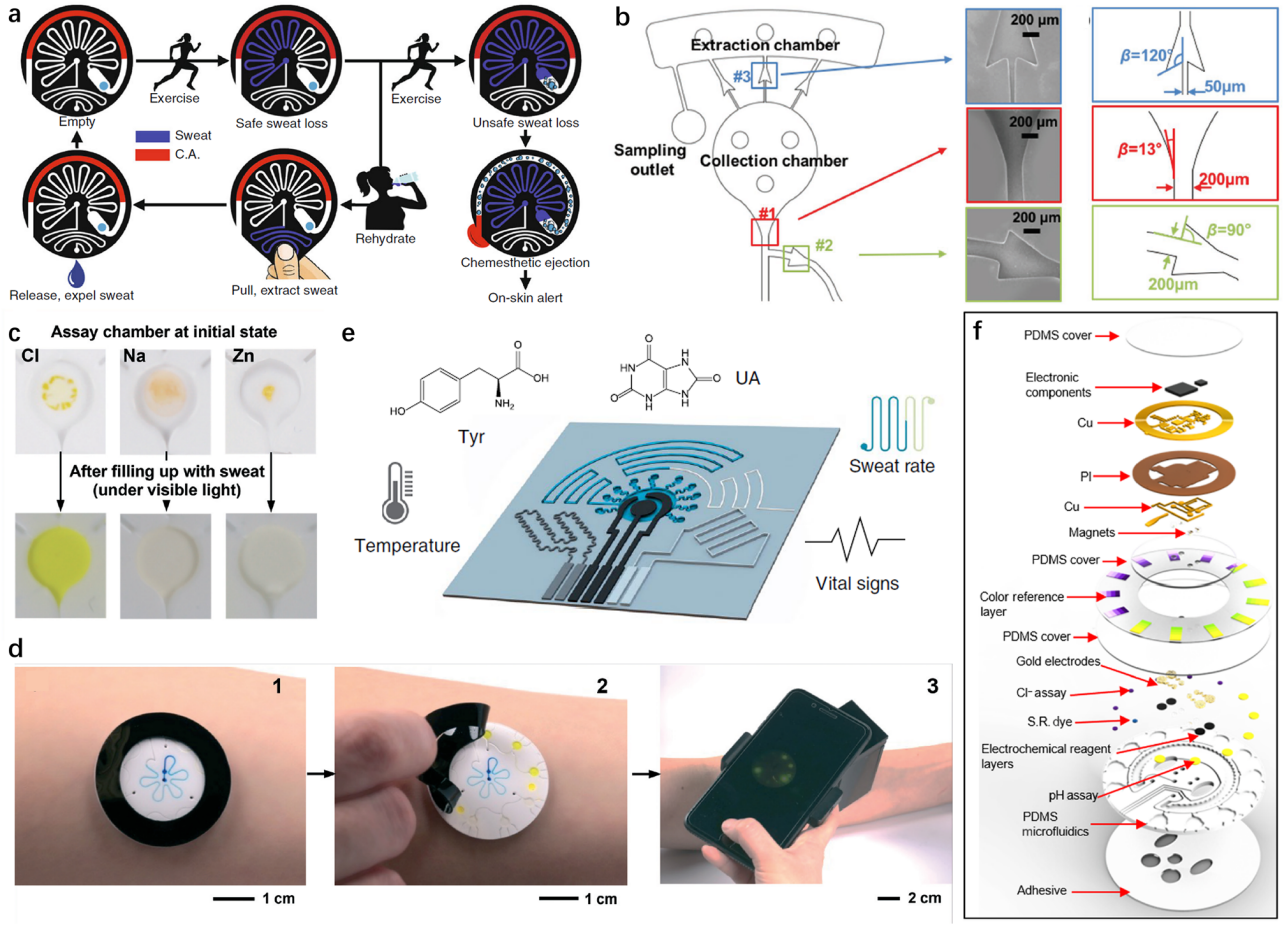
Soft sweat sensor. **a** Schematic flow of reusable microfluidic channels operation. C.A. = Chemesthetic agent. Reproduced with permission [406]. Copyright (2019), Nature Publishing Group. **b** Schematic illustration of a unit cell, containing a collection chamber, extraction chamber, sampling outlet, and three CBVs. SEM images of CBVs. Sketch of CBVs with indicated channel width and diverging angle. Reproduced with permission [409]. Copyright (2017), Wiley–VCH. **c** Photographs of the microreservoirs for the assays before (upper) and after (lower) filling with sweat collected by microfluidic channels under visible light illumination. **d** Procedure for performing a fluorometric assay: 1. Collecting sweat by skin-interfaced microfluidic device 2. Peeling away the black shield 3. Capturing a photograph of the device using a smartphone with the optics module. Reproduced with permission [412]. Copyright (2018), Royal Society of Chemistry. **e** Multiple functions of the laser-engraved sensor: sweat UA and Tyr detection, sweat rate estimation, temperature sensing, and vital-sign monitoring. Reproduced with permission [414]. Copyright (2020), Nature Publishing Group. **f** Schematic illustration of the exploded view of the hybrid battery-free microfluidic channels system. Reproduced with permission [415]. Copyright (2019), American Association for the Advancement of Science

To detect the sweat loss of human body, Reeder et al. developed a soft microfluidic device [406]. When the sweat loss exceeds a threshold, the sweat can trigger to release menthol or capsaicin solution as an alert (Fig. 35a). By designing microstructured features of microfluidic surface and strain-actuated elastomeric suction pump and elastomeric pinch valve, the sweat in microchannel can be pulled out, if the sweat loss was not across the threshold, which makes the device reusable.

To analyze the components of sweat, there are usually two common methods, colorimetric and electrochemical method. However, a problem should be solved before the analysis. The tested sweat is easy to be contaminated by oils, dirt, and chemicals of skin and environment. In addition, how to prevent the interference between sweat excreted at different time is also a problem. The microfluidic channel is an effective way to collect fresh sweat [407, 408]. Choi et al. designed capillary bursting valves (CBVs), which can direct the sweat to fill the microreservoirs in a sequential manner (Fig. 35b) [409]. the computed bursting pressures for CBVs #1, #2, and #3 are 498.9, 881.7, and 3035.7 Pa, respectively. When sweat arrives at CBVs #1 and #2, CBV #1 will open and allow sweat into the chamber. After filling the chamber, the sweat flow will burst CBV #2 at sufficient pressure to fill the next chamber. After finishing the collection, the device was inserted into the centrifuge to open CBV #3 and move the sample into extraction chambers for lab analysis. Finally, the sample was analyzed by chromatography–mass spectrometry system.

Colorimetric method is based on the reaction between sweat components and color indicator to produce color change, and realize the detection of the tested substances [410, 411]. The color change is mainly recognized by human eyes. Therefore, it is not accurate enough. However, colorimetric method is much easier in special field, such as the fast test. Sekine developed a fluorometric sweat sensor which have three components: an adhesive layer, a platform of microfluidic channels, and valve structures to excrete sweat to microreservoirs (Fig. 35c, d) [412]. The microreservoir contains fluorescent assays tailored for Cl, Na, and Zn. A detachable light-shielding layer is fabricated to prevent exposure of fluorescent reagents to light before the readout process. A smartphone-based fluorescence-imaging modules was built to analyze the fluorescent performance. In addition to the components, the sweat loss can also be measured by the flower-shape microfluidic channels.

Compared with colorimetric method, electrochemical method has better sensitivity and accuracy. By modifying the selective electrode, the selective recognition and detection of physiological markers in sweat can be realized by detecting the potential difference, current and resistance signals between the electrodes [413]. Yang et al. realized the simultaneous sweat sampling, chemical sensing, and vital-sign monitoring by a laser-engraved sensor (Fig. 35e) [414]. LIG was used as the active material of temperature and strain sensor to detect temperature and respiration rate. Microfluidic channels pattern was engraved directly on the PET substrate by laser. LIG was also applied as the electrochemical electrode to analyze the sweat collected by the microfluidic channels. Low-concentration uric acid and tyrosine can be detected to prevent diseases such as gout. Bandodkar et al. realized a battery-free, wireless electronic sensing system, which can simultaneously monitor sweat rate/loss, pH, lactate, glucose, and chloride (Fig. 35f) [415]. The sweat collection and loss rate were measured by microfluidic channels. Lactate and glucose were detected by electrochemical method. PH and chloride were detected by colorimetric method. Besides, an NFC-based system can harvest energy from mobile phone and read data. This system has good long-term stability for more than 2 days.

Machine learning algorithm can efficiently find the relationship between various signals. The more the signal types, the more powerful the algorithms. Therefore, by combining physical and chemical signals, more useful information for the health care can be mined.

### Challenge and Outlook

The applications and advantages of soft electronics assisted by algorithms have been demonstrated above. However, many challenges still need to be overcome.

In the material level, the repeatability and yield of the nanomaterial should be further improved. For example, the well-aligned and all-semiconducting SWCNT films with high uniformity, high array density, and low defect density on large wafers are urged. Improving the quality and area of graphene thin films and finding better ways to transfer it are important for its further applications. For the wearable device, the safety and comfort are the most important parameters. The preparation process of some organic materials and heavy metal usually requires the toxic solutions, etc., which may be harmful to the human organisms. Therefore, how to improve the biocompatibility of soft material is important, and the biocompatibility experiment should be paid more attention. In addition, for the wearable device, the interaction between materials and skin or excreta should be discussed in detail in the future.

Although the soft electronics has been developed for many years. Most of the soft devices and system are still based on the dense polymers (PDMS and Ecoflex), which makes the sweat hard to evaporate and limit the metabolism of skin. When worn for a long time, there will be inevitable redness and inflammation. The uncomforting performance of these devices will hinder the development of soft electronics. Therefore, the morphology of material or structure should be designed to let the water and gas cross. In addition, the dense polymers usually have thickness in millimeter, which will influence the interface between the device and skin [287]. The nanomesh has potential to the solve the problem. In addition, for the current breathable sensor, only the sensor is breathable, while the circuit and other part are not. It is urgent to realize a permeable system. Besides, the interface between soft sensor and circuit should be optimized to obtain a high SNR. The ideal solution is the fully soft system consisting of the sensor, interconnection, and chips.

For the soft electronics combined with algorithms, there are still some points need to be further optimized in software and hardware. In terms of software, first, more industry-standard signal dataset suitable to the soft electronics should be built. This can avoid much redundant work and provide a standard to judge the performance of devices and algorithm. Second, the test method of physiological signals should also be standardized. Third, more special algorithm should be proposed to further widen the application field of machine learning in the soft electronics. For example, limited by the device density in the soft system, small-scale algorithms are tended to be chosen. Fourth, the system combining physical and chemical signal can provide more information about the physical condition. Therefore, the synergetic algorithm to analyze the multimodal signal is necessary. Fifth, to fully taken the powerful function of neural network algorithms, many novel neural networks such as Yolo, Faster R-CNN, and transformer should be combined with the soft electronics to realize more functions, for example multiple-target colorimetry recognition. In terms of hardware, the density of computing device in the soft system should be increased, which can run the larger-scale algorithm in situ. To realize more powerful soft system, advanced soft material and fabrication process should be studied.

The health care process can be divided into two types, health monitoring and real-time alert. Both of them can be realized by the machine learning-assisted soft electronics. The tight interface and good wearing experience can enlarge the size of dataset, which can provide more information to diagnose and make a more accurate monitoring process. For the real-time alert, the characteristic signal can be distinguished timely or even ahead of time. Taking the ECG as an example, users wear the ECG sensor the monitor their ECG signals. In traditional chest pain centers, medical personnel can read the ECG of users. If the abnormal signal appears, medical personnel can give warning timely by the medical personnel. However, with the increasing of users, more medical personnel are needed, which leads to larger economic pressure and social pressure. In addition, most of the physiological signals are normal signals, and the waste of resources is inevitable. The machine learning-assisted soft electronics can solve this problem, and relieve the medical pressure mentioned above. To improve accuracy of the diagnosis, the correctness of the label is important. Before the practical application, more experienced experts should be invited to label the data. Based on this, the diagnostic capability of the intelligent soft system can achieve the expert level in the future.

For the soft electronics combined with the neuromorphic devices, the HMI has the huge potential. Many sense organs such as eye and skin can be reconstructed. Besides, the prosthesis based on the soft neuromorphic system can recover the motor function. To realize the artificial prosthesis, more devices should be demonstrated to realize more neuromorphic functions. The electronics should be combined with physiology in the future.

For the soft ICs, the analog and digital circuit all have huge improvement. The basic amplifier has been realized to the analog signal amplification, and basic digital logic gates have been applied to realize the Boolean operation. However, the soft large-scale integration and very large-scale integration have not been realized yet. Some modular such as ADC and Bluetooth are indispensable to the totally soft system.

In conclusion, a comprehensive review about the soft electronics system has been demonstrated which consists of material, physiological signal, and machine learning algorithm. Some nanomaterials such as CNT, graphene, and AgNWs have been discussed in detail. Characteristics of different physiological signals corresponding devices have been introduced. Then, the intelligent soft system powered by the machine learning algorithm has been discussed carefully. Finally, the challenge and outlook of the intelligent soft system based on nanomaterial has been stated. With the help of the machine learning, the soft system can detect and diagnose physiological signal, simultaneously. The diagnosis and prevention of many diseases can be carried out during the daily life, which can greatly relieve medical pressure and decrease the physical examination cost.
